# Supplementary material for: The role of community engagement in promoting research participants’ understanding of pharmacogenomic research results: Perspectives of stakeholders involved in HIV/AIDS research and treatment
Source: PLoS One. 2024 Apr 2;19(4):e0299081. doi: 10.1371/journal.pone.0299081 (PMC10986979; doi:10.1371/journal.pone.0299081)
Supplement: S1 Dataset — (DOCX) [file pone.0299081.s001.docx]

|  | Coding Summary By Code | | | | | | | | | | | | | | | |  |  |  |
| --- | --- | --- | --- | --- | --- | --- | --- | --- | --- | --- | --- | --- | --- | --- | --- | --- | --- | --- | --- |
|  | | THE ROLE OF COMMUNITY ENGAGEMENT IN PROMOTING UNDERSTANDING OF INDIVIDUAL PHARMACOGENOMICS RESEARCH RESULTS..  ***CAB members’ perspectives*** | | | | | | | | | | | | | | |  |  |  |
|  |  | | | | | | | | | | | | | | | |  |  |  |
|  |  | | | | | | | | | | | | | | | |  |  |  |
|  | **Nodes\\Community engagement\Myths and misconceptions** | | | | | | | | | | | | | | | |  |  |  |
| Reports\\Coding Summary By Code Report | | | | | | | | | | Page 164 of 409 | | | | | | | |  |  |
| 02/03/2022 02:43 | | | | | | | | | | | | | | | | | |  |  |
|  | | | **Aggregate** |  | **Classification** |  | **Coverage** |  | **Number Of Coding References** | |  | **Reference Number** |  | **Coded By Initials** |  | **Modified On** |  |  |  |
|  | | | | | | | | | | | | | | | | | |  |  |
|  | | | | | | | | | | | | 2 |  | AT |  | 01/03/2022 10:23 |  |  |  |
|  | P6: About the issue of releasing results like you have said; the truth is getting advice from someone especially if they are not a professional in that field, is very dangerous. Because most people have myths, so many facts they do not know about; you find that one says 'HIV kills' but does not know how to handle the disease. I believe it is dangerous to seek advice from people and there are so many people that do this; they advised each other and said whoever suffered from HIV was bewitched. It wasn’t the HIV+ patient that says this, but the people in the community that they sought advice from, are the ones that gave the wrong information. Those people exist, those who pose risks in regard to that issue. Thank you very much.  P1: Thank you Ma'am. About seeking advice from people; I wouldn’t second it due to personal reasons—or I would say it depends on the health condition of the patient. I will look at my personal experience; the person I asked for advice is the one who almost killed me because whenever anyone came to see me, she says, "Did you know that this person is going to die? She was found HIV+, she is going to die". So some people from whom we seek the advice, are the ones that cause the patient to die. But if the participant of the research is able to understand, just prepare him/her and they will disclose the results to someone they trust. Because one might be my relative but I do not trust him/her, and my other friend who is elsewhere is the one I feel I am comfortable sharing my status with and she is ready to stand with me in every situation. Now my relative to whom you will give the results, will just bury me because whoever comes, "That one has HIV, she is going to die" "She was diagnosed with cancer" yet the cancer patient can survive for even 5years. And one who does not have cancer or HIV, could die of an accident; we have also had COVID, but didn’t have cancer or any other disease. But this relative you told about my issues, is the one that killed me by bringing stigma. Thank you Madam.  P4: Like I said, these things are not easy; these community matters are not easy because I had my challenges; my wife was taken after I was poverty-stricken. I went to the village and said, "Let me go and hustle in my village. There is no medicine, there is nothing, I will die". But a friend of mine got me from the village and started bringing me to town, and I found a female friend who used to come to see me but I was always worried, "Why is this lady coming here? I am HIV+, why can’t she leave me alone?" But there is a certain man in the community who came and told me, "That woman I see around often lost her husband" so we might think they don’t know our status in the community, but they actually know. So when he gave me that information, I started asking my friend questions and I told her to go for an HIV test. She came back with the results and she was positive, so I told her, "Let us start from there. If you want to stay alive, you have to take medicine". So we kept progressing. One man asked me, "Did you know that the first time you met me, I asked God to give me 5more years?" I asked him how old he was and he said he was 25, "How old are you now?" he said, "I am now 45years old". "Now give thanks to God because you asked him for 5years but he gave you much more, and you are still here". So these matters are not easy, you...<unintelligible> so it depends on how you move on with your life, with God's help I believe everything will be fine. Thank you very much.  M: Thank you.  P6: What I forgot to say, just like you asked if it is good to seek for advice from people in the community. You cannot say it is good but I would say, if you are to seek for advice—if you are in research study about malaria, you know that it is the medical doctor treating malaria. So you could be participating in the research that 2 does not know about, and JCRC knows, but you are on ARVs and you don’t want JCRC to find out. Now if you participate in this research and you want to seek for advice, do not go to someone in the community; instead, come to 2 where there is a professional doctor, and tell him about the issue and what you were told. He will then help you to prepare yourself. But if you consult anyone in the community, it is very challenging; you will get worried. Thank you.  P7: Thank you for this opportunity. I think seeking for advice has 2 sides to it; it is not bad to consult, but it is not good to consult any random person. First ask yourself, 'Who should I ask?' And during counselling when testing HIV patients, you first tell someone, "The results of your blood test are between you and your doctor. And if you have any questions, first consult that very doctor, don’t just ask people because they might not have the right answer" because some answers are right and others are wrong. You might consult someone and they just have positive answers which end up misleading you, and you end up dead. So I think—I have tested and tried it. There is a young adult about 20years old, he made 21 in September; but no one at home knows that he is positive except me, and his parents who are in the village. His home village is Bujagaali, but he lives in Maganjo. However, no one at home knows that he is HIV+ because he does not disclose to them, and they only say discouraging words yet they don’t know that he is positive. But he comes to speak to me and I say, "Just ignore them". Sometimes I call his father in the village, but we are talking about the son who is here. So it is not bad to ask, but the question is, "Who am I consulting?" you might not ask anyone and instead it affects you because advice could be poisonous and also medicine. So I think you need to be conscious; 'who am I going to consult?' Consult someone who is knowledgeable. We also have—I'm not sure if it was true but I think it was true; we have our system when we are in the community. We tell someone, "If you find that you are positive, do not go around disclosing to anyone, but you just have to ask yourself, 'Who should I disclose to?' because you have to disclose to one or two people. But that girl told me it was only her father and mother that knew her status, but I shouldn’t tell anyone at home, "If I get any problem"—she even gave me her father's contact; I don’t know | | | | | | | | | | | | | | | |  |  |  |
|  |  |  |  |  |  |  |  |  |  |  |  |  |  |  |  |  |  |  |  |
|  |  |  |  |  |  |  |  |  |  |  |  |  |  |  |  |  |  |  |  |
|  |  |  |  |  |  |  |  |  |  |  |  |  |  |  |  |  |  |  |  |
|  |  |  |  |  |  |  |  |  |  |  |  |  |  |  |  |  |  |  |  |
|  |  |  |  |  |  |  |  |  |  |  |  |  |  |  |  |  |  |  |  |
|  |  |  |  |  |  |  |  |  |  |  |  |  |  |  |  |  |  |  |  |
|  |  |  |  |  |  |  |  |  |  |  |  |  |  |  |  |  |  |  |  |
|  |  |  |  |  |  |  |  |  |  |  |  |  |  |  |  |  |  |  |  |
|  |  |  |  |  |  |  |  |  |  |  |  |  |  |  |  |  |  |  |  |
| Reports\\Coding Summary By Code Report | | | | | | | | | | Page 165 of 409 | | | | | | | |  |  |
| 02/03/2022 02:43 | | | | | | | | | | | | | | | | | |  |  |
|  | | | **Aggregate** |  | **Classification** |  | **Coverage** |  | **Number Of Coding References** | |  | **Reference Number** |  | **Coded By Initials** |  | **Modified On** |  |  |  |
|  | him at all, and neither does he know me but I have his contact. If she gets any problem, I first call the father; in fact, one time she got a problem, she fell seriously ill with typhoid. But I didn’t tell anyone at home; I called the father in the village and he said, "You know, I think that girl should come" however, she had an Airtel simcard and the father had MTN. He asked me, "Do you know of anyone you trust with an MTN simcard so that I send the child money to come to the village?" He sent the money to my phone, I gave it to the child and she went to the village. She got treatment, stabilized and then came back, but the people at home; even when she gets her medicine, she says, "I don’t want to keep the pills here because those people's words discourage me a lot, but you always encourage me" and I told her, "Ohh we shall change the packaging of the medicine, and use another polythene bag" and she takes her medicine home. "Even if they see it—do not label it because I know it. Do not label it, those at home do not know it. But if they see it, it is my business". So I say it is not good to ask around, but it is good to consult a professional first. | | | | | | | | | | | | | | | |  |  |  |
|  |  |  |  |  |  |  |  |  |  |  |  |  |  |  |  |  |  |  |  |
|  |  | | | | | | | | | | | | | | | |  |  |  |
|  | | | | | | | | | | | | 3 |  | AT |  | 02/03/2022 01:31 |  |  |  |
|  | P6: About the issue of releasing results like you have said; the truth is getting advice from someone especially if they are not a professional in that field, is very dangerous. Because most people have myths, so many facts they do not know about; you find that one says 'HIV kills' but does not know how to handle the disease. I believe it is dangerous to seek advice from people and there are so many people that do this; they advised each other and said whoever suffered from HIV was bewitched. It wasn’t the HIV+ patient that says this, but the people in the community that they sought advice from, are the ones that gave the wrong information. Those people exist, those who pose risks in regard to that issue. Thank you very much. | | | | | | | | | | | | | | | |  |  |  |
|  |  | | | | | | | | | | | | | | | |  |  |  |
|  | | | | | | | | | | | | 4 |  | AT |  | 01/03/2022 10:53 |  |  |  |
|  | P6: I was still speaking. Just like you said, you could tell your grandparent because most grandparents have lived long enough to know the family problems, the children. I will give an example; most of the educated people do not believe bathing the children with 'ekyogero' <good-luck charms> and they argue that White people who do not have the same rituals but they survive. What does that mean? The people long ago who did it, did so for a reason but it might instead cause a side-effect on the child. So I would suggest that if you are to share it, share it with someone who will keep your identity discreet; they could call for a meeting and say, "If you give birth to a child with this health condition, just know that it is in the lineage" instead of saying, "So and so has this". So if it is to be shared, it should be spoken of generally and not point out a particular person. Thank you very much. | | | | | | | | | | | | | | | |  |  |  |
|  |  |  |  |  |  |  |  |  |  |  |  |  |  |  |  |  |  |  |  |
|  |  | | | | | | | | | | | | | | | |  |  |  |
|  | | | | | | | | | | | | 5 |  | AT |  | 01/03/2022 10:53 |  |  |  |
|  | P5: In addition to my colleague P6, most people waste their money on traditional healers claiming that so and so was be-witched. But after sitting the clan down or your children, you tell them, "I went to study and we were told that these conditions are genetic" you tell your children, "Do not go to traditional healers asking for goats and so on, yet you know this is a genetic condition". Yes, that is all. | | | | | | | | | | | | | | | |  |  |  |
|  | **Nodes\\Community engagement\Role of CAB members** | | | | | | | | | | | | | | | |  |  |  |
|  | | | **Files\\FGD for CAB #3_3_Transcript** | | | | | | | | | | | | | |  |  |  |
|  |  |  | Yes |  |  |  | 0.0490 |  | 6 | |  | | | | | |  |  |  |
|  | | |  |  |  |  |  |  |  | |  | | | | | | |  |  |
|  | | | | | | | | | | | | 1 |  | AT |  | 28/02/2022 19:02 |  |  |  |
|  | P3: Yes, [moderator]. As CAB members I think we need to create a relationship and by creating a relationship, that helps you to prepare that person. When you are creating a relationship with this person, you are actually trying to create some attachment to this person. So it becomes very easy for you as you prepare, as you create a relationship and prepare this person, this person is going to gain confidence from you and finally you can break any news at some point in time—not breaking it abruptly, but creating that attachment is very key as a CAB member. | | | | | | | | | | | | | | | |  |  |  |
|  |  | | | | | | | | | | | | | | | |  |  |  |
|  | | | | | | | | | | | | 2 |  | AT |  | 02/03/2022 01:36 |  |  |  |
|  | P1: For—for—you see for us as CAB members, we shall speak out these things in our community dialogues, in our uh—in our preparatory meetings, in all those interventions. But at the study site—and I come to the point of how prepared are the—how should we prepare the participant. For me it takes me up to—I'll tell you that the quality of the preparation of the participant depends on the quality of the counsellors that you have in the study site. Because you should get the best. | | | | | | | | | | | | | | | |  |  |  |
|  |  | | | | | | | | | | | | | | | |  |  |  |
|  | | | | | | | | | | | | 3 |  | AT |  | 28/02/2022 19:12 |  |  |  |
|  | So for us as CAB members, our role is to share much—as accurate as information at community level, and so that the participants who are coming to the clinic already have a glimpse—an accurate glimpse of what we expect. And then the counsellor does the job uh—of finalizing it. Over to you. | | | | | | | | | | | | | | | |  |  |  |
|  |  | | | | | | | | | | | | | | | |  |  |  |
|  | | | | | | | | | | | | 4 |  | AT |  | 28/02/2022 22:29 |  |  |  |
|  | because there is no such thing as 'one size fits all' [Hmm, hmm] it is dependent on the individuals. Because they'll tell you pointblank, "Ahh I don’t want to hear what that man is saying; he is even tough. If you try to ask him a question, he says he is in a hurry" so that person is already as blocked. So the messenger is very important. [Yeah the messenger] If the participant has built a relationship, a special relationship with the clinician, let the clinician give them that information. | | | | | | | | | | | | | | | |  |  |  |
|  |  | | | | | | | | | | | | | | | |  |  |  |
| Reports\\Coding Summary By Code Report | | | | | | | | | | Page 166 of 409 | | | | | | | |  |  |
| 02/03/2022 02:43 | | | | | | | | | | | | | | | | | |  |  |
|  | | | **Aggregate** |  | **Classification** |  | **Coverage** |  | **Number Of Coding References** | |  | **Reference Number** |  | **Coded By Initials** |  | **Modified On** |  |  |  |
|  | | | | | | | | | | | | | | | | | |  |  |
|  | | | | | | | | | | | | 5 |  | AT |  | 28/02/2022 22:47 |  |  |  |
|  | P1: [moderator]. In a typical village setting, anybody who dies has been bewitched. | | | | | | | | | | | | | | | |  |  |  |
|  |  | | | | | | | | | | | | | | | |  |  |  |
|  | | | | | | | | | | | | 6 |  | AT |  | 28/02/2022 22:48 |  |  |  |
|  | M: Hmm, exactly. And here you are telling them that you want to—  P1: I'll tell you something; my Dad died of liver-related disease. My uncle also died of liver-related disease; I am ...<cuss word deleted> scared of that and I'm actually taking tests because I know—I believe in genetics. But of all those 2 people, none of them according to the villagers [died of liver, died of something else] ...<unintelligible> The other uncle—the other guy who never liked them and that was...<unintelligible>. So when you are bringing that, the reason why I laughed, we are living in a totally different setup from maybe Europe or United States. We are living in a setup where you've died because of some other causes rather than illness even when you have clearly died of this illness. So you see it really depends on the individual who has it, because you are not going to give—even the elder you have picked may never believe— | | | | | | | | | | | | | | | |  |  |  |
|  |  |  |  |  |  |  |  |  |  |  |  |  |  |  |  |  |  |  |  |
|  |  | | | | | | | | | | | | | | | |  |  |  |
| Reports\\Coding Summary By Code Report | | | | | | | | | | Page 167 of 409 | | | | | | | |  |  |
| **Nodes\\Community engagement\Willingness to accept technologies like pharmacogenetics** | | | | | | | | | | | | | | | | | |  |  |
|  | | | **Aggregate** |  | **Classification** |  | **Coverage** |  | **Number Of Coding References** | |  | **Reference Number** |  | **Coded By Initials** |  | **Modified On** |  |  |  |
|  | | | | | | | | | | | | | | | | | |  |  |
|  |  | | | | | | | | | | | | | | | |  |  |  |
|  | | | | | | | | | | | | 10 |  | AT |  | 01/03/2022 09:06 |  |  |  |
|  | So I wanted to ask, what do you hear about technology in the communities? Do people believe in technology or not? P6.  P6: Technology especially on the side of Muslims; they don’t believe in that because they are taught different doctrines that say, "If you refuse to believe the prophet, you are condemned". An example is, there are some people I advised to go for the paternity test. Sometime back when the Muslim leader was around, there is a lady who had a child and two men claimed paternity of the child. So during that time, they asked, "Who is this woman's husband?" and they said, "It is him" and "Who is the owner of the bed?" "It is him". So he said, "I want to let you know that the owner of this bed is the father of the child". Now that is how the judgement is made in Islam— | | | | | | | | | | | | | | | |  |  |  |
|  |  | | | | | | | | | | | | | | | |  |  |  |
|  | | | | | | | | | | | | 11 |  | AT |  | 02/03/2022 01:15 |  |  |  |
|  | P6: Technology especially on the side of Muslims; they don’t believe in that because they are taught different doctrines that say, "If you refuse to believe the prophet, you are condemned". An example is, there are some people I advised to go for the paternity test. Sometime back when the Muslim leader was around, there is a lady who had a child and two men claimed paternity of the child. So during that time, they asked, "Who is this woman's husband?" and they said, "It is him" and "Who is the owner of the bed?" "It is him". So he said, "I want to let you know that the owner of this bed is the father of the child". Now that is how the judgement is made in Islam— | | | | | | | | | | | | | | | |  |  |  |
|  |  | | | | | | | | | | | | | | | |  |  |  |
|  | | | | | | | | | | | | 12 |  | AT |  | 01/03/2022 09:06 |  |  |  |
|  | So I said, "Ahh! You could go to the DNA and find out the father of the child" and he said, "You are disagreeing with the prophet!". So I insisted and said, "Religion exists and back in the day, the final verdict of the prophet is what you would go by. But the world is evolving" and there is a word in Quran that says, "Consult others about what you do not know" <Speaks Arabic>. If you fall sick of malaria, you cannot see the germ itself but you go to an expert who will make the diagnosis and tell you, "You are suffering from malaria". Therefore, we should try to accept that there are those who know what we don’t know. Therefore—especially in the Muslim society, they are so rigid about accepting technology because a Muslim might not pick a phone call...<unintelligible> they exist in society. In this day and age, a mobile phone is necessary, it is a need and they just say, "Don’t tell me that!" So we still have that challenge especially among Muslims; ignorance is still high. Therefore, telling people in the community about technology requires thorough teaching in order for them to understand. Also giving examples for instance last evening, I was watching TV with some little girls, and they were talking about HIV/AIDS; they had never seen the situation before. Now when they saw the documentary about HIV/AIDS they were so shocked! "HIV/AIDS really affected these people!" They were frightened. I told them, "This is how they were, but even now that we have medication, one should still stay safe"—there was a gentleman who gave his story, he had HIV for about 30years but he is healthy. He said, "Even if I am still healthy, this disease has still affected me" and it is still evident that he is infected. There is a young adult who said he got HIV because he was promiscuous, and one girl said...<unintelligible>. So people really need to be aware and taught that technology actually exists. | | | | | | | | | | | | | | | |  |  |  |
|  |  |  |  |  |  |  |  |  |  |  |  |  |  |  |  |  |  |  |  |
|  |  |  |  |  |  |  |  |  |  |  |  |  |  |  |  |  |  |  |  |
|  | | | | | | | | | | | | | | | | | |  |  |
| Reports\\Coding Summary By Code Report | | | | | | | | | | Page 168 of 409 | | | | | | | |  |  |
| 02/03/2022 02:43 | | | | | | | | | | | | | | | | | |  |  |
|  | | | **Aggregate** |  | **Classification** |  | **Coverage** |  | **Number Of Coding References** | |  | **Reference Number** |  | **Coded By Initials** |  | **Modified On** |  |  |  |
|  | | | | | | | | | | | | | | | | | |  |  |
|  | | | | | | | | | | | | 13 |  | AT |  | 01/03/2022 09:09 |  |  |  |
|  | Indeed. Because—let me first start with the instance of the religious person who was talking the client. Because if you look at the bibles we read, they have editions; having editions means every generation has something different. And the bible says something--a certain leader used it and said, "You will have different children that will change the narrative of their community" you know this person; if I make the statement, it will sound political yet we are in research right now—  <laughs>  Do you understand? And the bible itself says, "we have to be as wise as serpents" it means our brains are always active and that is why we come up with different innovations. So we have different references to show those people that it is not all about the bible, even now as a human, you could be a moving bible because you are the one who puts the bible's instructions into practice. Because it would be in vain if you don’t put it into practice. So I personally think, some people don’t believe in science; some do and others don’t. An example; there are some churches I know that believe HIV is cured through the bible; they pray for it and it is cured. The more one goes to church, the more they deteriorate but when he/she goes home, "My pastor prays!" but the one telling him that he would get cured, is taking his pills. Do you get me? We also have some people who don’t wish others well in religion. So I don’t say it in bad faith, but I give the scenario; but ever since people discovered the truth—you find that one goes to church from Monday to Sunday, he has bible studies but knows that he has to take his medicine at 10pm in order to survive. So this person says, "I shall pray and plead the blood of Jesus, but continue taking my medicine" which means people understand science. You see even the elderly are on tiktok! How about us in research. So I think we should just continue to assure them and show them the advantages, benefits of technology because in this day and age, if an elderly person is able to hold a phone worth shs. 300,000 but another person says, "I don’t need it!" The old person even buys data and takes selfies; you understand? Which means we need to show people that every day that goes by, you cannot remain the same; every month and day that goes by—if you were 2years old—I started by crawling but now I can walk, run, speak, speak to those older than me and even those who have just been born. Which means there is always a new update, and technology is always improving...<unintelligible>Before my mother passed away, I was amazed and asked her, "Mother, don’t you think to yourself how you are in Uganda but you are able to communicate with your brother in America? How do you think things move on?" and she said, "We expected this; actually the clothes and shoes you wear now, are the ones we used to have in the days of the 'twist dance', the old trends like checked clothes, bell bottoms, are just coming back in this current generation".  So I think the more we sensitize people about the benefits, the more they will comprehend and adapt to it, then move on with it. | | | | | | | | | | | | | | | |  |  |  |
|  |  |  |  |  |  |  |  |  |  |  |  |  |  |  |  |  |  |  |  |
|  |  |  |  |  |  |  |  |  |  |  |  |  |  |  |  |  |  |  |  |
|  |  |  |  |  |  |  |  |  |  |  |  |  |  |  |  |  |  |  |  |
|  |  | | | | | | | | | | | | | | | |  |  |  |
|  | | | | | | | | | | | | 14 |  | AT |  | 01/03/2022 09:08 |  |  |  |
|  | Indeed. Because—let me first start with the instance of the religious person who was talking the client. Because if you look at the bibles we read, they have editions; having editions means every generation has something different. And the bible says something--a certain leader used it and said, "You will have different children that will change the narrative of their community" you know this person; if I make the statement, it will sound political yet we are in research right now—  <laughs>  Do you understand? And the bible itself says, "we have to be as wise as serpents" it means our brains are always active and that is why we come up with different innovations. So we have different references to show those people that it is not all about the bible, even now as a human, you could be a moving bible because you are the one who puts the bible's instructions into practice. Because it would be in vain if you don’t put it into practice. So I personally think, some people don’t believe in science; some do and others don’t. An example; there are some churches I know that believe HIV is cured through the bible; they pray for it and it is cured. The more one goes to church, the more they deteriorate but when he/she goes home, "My pastor prays!" but the one telling him that he would get cured, is taking his pills. Do you get me? We also have some people who don’t wish others well in religion. So I don’t say it in bad faith, but I give the scenario; but ever since people discovered the truth—you find that one goes to church from Monday to Sunday, he has bible studies but knows that he has to take his medicine at 10pm in order to survive. So this person says, "I shall pray and plead the blood of Jesus, but continue taking my medicine" which means people understand science. You see even the elderly are on tiktok! How about us in research. So I think we should just continue to assure them and show them the advantages, benefits of technology because in this day and age, if an elderly person is able to hold a phone worth shs. 300,000 but another person says, "I don’t need it!" The old person even buys data and takes selfies; you understand? Which means we need to show people that every day that goes by, you cannot remain the same; every month and day that goes by—if you were 2years old—I started by crawling but now I can walk, run, speak, speak to those older than me and even those who have just been born. Which means there is always a new update, and technology is always improving...<unintelligible>Before my mother passed away, I was amazed and asked her, "Mother, don’t you think to yourself how you are in Uganda but you are able to communicate with your brother in America? How do you think things move on?" and she said, "We expected this; actually the clothes and shoes you wear now, are the ones we used to have in the days of the 'twist dance', the old trends like checked clothes, bell bottoms, are just coming back in this current generation".  So I think the more we sensitize people about the benefits, the more they will comprehend and adapt to it, then move on with it. | | | | | | | | | | | | | | | |  |  |  |
|  |  |  |  |  |  |  |  |  |  |  |  |  |  |  |  |  |  |  |  |
|  |  |  |  |  |  |  |  |  |  |  |  |  |  |  |  |  |  |  |  |
|  |  |  |  |  |  |  |  |  |  |  |  |  |  |  |  |  |  |  |  |
|  |  | | | | | | | | | | | | | | | |  |  |  |
|  | | | | | | | | | | | | | | | | | |  |  |
|  | | | | | | | | | | | | | | | | | |  |  |
| Reports\\Coding Summary By Code Report | | | | | | | | | | Page 169 of 409 | | | | | | | |  |  |
| 02/03/2022 02:43 | | | | | | | | | | | | | | | | | |  |  |
|  | | | **Aggregate** |  | **Classification** |  | **Coverage** |  | **Number Of Coding References** | |  | **Reference Number** |  | **Coded By Initials** |  | **Modified On** |  |  |  |
|  | | | | | | | | | | | | | | | | | |  |  |
|  | | | | | | | | | | | | 15 |  | AT |  | 01/03/2022 09:11 |  |  |  |
|  | M: Yes, will the people accept the results from the technology, especially those that say, 'in the future, this is what will happen'?  P7: I think they will accept the technology; the answer is actually yes and no. Because if you look at the data in this country, majority of the population is young people and they are blessed to live in the Information technology era and they strongly believe in it. One thing I admire about the young people is, while we were still in school, we didn’t study the Entrepreneurship course. And it gave us hard time to know what 'business' is; and these children who have studied entrepreneurship are well versed in finance. While we were in school, we thought we just had to study and get jobs and then pay monthly bills but things have changed. The children who have studied the entrepreneurship course, they are versatile and change with the trends, and since it is majority of the population, they are well versed with science and prove the facts. Since they are the majority, those who do not want to adapt to the computer age will remain backward; we shall be just like the ones...<unintelligible> because we remained in the analog age yet the world is evolving.  We didn’t think a time would come where we are 50 people on a zoom meeting; but that is where the world is headed. In 5-10years, the entire world will run on technology, so since the younger generations have had that chance, they have grasped it faster. They will just go ahead and check, "What are the research results?" Take a look at the vaccinations for COVID, they just press this and the certificate number is given because we used to think the vaccination card was the certificate, which isn’t the case. So I believe they are in the technology age and in the future, if they experience any symptoms, they first make a diagnosis themselves using technology and then come to the doctor. And the doctor will have to also be an IT expert, not just a general doctor, so I think they will accept very well and continue with it. Another reason I say no is, for us who are rigid will just continue arguing and disagree, "It used to be like this". One time I was sent, "Go to Mpelerwe, our home is just next to the big tree" but the tree is no longer there; instead there are shops, etc, and yet this person is giving you directions based on what used to be there. So things have changed drastically, whether we like it or not; some of us will be left hanging, because the world is moving at a terrific speed. You just find yourself...<unintelligible> we used to pick up phone calls like this unlike now, but these days the young people are always on their phones, trying to keep up with the current trends. During our days—I wonder if the landline phones still exist; I think they are found in offices. So I think in the coming time, someone will just tab a button and all the data is presented.  P2: Now about these people's perception of science and technology; majority believe in science but others don’t believe in science. But the challenge of being rigid is—what makes them rigid when it comes to technology is, they put the blame on people, "We don’t trust the government, they are planning to do this" others believe conspiracy theories, "The whites just want to get rid of us and then take over our country". And take an example of the COVID vaccinations, "I don’t trust the first vaccine, I want Johnson and Johnson" Who manufactures it? It is for the whites; we were told". So don’t you see that there are information gaps, and they claim, "You are vaccinated so in a few years, all of you are going to die!"  ...<unintelligible> So you find that those are the challenges we have. Some of them still say, "We just check our children to know if they belong to our family" people don’t believe in that anymore. So if you don’t explain to them, some will believe in science and others won’t. But there are some who will never change because they still believe, "The owner of the bed and woman is the owner of the child" that is the law in Uganda! Until you prove beyond reasonable doubt that the child is not yours. But the child born in your house, is yours and nothing else! ...<Noise>  <unintelligible>  So you find that the court says this, the DNA says otherwise. So that is the challenge of science but our people have accepted science especially if you sensitize them. One may refuse to get vaccinated for COVID but thinks to oneself, "These children were vaccinated for the 9 child-killer diseases. Every child is immunized but we don’t know where those vaccines are manufactured" then eventually he comes back and says, "I think science is necessary! Let us just go ahead with everything". So people believe in science, some don’t—they are mixed in the community. But others are rigid and don’t believe in science. | | | | | | | | | | | | | | | |  |  |  |
|  |  |  |  |  |  |  |  |  |  |  |  |  |  |  |  |  |  |  |  |
|  |  |  |  |  |  |  |  |  |  |  |  |  |  |  |  |  |  |  |  |
|  |  |  |  |  |  |  |  |  |  |  |  |  |  |  |  |  |  |  |  |
|  |  |  |  |  |  |  |  |  |  |  |  |  |  |  |  |  |  |  |  |
|  |  |  |  |  |  |  |  |  |  |  |  |  |  |  |  |  |  |  |  |
|  |  | | | | | | | | | | | | | | | |  |  |  |
|  | | | | | | | | | | | | 16 |  | AT |  | 02/03/2022 01:23 |  |  |  |
|  | P2: Now about these people's perception of science and technology; majority believe in science but others don’t believe in science. But the challenge of being rigid is—what makes them rigid when it comes to technology is, they put the blame on people, "We don’t trust the government, they are planning to do this" others believe conspiracy theories, "The whites just want to get rid of us and then take over our country". And take an example of the COVID vaccinations, "I don’t trust the first vaccine, I want Johnson and Johnson" Who manufactures it? It is for the whites; we were told". So don’t you see that there are information gaps, and they claim, "You are vaccinated so in a few years, all of you are going to die!"  ...<unintelligible> So you find that those are the challenges we have. Some of them still say, "We just check our children to know if they belong to our family" people don’t believe in that anymore. So if you don’t explain to them, some will believe in science and others won’t. But there are some who will never change because they still believe, "The owner of the bed and woman is the owner of the child" that is the law in Uganda! Until you prove beyond reasonable doubt that the child is not yours. But the child born in your house, is yours and nothing else! ...<Noise> | | | | | | | | | | | | | | | |  |  |  |
|  |  |  |  |  |  |  |  |  |  |  |  |  |  |  |  |  |  |  |  |
|  |  | | | | | | | | | | | | | | | |  |  |  |
|  | | | | | | | | | | | | | | | | | |  |  |
|  | | | | | | | | | | | | | | | | | |  |  |
| Reports\\Coding Summary By Code Report | | | | | | | | | | Page 170 of 409 | | | | | | | |  |  |
| 02/03/2022 02:43 | | | | | | | | | | | | | | | | | |  |  |
|  | | | **Aggregate** |  | **Classification** |  | **Coverage** |  | **Number Of Coding References** | |  | **Reference Number** |  | **Coded By Initials** |  | **Modified On** |  |  |  |
|  | **Nodes\\Community engagement\Building trust** | | | | | | | | | | | | | | | |  |  |  |
|  | | **Document** | | | | | | | | | | | | | | |  |  |  |
|  | | | **Files\\FGD for CAB #1_1_Transcript** | | | | | | | | | | | | | |  |  |  |
|  |  |  | Yes |  |  |  | 0.0129 |  | 1 | |  | | | | | |  |  |  |
|  | | |  |  |  |  |  |  |  | |  | | | | | | |  |  |
|  | | | | | | | | | | | | 1 |  | AT |  | 01/03/2022 09:16 |  |  |  |
|  | P6: Releasing those results would be good by the end of the day, but then it also depends on a particular group of people you are going to give that information to. So you are supposed to sit with the health provider, and even the way you are going to break that information in a way that is going to be understandable and all that, things like that. Because we have particular groups of people in the clinic; the mothers, the MH people, people in studies and all that. So it’s just a way of finding how to bring it out well so that by the end of the day, that person can understand what came out of the study. | | | | | | | | | | | | | | | |  |  |  |
|  |  | | | | | | | | | | | | | | | |  |  |  |
|  | | | **Files\\FGD for CAB #3_3_Transcript** | | | | | | | | | | | | | |  |  |  |
|  |  |  | Yes |  |  |  | 0.0183 |  | 2 | |  | | | | | |  |  |  |
|  | | |  |  |  |  |  |  |  | |  | | | | | | |  |  |
|  | | | | | | | | | | | | 1 |  | AT |  | 28/02/2022 19:02 |  |  |  |
|  | P3: Yes, [moderator]. As CAB members I think we need to create a relationship and by creating a relationship, that helps you to prepare that person. When you are creating a relationship with this person, you are actually trying to create some attachment to this person. So it becomes very easy for you as you prepare, as you create a relationship and prepare this person, this person is going to gain confidence from you and finally you can break any news at some point in time—not breaking it abruptly, but creating that attachment is very key as a CAB member. | | | | | | | | | | | | | | | |  |  |  |
|  |  | | | | | | | | | | | | | | | |  |  |  |
|  | | | | | | | | | | | | 2 |  | AT |  | 28/02/2022 22:29 |  |  |  |
|  | because there is no such thing as 'one size fits all' [Hmm, hmm] it is dependent on the individuals. Because they'll tell you pointblank, "Ahh I don’t want to hear what that man is saying; he is even tough. If you try to ask him a question, he says he is in a hurry" so that person is already as blocked. So the messenger is very important. [Yeah the messenger] If the participant has built a relationship, a special relationship with the clinician, let the clinician give them that information. | | | | | | | | | | | | | | | |  |  |  |
|  |  | | | | | | | | | | | | | | | |  |  |  |
|  | | | **Files\\FGD for CAB #5_5_Transcript** | | | | | | | | | | | | | |  |  |  |
|  |  |  | Yes |  |  |  | 0.0112 |  | 1 | |  | | | | | |  |  |  |
|  | | |  |  |  |  |  |  |  | |  | | | | | | |  |  |
|  | | | | | | | | | | | | 1 |  | AT |  | 02/03/2022 01:15 |  |  |  |
|  | P6: Technology especially on the side of Muslims; they don’t believe in that because they are taught different doctrines that say, "If you refuse to believe the prophet, you are condemned". An example is, there are some people I advised to go for the paternity test. Sometime back when the Muslim leader was around, there is a lady who had a child and two men claimed paternity of the child. So during that time, they asked, "Who is this woman's husband?" and they said, "It is him" and "Who is the owner of the bed?" "It is him". So he said, "I want to let you know that the owner of this bed is the father of the child". Now that is how the judgement is made in Islam— | | | | | | | | | | | | | | | |  |  |  |
|  |  | | | | | | | | | | | | | | | |  |  |  |
|  | | | | | | | | | | | | | | | | | |  |  |
|  | | | | | | | | | | | | | | | | | |  |  |
| Reports\\Coding Summary By Code Report | | | | | | | | | | Page 171 of 409 | | | | | | | |  |  |
| 02/03/2022 02:43 | | | | | | | | | | | | | | | | | |  |  |
|  | | | **Aggregate** |  | **Classification** |  | **Coverage** |  | **Number Of Coding References** | |  | **Reference Number** |  | **Coded By Initials** |  | **Modified On** |  |  |  |
|  | **Nodes\\Community engagement\Building trust\Create a relationship with person as a CAB member to prepare to receive results** | | | | | | | | | | | | | | | |  |  |  |
|  | | **Document** | | | | | | | | | | | | | | |  |  |  |
|  | | | **Files\\FGD for CAB #3_3_Transcript** | | | | | | | | | | | | | |  |  |  |
|  |  |  | No |  |  |  | 0.0096 |  | 1 | |  | | | | | |  |  |  |
|  | | |  |  |  |  |  |  |  | |  | | | | | | |  |  |
|  | | | | | | | | | | | | 1 |  | AT |  | 28/02/2022 19:02 |  |  |  |
|  | P3: Yes, [moderator]. As CAB members I think we need to create a relationship and by creating a relationship, that helps you to prepare that person. When you are creating a relationship with this person, you are actually trying to create some attachment to this person. So it becomes very easy for you as you prepare, as you create a relationship and prepare this person, this person is going to gain confidence from you and finally you can break any news at some point in time—not breaking it abruptly, but creating that attachment is very key as a CAB member. | | | | | | | | | | | | | | | |  |  |  |
|  |  | | | | | | | | | | | | | | | |  |  |  |
|  | **Nodes\\Community engagement\Building trust\create rapport** | | | | | | | | | | | | | | | |  |  |  |
|  | | **Document** | | | | | | | | | | | | | | |  |  |  |
|  | | | **Files\\FGD for CAB #1_1_Transcript** | | | | | | | | | | | | | |  |  |  |
|  |  |  | No |  |  |  | 0.0129 |  | 1 | |  | | | | | |  |  |  |
|  | | |  |  |  |  |  |  |  | |  | | | | | | |  |  |
|  | | | | | | | | | | | | 1 |  | AT |  | 01/03/2022 09:16 |  |  |  |
|  | P6: Releasing those results would be good by the end of the day, but then it also depends on a particular group of people you are going to give that information to. So you are supposed to sit with the health provider, and even the way you are going to break that information in a way that is going to be understandable and all that, things like that. Because we have particular groups of people in the clinic; the mothers, the MH people, people in studies and all that. So it’s just a way of finding how to bring it out well so that by the end of the day, that person can understand what came out of the study. | | | | | | | | | | | | | | | |  |  |  |
|  |  | | | | | | | | | | | | | | | |  |  |  |
|  | | | | | | | | | | | | | | | | | |  |  |
|  | | | | | | | | | | | | | | | | | |  |  |
|  | | | | | | | | | | | | | | | | | |  |  |
|  | | | | | | | | | | | | | | | | | |  |  |
| Reports\\Coding Summary By Code Report | | | | | | | | | | Page 172 of 409 | | | | | | | |  |  |
| 02/03/2022 02:43 | | | | | | | | | | | | | | | | | |  |  |
|  | | | **Aggregate** |  | **Classification** |  | **Coverage** |  | **Number Of Coding References** | |  | **Reference Number** |  | **Coded By Initials** |  | **Modified On** |  |  |  |
|  | **Nodes\\Community engagement\Building trust\Embracing and acceptability of emerging technologies** | | | | | | | | | | | | | | | |  |  |  |
|  | | **Document** | | | | | | | | | | | | | | |  |  |  |
|  | | | **Files\\FGD for CAB #5_5_Transcript** | | | | | | | | | | | | | |  |  |  |
|  |  |  | No |  |  |  | 0.0112 |  | 1 | |  | | | | | |  |  |  |
|  | | |  |  |  |  |  |  |  | |  | | | | | | |  |  |
|  | | | | | | | | | | | | 1 |  | AT |  | 02/03/2022 01:15 |  |  |  |
|  | P6: Technology especially on the side of Muslims; they don’t believe in that because they are taught different doctrines that say, "If you refuse to believe the prophet, you are condemned". An example is, there are some people I advised to go for the paternity test. Sometime back when the Muslim leader was around, there is a lady who had a child and two men claimed paternity of the child. So during that time, they asked, "Who is this woman's husband?" and they said, "It is him" and "Who is the owner of the bed?" "It is him". So he said, "I want to let you know that the owner of this bed is the father of the child". Now that is how the judgement is made in Islam— | | | | | | | | | | | | | | | |  |  |  |
|  |  | | | | | | | | | | | | | | | |  |  |  |
|  | **Nodes\\Community engagement\Building trust\politeness** | | | | | | | | | | | | | | | |  |  |  |
|  | | **Document** | | | | | | | | | | | | | | |  |  |  |
|  | | | **Files\\FGD for CAB #3_3_Transcript** | | | | | | | | | | | | | |  |  |  |
|  |  |  | No |  |  |  | 0.0086 |  | 1 | |  | | | | | |  |  |  |
|  | | |  |  |  |  |  |  |  | |  | | | | | | |  |  |
|  | | | | | | | | | | | | 1 |  | AT |  | 28/02/2022 22:29 |  |  |  |
|  | because there is no such thing as 'one size fits all' [Hmm, hmm] it is dependent on the individuals. Because they'll tell you pointblank, "Ahh I don’t want to hear what that man is saying; he is even tough. If you try to ask him a question, he says he is in a hurry" so that person is already as blocked. So the messenger is very important. [Yeah the messenger] If the participant has built a relationship, a special relationship with the clinician, let the clinician give them that information. | | | | | | | | | | | | | | | |  |  |  |
|  |  | | | | | | | | | | | | | | | |  |  |  |
|  | **Nodes\\Community engagement\factors hindering emerging technologies** | | | | | | | | | | | | | | | |  |  |  |
|  | | **Document** | | | | | | | | | | | | | | |  |  |  |
|  | | | **Files\\FGD for CAB #2_2_Transcript** | | | | | | | | | | | | | |  |  |  |
|  |  |  | Yes |  |  |  | 0.0119 |  | 1 | |  | | | | | |  |  |  |
|  | | |  |  |  |  |  |  |  | |  | | | | | | |  |  |
|  | | | | | | | | | | | | 1 |  | AT |  | 02/03/2022 01:31 |  |  |  |
|  | P6: About the issue of releasing results like you have said; the truth is getting advice from someone especially if they are not a professional in that field, is very dangerous. Because most people have myths, so many facts they do not know about; you find that one says 'HIV kills' but does not know how to handle the disease. I believe it is dangerous to seek advice from people and there are so many people that do this; they advised each other and said whoever suffered from HIV was bewitched. It wasn’t the HIV+ patient that says this, but the people in the community that they sought advice from, are the ones that gave the wrong information. Those people exist, those who pose risks in regard to that issue. Thank you very much. | | | | | | | | | | | | | | | |  |  |  |
|  |  | | | | | | | | | | | | | | | |  |  |  |
| Reports\\Coding Summary By Code Report | | | | | | | | | | Page 173 of 409 | | | | | | | |  |  |
| 02/03/2022 02:43 | | | | | | | | | | | | | | | | | |  |  |
|  | | | **Aggregate** |  | **Classification** |  | **Coverage** |  | **Number Of Coding References** | |  | **Reference Number** |  | **Coded By Initials** |  | **Modified On** |  |  |  |
|  | | | **Files\\FGD for CAB #5_5_Transcript** | | | | | | | | | | | | | |  |  |  |
|  |  |  | Yes |  |  |  | 0.0217 |  | 1 | |  | | | | | |  |  |  |
|  | | |  |  |  |  |  |  |  | |  | | | | | | |  |  |
|  | | | | | | | | | | | | 1 |  | AT |  | 02/03/2022 01:23 |  |  |  |
|  | P2: Now about these people's perception of science and technology; majority believe in science but others don’t believe in science. But the challenge of being rigid is—what makes them rigid when it comes to technology is, they put the blame on people, "We don’t trust the government, they are planning to do this" others believe conspiracy theories, "The whites just want to get rid of us and then take over our country". And take an example of the COVID vaccinations, "I don’t trust the first vaccine, I want Johnson and Johnson" Who manufactures it? It is for the whites; we were told". So don’t you see that there are information gaps, and they claim, "You are vaccinated so in a few years, all of you are going to die!"  ...<unintelligible> So you find that those are the challenges we have. Some of them still say, "We just check our children to know if they belong to our family" people don’t believe in that anymore. So if you don’t explain to them, some will believe in science and others won’t. But there are some who will never change because they still believe, "The owner of the bed and woman is the owner of the child" that is the law in Uganda! Until you prove beyond reasonable doubt that the child is not yours. But the child born in your house, is yours and nothing else! ...<Noise> | | | | | | | | | | | | | | | |  |  |  |
|  |  |  |  |  |  |  |  |  |  |  |  |  |  |  |  |  |  |  |  |
|  |  | | | | | | | | | | | | | | | |  |  |  |
|  | **Nodes\\Community engagement\factors hindering emerging technologies\Community myths and conspiracy theories** | | | | | | | | | | | | | | | |  |  |  |
|  | | **Document** | | | | | | | | | | | | | | |  |  |  |
|  | | | **Files\\FGD for CAB #2_2_Transcript** | | | | | | | | | | | | | |  |  |  |
|  |  |  | No |  |  |  | 0.0119 |  | 1 | |  | | | | | |  |  |  |
|  | | |  |  |  |  |  |  |  | |  | | | | | | |  |  |
|  | | | | | | | | | | | | 1 |  | AT |  | 02/03/2022 01:31 |  |  |  |
|  | P6: About the issue of releasing results like you have said; the truth is getting advice from someone especially if they are not a professional in that field, is very dangerous. Because most people have myths, so many facts they do not know about; you find that one says 'HIV kills' but does not know how to handle the disease. I believe it is dangerous to seek advice from people and there are so many people that do this; they advised each other and said whoever suffered from HIV was bewitched. It wasn’t the HIV+ patient that says this, but the people in the community that they sought advice from, are the ones that gave the wrong information. Those people exist, those who pose risks in regard to that issue. Thank you very much. | | | | | | | | | | | | | | | |  |  |  |
|  |  | | | | | | | | | | | | | | | |  |  |  |
|  | | | | | | | | | | | | | | | | | |  |  |
|  | | | | | | | | | | | | | | | | | |  |  |
|  | | | | | | | | | | | | | | | | | |  |  |
| Reports\\Coding Summary By Code Report | | | | | | | | | | Page 174 of 409 | | | | | | | |  |  |
| 02/03/2022 02:43 | | | | | | | | | | | | | | | | | |  |  |
|  | | | **Aggregate** |  | **Classification** |  | **Coverage** |  | **Number Of Coding References** | |  | **Reference Number** |  | **Coded By Initials** |  | **Modified On** |  |  |  |
|  | **Nodes\\Community engagement\factors hindering emerging technologies\Distrust in health care systems especially the government ones** | | | | | | | | | | | | | | | |  |  |  |
|  | | **Document** | | | | | | | | | | | | | | |  |  |  |
|  | | | **Files\\FGD for CAB #5_5_Transcript** | | | | | | | | | | | | | |  |  |  |
|  |  |  | No |  |  |  | 0.0217 |  | 1 | |  | | | | | |  |  |  |
|  | | |  |  |  |  |  |  |  | |  | | | | | | |  |  |
|  | | | | | | | | | | | | 1 |  | AT |  | 02/03/2022 01:23 |  |  |  |
|  | P2: Now about these people's perception of science and technology; majority believe in science but others don’t believe in science. But the challenge of being rigid is—what makes them rigid when it comes to technology is, they put the blame on people, "We don’t trust the government, they are planning to do this" others believe conspiracy theories, "The whites just want to get rid of us and then take over our country". And take an example of the COVID vaccinations, "I don’t trust the first vaccine, I want Johnson and Johnson" Who manufactures it? It is for the whites; we were told". So don’t you see that there are information gaps, and they claim, "You are vaccinated so in a few years, all of you are going to die!"  ...<unintelligible> So you find that those are the challenges we have. Some of them still say, "We just check our children to know if they belong to our family" people don’t believe in that anymore. So if you don’t explain to them, some will believe in science and others won’t. But there are some who will never change because they still believe, "The owner of the bed and woman is the owner of the child" that is the law in Uganda! Until you prove beyond reasonable doubt that the child is not yours. But the child born in your house, is yours and nothing else! ...<Noise> | | | | | | | | | | | | | | | |  |  |  |
|  |  |  |  |  |  |  |  |  |  |  |  |  |  |  |  |  |  |  |  |
|  |  | | | | | | | | | | | | | | | |  |  |  |
|  | **Nodes\\Community engagement\Importance of involving committee** | | | | | | | | | | | | | | | |  |  |  |
|  | | **Document** | | | | | | | | | | | | | | |  |  |  |
|  | | | **Files\\FGD for CAB #3_3_Transcript** | | | | | | | | | | | | | |  |  |  |
|  |  |  | Yes |  |  |  | 0.0050 |  | 1 | |  | | | | | |  |  |  |
|  | | |  |  |  |  |  |  |  | |  | | | | | | |  |  |
|  | | | | | | | | | | | | 1 |  | AT |  | 28/02/2022 19:12 |  |  |  |
|  | So for us as CAB members, our role is to share much—as accurate as information at community level, and so that the participants who are coming to the clinic already have a glimpse—an accurate glimpse of what we expect. And then the counsellor does the job uh—of finalizing it. Over to you. | | | | | | | | | | | | | | | |  |  |  |
|  |  | | | | | | | | | | | | | | | |  |  |  |
|  | | | **Files\\FGD for CAB #5_5_Transcript** | | | | | | | | | | | | | |  |  |  |
|  |  |  | Yes |  |  |  | 0.0444 |  | 9 | |  | | | | | |  |  |  |
|  | | |  |  |  |  |  |  |  | |  | | | | | | |  |  |
|  | | | | | | | | | | | | 1 |  | AT |  | 01/03/2022 08:09 |  |  |  |
|  | P2: I think this committee is necessary because it is all about research; let them first do trials and research, 'there could be some challenges, what happens when the harm is done? Is it easy?' That research should first be done before— | | | | | | | | | | | | | | | |  |  |  |
|  |  | | | | | | | | | | | | | | | |  |  |  |
|  | | | | | | | | | | | | 2 |  | AT |  | 01/03/2022 08:11 |  |  |  |
|  | P4: I think every research study has committees. And before research is done in a community, there are committees that first sit and discuss about it. If there is anything they think is harmful, it is prohibited from going into the community. Therefore, I would say if the committee suspects it—because...<unintelligible>but I it wouldn’t happen if there was another committee. | | | | | | | | | | | | | | | |  |  |  |
| Reports\\Coding Summary By Code Report | | | | | | | | | | Page 176 of 409 | | | | | | | |  |  |
| 02/03/2022 02:43 | | | | | | | | | | | | | | | | | |  |  |
|  | | | **Aggregate** |  | **Classification** |  | **Coverage** |  | **Number Of Coding References** | |  | **Reference Number** |  | **Coded By Initials** |  | **Modified On** |  |  |  |
|  | **Nodes\\Community engagement\Importance of involving committee\CAB members role accurate information at community level** | | | | | | | | | | | | | | | |  |  |  |
|  | | **Document** | | | | | | | | | | | | | | |  |  |  |
|  | | | **Files\\FGD for CAB #3_3_Transcript** | | | | | | | | | | | | | |  |  |  |
|  |  |  | No |  |  |  | 0.0050 |  | 1 | |  | | | | | |  |  |  |
|  | | |  |  |  |  |  |  |  | |  | | | | | | |  |  |
|  | | | | | | | | | | | | 1 |  | AT |  | 28/02/2022 19:12 |  |  |  |
|  | So for us as CAB members, our role is to share much—as accurate as information at community level, and so that the participants who are coming to the clinic already have a glimpse—an accurate glimpse of what we expect. And then the counsellor does the job uh—of finalizing it. Over to you. | | | | | | | | | | | | | | | |  |  |  |
|  |  | | | | | | | | | | | | | | | |  |  |  |
|  | **Nodes\\Community engagement\Importance of involving committee\Committee can separate difficult results from good ones.** | | | | | | | | | | | | | | | |  |  |  |
|  | | **Document** | | | | | | | | | | | | | | |  |  |  |
|  | | | **Files\\FGD for CAB #5_5_Transcript** | | | | | | | | | | | | | |  |  |  |
|  |  |  | No |  |  |  | 0.0072 |  | 1 | |  | | | | | |  |  |  |
|  | | |  |  |  |  |  |  |  | |  | | | | | | |  |  |
|  | | | | | | | | | | | | 1 |  | AT |  | 02/03/2022 01:11 |  |  |  |
|  | P1: The committee should be put in place because it is going to separate the difficult results and the good results to return to the research participants. And after scrutinizing them, they will find a way forward on how to return these results. I suggest that after discussing how difficult the results are, they should get someone or counselors they could train on how to tackle those issues and disclose to those participants. | | | | | | | | | | | | | | | |  |  |  |
|  |  | | | | | | | | | | | | | | | |  |  |  |
|  | | | | | | | | | | | | | | | | | |  |  |
|  | | | | | | | | | | | | | | | | | |  |  |
|  | | | | | | | | | | | | | | | | | |  |  |
|  | | | | | | | | | | | | | | | | | |  |  |
| Reports\\Coding Summary By Code Report | | | | | | | | | | Page 177 of 409 | | | | | | | |  |  |
| 02/03/2022 02:43 | | | | | | | | | | | | | | | | | |  |  |
|  | | | **Aggregate** |  | **Classification** |  | **Coverage** |  | **Number Of Coding References** | |  | **Reference Number** |  | **Coded By Initials** |  | **Modified On** |  |  |  |
|  | **Nodes\\Community engagement\Importance of involving committee\Committee could research on how best ROR can be done** | | | | | | | | | | | | | | | |  |  |  |
|  | | **Document** | | | | | | | | | | | | | | |  |  |  |
|  | | | **Files\\FGD for CAB #5_5_Transcript** | | | | | | | | | | | | | |  |  |  |
|  |  |  | No |  |  |  | 0.0155 |  | 1 | |  | | | | | |  |  |  |
|  | | |  |  |  |  |  |  |  | |  | | | | | | |  |  |
|  | | | | | | | | | | | | 1 |  | AT |  | 01/03/2022 08:22 |  |  |  |
|  | P2: The issue of the committee and counselors has come up. I would personally say, take an example of HIV/AIDS; after realizing the impact of the results, you find that the one performing the test and counselor are different people. The one telling you to get the test, and the one testing the blood sample, are different people all together. It is the trained counselor who gives you the results whether you are positive or not. So that setting was put in place by the committee after researching on how best it can be done. So according to the committee, the best person to deliver the results is the counselor who is able to do the counselling. So after the committee, they decide that the counselor should be engaged in the delivery of these results; you will find that the researcher and doctor should not deliver the results to the participant. The counselor should take the results because they are trained at counseling. | | | | | | | | | | | | | | | |  |  |  |
|  |  |  |  |  |  |  |  |  |  |  |  |  |  |  |  |  |  |  |  |
|  |  | | | | | | | | | | | | | | | |  |  |  |
|  | **Nodes\\Community engagement\Importance of involving committee\Committee discuss how difficult the results are** | | | | | | | | | | | | | | | |  |  |  |
|  | | **Document** | | | | | | | | | | | | | | |  |  |  |
|  | | | **Files\\FGD for CAB #5_5_Transcript** | | | | | | | | | | | | | |  |  |  |
|  |  |  | No |  |  |  | 0.0072 |  | 1 | |  | | | | | |  |  |  |
|  | | |  |  |  |  |  |  |  | |  | | | | | | |  |  |
|  | | | | | | | | | | | | 1 |  | AT |  | 01/03/2022 08:25 |  |  |  |
|  | P1: The committee should be put in place because it is going to separate the difficult results and the good results to return to the research participants. And after scrutinizing them, they will find a way forward on how to return these results. I suggest that after discussing how difficult the results are, they should get someone or counselors they could train on how to tackle those issues and disclose to those participants. | | | | | | | | | | | | | | | |  |  |  |
|  |  | | | | | | | | | | | | | | | |  |  |  |
|  | | | | | | | | | | | | | | | | | |  |  |
|  | | | | | | | | | | | | | | | | | |  |  |
|  | | | | | | | | | | | | | | | | | |  |  |
| Reports\\Coding Summary By Code Report | | | | | | | | | | Page 178 of 409 | | | | | | | |  |  |
| 02/03/2022 02:43 | | | | | | | | | | | | | | | | | |  |  |
|  | | | **Aggregate** |  | **Classification** |  | **Coverage** |  | **Number Of Coding References** | |  | **Reference Number** |  | **Coded By Initials** |  | **Modified On** |  |  |  |
|  | **Nodes\\Community engagement\Importance of involving committee\Committee scrutinizes results and finds a way forward on how to return these results.** | | | | | | | | | | | | | | | |  |  |  |
|  | | **Document** | | | | | | | | | | | | | | |  |  |  |
|  | | | **Files\\FGD for CAB #5_5_Transcript** | | | | | | | | | | | | | |  |  |  |
|  |  |  | No |  |  |  | 0.0072 |  | 1 | |  | | | | | |  |  |  |
|  | | |  |  |  |  |  |  |  | |  | | | | | | |  |  |
|  | | | | | | | | | | | | 1 |  | AT |  | 01/03/2022 08:25 |  |  |  |
|  | P1: The committee should be put in place because it is going to separate the difficult results and the good results to return to the research participants. And after scrutinizing them, they will find a way forward on how to return these results. I suggest that after discussing how difficult the results are, they should get someone or counselors they could train on how to tackle those issues and disclose to those participants. | | | | | | | | | | | | | | | |  |  |  |
|  |  | | | | | | | | | | | | | | | |  |  |  |
|  | **Nodes\\Community engagement\Importance of involving committee\Committee should be in place to gauge the categories of results to be returned** | | | | | | | | | | | | | | | |  |  |  |
|  | | **Document** | | | | | | | | | | | | | | |  |  |  |
|  | | | **Files\\FGD for CAB #5_5_Transcript** | | | | | | | | | | | | | |  |  |  |
|  |  |  | No |  |  |  | 0.0041 |  | 1 | |  | | | | | |  |  |  |
|  | | |  |  |  |  |  |  |  | |  | | | | | | |  |  |
|  | | | | | | | | | | | | 1 |  | AT |  | 01/03/2022 08:24 |  |  |  |
|  | P1: The committee should be in place to gauge the categories of these results, when it comes to returning these results, they should have someone who will help to counsel this person so that the news does not affect him/her so much. That is all. | | | | | | | | | | | | | | | |  |  |  |
|  |  | | | | | | | | | | | | | | | |  |  |  |
|  | **Nodes\\Community engagement\Importance of involving committee\Has a role to play in Research** | | | | | | | | | | | | | | | |  |  |  |
|  | | **Document** | | | | | | | | | | | | | | |  |  |  |
|  | | | **Files\\FGD for CAB #5_5_Transcript** | | | | | | | | | | | | | |  |  |  |
|  |  |  | No |  |  |  | 0.0039 |  | 1 | |  | | | | | |  |  |  |
|  | | |  |  |  |  |  |  |  | |  | | | | | | |  |  |
|  | | | | | | | | | | | | 1 |  | AT |  | 01/03/2022 08:09 |  |  |  |
|  | P2: I think this committee is necessary because it is all about research; let them first do trials and research, 'there could be some challenges, what happens when the harm is done? Is it easy?' That research should first be done before— | | | | | | | | | | | | | | | |  |  |  |
|  |  | | | | | | | | | | | | | | | |  |  |  |
|  | | | | | | | | | | | | | | | | | |  |  |
| Reports\\Coding Summary By Code Report | | | | | | | | | | Page 179 of 409 | | | | | | | |  |  |
| 02/03/2022 02:43 | | | | | | | | | | | | | | | | | |  |  |
|  | | | **Aggregate** |  | **Classification** |  | **Coverage** |  | **Number Of Coding References** | |  | **Reference Number** |  | **Coded By Initials** |  | **Modified On** |  |  |  |
|  | **Nodes\\Community engagement\Importance of involving committee\Prohibit harmful research** | | | | | | | | | | | | | | | |  |  |  |
|  | | **Document** | | | | | | | | | | | | | | |  |  |  |
|  | | | **Files\\FGD for CAB #5_5_Transcript** | | | | | | | | | | | | | |  |  |  |
|  |  |  | No |  |  |  | 0.0062 |  | 1 | |  | | | | | |  |  |  |
|  | | |  |  |  |  |  |  |  | |  | | | | | | |  |  |
|  | | | | | | | | | | | | 1 |  | AT |  | 01/03/2022 08:11 |  |  |  |
|  | P4: I think every research study has committees. And before research is done in a community, there are committees that first sit and discuss about it. If there is anything they think is harmful, it is prohibited from going into the community. Therefore, I would say if the committee suspects it—because...<unintelligible>but I it wouldn’t happen if there was another committee. | | | | | | | | | | | | | | | |  |  |  |
|  |  | | | | | | | | | | | | | | | |  |  |  |
|  | **Nodes\\Community engagement\Importance of involving committee\Resolve disputes due to rumors from neighbors about results** | | | | | | | | | | | | | | | |  |  |  |
|  | | **Document** | | | | | | | | | | | | | | |  |  |  |
|  | | | **Files\\FGD for CAB #5_5_Transcript** | | | | | | | | | | | | | |  |  |  |
|  |  |  | No |  |  |  | 0.0073 |  | 1 | |  | | | | | |  |  |  |
|  | | |  |  |  |  |  |  |  | |  | | | | | | |  |  |
|  | | | | | | | | | | | | 1 |  | AT |  | 02/03/2022 01:10 |  |  |  |
|  | So I would suggest that there should be a committee because the words we are speaking and sharing--but right now in the area of Katanga or Mpelerwe or Mulago, there is a family that is having disputes due to rumors from neighbors—  M: Information that is not authentic;  P5: It is not proven nor is it authentic, you understand? So when it comes to research, that committee should be in place to see what could cause chaos and what cannot. | | | | | | | | | | | | | | | |  |  |  |
|  |  | | | | | | | | | | | | | | | |  |  |  |
|  | | | | | | | | | | | | | | | | | |  |  |
|  | | | | | | | | | | | | | | | | | |  |  |
|  | | | | | | | | | | | | | | | | | |  |  |
|  | | | | | | | | | | | | | | | | | |  |  |
| Reports\\Coding Summary By Code Report | | | | | | | | | | Page 180 of 409 | | | | | | | |  |  |
| 02/03/2022 02:43 | | | | | | | | | | | | | | | | | |  |  |
|  | | | **Aggregate** |  | **Classification** |  | **Coverage** |  | **Number Of Coding References** | |  | **Reference Number** |  | **Coded By Initials** |  | **Modified On** |  |  |  |
|  | **Nodes\\Community engagement\Importance of involving committee\See what cause chaos and what could cannot.** | | | | | | | | | | | | | | | |  |  |  |
|  | | **Document** | | | | | | | | | | | | | | |  |  |  |
|  | | | **Files\\FGD for CAB #5_5_Transcript** | | | | | | | | | | | | | |  |  |  |
|  |  |  | No |  |  |  | 0.0073 |  | 1 | |  | | | | | |  |  |  |
|  | | |  |  |  |  |  |  |  | |  | | | | | | |  |  |
|  | | | | | | | | | | | | 1 |  | AT |  | 02/03/2022 01:10 |  |  |  |
|  | So I would suggest that there should be a committee because the words we are speaking and sharing--but right now in the area of Katanga or Mpelerwe or Mulago, there is a family that is having disputes due to rumors from neighbors—  M: Information that is not authentic;  P5: It is not proven nor is it authentic, you understand? So when it comes to research, that committee should be in place to see what could cause chaos and what cannot. | | | | | | | | | | | | | | | |  |  |  |
|  |  | | | | | | | | | | | | | | | |  |  |  |
|  | **Nodes\\Community engagement\Role of CE in understanding PG results** | | | | | | | | | | | | | | | |  |  |  |
|  | | **Document** | | | | | | | | | | | | | | |  |  |  |
|  | | | **Files\\FGD for CAB #2_2_Transcript** | | | | | | | | | | | | | |  |  |  |
|  |  |  | Yes |  |  |  | 0.0295 |  | 1 | |  | | | | | |  |  |  |
|  | | |  |  |  |  |  |  |  | |  | | | | | | |  |  |
|  | | | | | | | | | | | | 1 |  | AT |  | 02/03/2022 01:34 |  |  |  |
|  | P6: Thank you very much. About consent of when to receive the results, is in 2 categories. Because the first example I would give is a child that is born with HIV; for instance, research is being done and the child is found HIV+, I believe the child is not given the results if he is 5years old. He just starts the treatment and after some time, the results are disclosed to him—but after he has already begun treatment. That means the consent; yes, he could consent but since he was not prepared—because you could disclose the results and he commits suicide at home. So it means the preparation for the risks involved; for today you could say 'Yes, you have given consent' but there are some results that you don’t give right away. Secondly, one is sick in the ward, one came after performing various tests and no clear diagnosis has been made; they reach a point and say, "There is research going on, maybe what we are doing could be of help". I will give you an example of meningitis; I was in ward 4C taking care of my friend who was participating in a certain research study, but they did not know what to do; the only possibility was to get the lumbar puncture, and the consent is supposed to be given. But the patient is able to give his consent, however, those around him are the ones who act on his behalf. What does that mean? That either you wait to disclose the results, so that the research participant himself first stabilizes and then you give him the results. Or else, the you disclose the results to the caretakers of the participant, then when he stabilizes, we disclose to him and then help him. But I think the results should be returned when the person is already in the research study. But examining him and then disclosing the results right away, sometimes he is not prepared to receive them. | | | | | | | | | | | | | | | |  |  |  |
|  |  |  |  |  |  |  |  |  |  |  |  |  |  |  |  |  |  |  |  |
|  |  | | | | | | | | | | | | | | | |  |  |  |
|  | | | | | | | | | | | | | | | | | |  |  |
|  | | | | | | | | | | | | | | | | | |  |  |
| Reports\\Coding Summary By Code Report | | | | | | | | | | Page 181 of 409 | | | | | | | |  |  |
| 02/03/2022 02:43 | | | | | | | | | | | | | | | | | |  |  |
|  | | | **Aggregate** |  | **Classification** |  | **Coverage** |  | **Number Of Coding References** | |  | **Reference Number** |  | **Coded By Initials** |  | **Modified On** |  |  |  |
|  | | | **Files\\FGD for CAB #3_3_Transcript** | | | | | | | | | | | | | |  |  |  |
|  |  |  | Yes |  |  |  | 0.0083 |  | 1 | |  | | | | | |  |  |  |
|  | | |  |  |  |  |  |  |  | |  | | | | | | |  |  |
|  | | | | | | | | | | | | 1 |  | AT |  | 02/03/2022 01:36 |  |  |  |
|  | P1: For—for—you see for us as CAB members, we shall speak out these things in our community dialogues, in our uh—in our preparatory meetings, in all those interventions. But at the study site—and I come to the point of how prepared are the—how should we prepare the participant. For me it takes me up to—I'll tell you that the quality of the preparation of the participant depends on the quality of the counsellors that you have in the study site. Because you should get the best. | | | | | | | | | | | | | | | |  |  |  |
|  |  | | | | | | | | | | | | | | | |  |  |  |
|  | **Nodes\\Community engagement\Role of CE in understanding PG results\speak out about ROR in community dialogues** | | | | | | | | | | | | | | | |  |  |  |
|  | | **Document** | | | | | | | | | | | | | | |  |  |  |
|  | | | **Files\\FGD for CAB #3_3_Transcript** | | | | | | | | | | | | | |  |  |  |
|  |  |  | No |  |  |  | 0.0083 |  | 1 | |  | | | | | |  |  |  |
|  | | |  |  |  |  |  |  |  | |  | | | | | | |  |  |
|  | | | | | | | | | | | | 1 |  | AT |  | 02/03/2022 01:36 |  |  |  |
|  | P1: For—for—you see for us as CAB members, we shall speak out these things in our community dialogues, in our uh—in our preparatory meetings, in all those interventions. But at the study site—and I come to the point of how prepared are the—how should we prepare the participant. For me it takes me up to—I'll tell you that the quality of the preparation of the participant depends on the quality of the counsellors that you have in the study site. Because you should get the best. | | | | | | | | | | | | | | | |  |  |  |
|  | **Nodes\\Community engagement\Role of CE in understanding PG results\speak out about ROR in community dialogues**  **Files\\FGD for CAB #1_1 Transcript** | | | | | | | | | | | | | | | |  |  |  |
| P1: If you want participants and families to own and utilize these results, you may need to identify a key person in that family, someone that is respected and can be listened to by most family members…. If one of their own can describe a given condition, while citing familiar stories, it will be easy for other family members to appreciate the results and own them… | | | | | | | | | | | | | | | | | |  | **Files\\FGD for CAB #3_3_Transcript** |
| Reports\\Coding Summary By Code Report | | | | | | | | | | Page 182 of 409 | | | | | | | |  |  |
| 02/03/2022 02:43 | | | | | | | | | | | | | | | | | |  |  |
|  | | | **Aggregate** |  | **Classification** |  | **Coverage** |  | **Number Of Coding References** | |  | **Reference Number** |  | **Coded By Initials** |  | **Modified On** |  |  |  |
|  | **Nodes\\Community engagement\Role of CE in understanding PG results\Teaching with examples** | | | | | | | | | | | | | | | |  |  |  |
|  | | **Document** | | | | | | | | | | | | | | |  |  |  |
|  | | | **Files\\FGD for CAB #2_2_Transcript** | | | | | | | | | | | | | |  |  |  |
|  |  |  | No |  |  |  | 0.0295 |  | 1 | |  | | | | | |  |  |  |
|  | | |  |  |  |  |  |  |  | |  | | | | | | |  |  |
|  | | | | | | | | | | | | 1 |  | AT |  | 02/03/2022 01:34 |  |  |  |
|  | P6: Thank you very much. About consent of when to receive the results, is in 2 categories. Because the first example I would give is a child that is born with HIV; for instance, research is being done and the child is found HIV+, I believe the child is not given the results if he is 5years old. He just starts the treatment and after some time, the results are disclosed to him—but after he has already begun treatment. That means the consent; yes, he could consent but since he was not prepared—because you could disclose the results and he commits suicide at home. So it means the preparation for the risks involved; for today you could say 'Yes, you have given consent' but there are some results that you don’t give right away. Secondly, one is sick in the ward, one came after performing various tests and no clear diagnosis has been made; they reach a point and say, "There is research going on, maybe what we are doing could be of help". I will give you an example of meningitis; I was in ward 4C taking care of my friend who was participating in a certain research study, but they did not know what to do; the only possibility was to get the lumbar puncture, and the consent is supposed to be given. But the patient is able to give his consent, however, those around him are the ones who act on his behalf. What does that mean? That either you wait to disclose the results, so that the research participant himself first stabilizes and then you give him the results. Or else, the you disclose the results to the caretakers of the participant, then when he stabilizes, we disclose to him and then help him. But I think the results should be returned when the person is already in the research study. But examining him and then disclosing the results right away, sometimes he is not prepared to receive them.   \| **Nodes\\Community engagement\Role of CE in understanding PG results\communicating in simple language** \| \| \| \| --- \| --- \| --- \| \| **Document** \| \| \| **Files\\FGD for CAB #5_5_Transcript** \|   P5: For me I am always concerned about the kind of language participants use when discussing genetics in communities. These this are too hard to understand… The language researchers use to communicate this information matters. Researchers should consider translating this information into local languages and consult community representatives on the appropriate local words to be used when explaining genomic terms. | | | | | | | | | | | | | | | |  |  |  |
|  |  |  |  |  |  |  |  |  |  |  |  |  |  |  |  |  |  |  |  |
|  | **Files\\FGD for CAB #1_1_Transcript**  P4: There are many languages in Uganda andsSometimes deciding on what language to use when speaking to a group of people is very hard. In Kampala, people speak more than three common languages so the researchers may even get confused on the most suitable language to choose over others | | | | | | | | | | | | | | | |  |  |  |
|  | | | | | | | | | | | | | | | | | |  |  |
|  | | | | | | | | | | | | | | | | | |  |  |
|  | | | | | | | | | | | | | | | | | |  |  |
|  | | | | | | | | | | | | | | | | | |  |  |
|  | | | | | | | | | | | | | | | | | |  |  |
|  | | | | | | | | | | | | | | | | | |  |  |
| Reports\\Coding Summary By Code Report | | | | | | | | | | Page 183 of 409 | | | | | | | |  |  |
| 02/03/2022 02:43 | | | | | | | | | | | | | | | | | |  |  |
|  | | | **Aggregate** |  | **Classification** |  | **Coverage** |  | **Number Of Coding References** | |  | **Reference Number** |  | **Coded By Initials** |  | **Modified On** |  |  |  |
|  | **Nodes\\Community engagement\Understanding Community values, cultural norms and beliefs** | | | | | | | | | | | | | | | |  |  |  |
|  | | **Document** | | | | | | | | | | | | | | |  |  |  |
|  | | | **Files\\FGD for CAB #2_2_Transcript** | | | | | | | | | | | | | |  |  |  |
|  |  |  | Yes |  |  |  | 0.1784 |  | 3 | |  | | | | | |  |  |  |
|  | | |  |  |  |  |  |  |  | |  | | | | | | |  |  |
|  | | | | | | | | | | | | 1 |  | AT |  | 01/03/2022 10:23 |  |  |  |
|  | Do you think that could be harmful? In the long run the person, when many people know about your status and other health conditions; will that be harmful to us in anyway? Like stigmatizing us, or saying 'this person is suffering from every health condition'.  P6: About the issue of releasing results like you have said; the truth is getting advice from someone especially if they are not a professional in that field, is very dangerous. Because most people have myths, so many facts they do not know about; you find that one says 'HIV kills' but does not know how to handle the disease. I believe it is dangerous to seek advice from people and there are so many people that do this; they advised each other and said whoever suffered from HIV was bewitched. It wasn’t the HIV+ patient that says this, but the people in the community that they sought advice from, are the ones that gave the wrong information. Those people exist, those who pose risks in regard to that issue. Thank you very much.  P1: Thank you Ma'am. About seeking advice from people; I wouldn’t second it due to personal reasons—or I would say it depends on the health condition of the patient. I will look at my personal experience; the person I asked for advice is the one who almost killed me because whenever anyone came to see me, she says, "Did you know that this person is going to die? She was found HIV+, she is going to die". So some people from whom we seek the advice, are the ones that cause the patient to die. But if the participant of the research is able to understand, just prepare him/her and they will disclose the results to someone they trust. Because one might be my relative but I do not trust him/her, and my other friend who is elsewhere is the one I feel I am comfortable sharing my status with and she is ready to stand with me in every situation. Now my relative to whom you will give the results, will just bury me because whoever comes, "That one has HIV, she is going to die" "She was diagnosed with cancer" yet the cancer patient can survive for even 5years. And one who does not have cancer or HIV, could die of an accident; we have also had COVID, but didn’t have cancer or any other disease. But this relative you told about my issues, is the one that killed me by bringing stigma. Thank you Madam.  P2: About seeking advice from someone has its advantage and disadvantage. You the participant should first get someone who is knowledgeable that you are able to consult, but you shouldn’t leave the participant of the research. But as a participant, you should first consult the researcher but at the end of it all, you have someone you can trust. For instance, we have had challenging health conditions; after being tested, I won’t deny the fact that HIV/AIDS kills, but the major killer was TB because of weak bodies. Now most of the people, because they used to hide and conceal the secret—it is so heavy and burdensome, you even get dizzy while walking because you kept it at heart; this caused TB to kill the mother, father, and even the children. Because of concealing the secret; you are coming from the hospital after being told you had TB, but at home you sleep in the same room with the husband, the breastfeeding baby and another child, so there is going to be infection. You have kept it at heart but you share items with everyone else at home, so you find that the entire household is infected. Yet if you got someone you trust, you could talk to him/her and find a way to avoid all that. But as an individual, think to yourself, 'among all the people I have, who can fight this battle with me?' Just like you see the battle of HIV/AIDS, we won’t brag that us the survivors have fought this battle alone, we have had people who have fought with us. For example, I have ever been into the coma; you leave home without any problem, but you get an attack, and you are in the coma for a couple of days. But because there was someone who was close to me who would rush to 2, since I disclosed my status. Yet if you were as hard as rock, you wouldn’t have survived, so those things have advantages and disadvantages according to my knowledge and experience.  P4: Like I said, these things are not easy; these community matters are not easy because I had my challenges; my wife was taken after I was poverty-stricken. I went to the village and said, "Let me go and hustle in my village. There is no medicine, there is nothing, I will die". But a friend of mine got me from the village and started bringing me to town, and I found a female friend who used to come to see me but I was always worried, "Why is this lady coming here? I am HIV+, why can’t she leave me alone?" But there is a certain man in the community who came and told me, "That woman I see around often lost her husband" so we might think they don’t know our status in the community, but they actually know. So when he gave me that information, I started asking my friend questions and I told her to go for an HIV test. She came back with the results and she was positive, so I told her, "Let us start from there. If you want to stay alive, you have to take medicine". So we kept progressing. One man asked me, "Did you know that the first time you met me, I asked God to give me 5more years?" I asked him how old he was and he said he was 25, "How old are you now?" he said, "I am now 45years old". "Now give thanks to God because you asked him for 5years but he gave you much more, and you are still here". So these matters are not easy, you...<unintelligible> so it depends on how you move on with your life, with God's help I believe everything will be fine. Thank you very much.  M: Thank you.  P6: What I forgot to say, just like you asked if it is good to seek for advice from people in the community. You cannot say it is good but I would say, if you are to seek for advice—if you are in research study about malaria, you know that it is the medical doctor treating malaria. So you could be participating in the research that 2 does not know about, and JCRC knows, but you are on ARVs and you don’t want JCRC to find out. Now if you participate in this research and you want to seek for advice, do not go to someone in the community; instead, come to 2 where there is a professional doctor, and tell him about the issue and what you were told. He will then help you to prepare yourself. But if you consult anyone in the community, it is very challenging; you will get worried. Thank you.  P7: Thank you for this opportunity. I think seeking for advice has 2 sides to it; it is not bad to consult, but it is not good to consult any random person. First ask yourself, 'Who should I ask?' And during counselling when testing HIV patients, you first tell someone, "The results of your blood test are between you and your doctor. And if you have any questions, first consult that very doctor, don’t just ask people because they might not have the right answer" because some answers are right and others are wrong. You might consult someone and they just have positive answers which end up misleading you, and you end up dead. So I think—I have tested and tried it. There is a young adult about 20years | | | | | | | | | | | | | | | |  |  |  |
|  |  |  |  |  |  |  |  |  |  |  |  |  |  |  |  |  |  |  |  |
|  |  |  |  |  |  |  |  |  |  |  |  |  |  |  |  |  |  |  |  |
|  |  |  |  |  |  |  |  |  |  |  |  |  |  |  |  |  |  |  |  |
|  |  |  |  |  |  |  |  |  |  |  |  |  |  |  |  |  |  |  |  |
|  |  |  |  |  |  |  |  |  |  |  |  |  |  |  |  |  |  |  |  |
|  |  |  |  |  |  |  |  |  |  |  |  |  |  |  |  |  |  |  |  |
|  |  |  |  |  |  |  |  |  |  |  |  |  |  |  |  |  |  |  |  |
|  |  |  |  |  |  |  |  |  |  |  |  |  |  |  |  |  |  |  |  |
| Reports\\Coding Summary By Code Report | | | | | | | | | | Page 184 of 409 | | | | | | | |  |  |
| 02/03/2022 02:43 | | | | | | | | | | | | | | | | | |  |  |
|  | | | **Aggregate** |  | **Classification** |  | **Coverage** |  | **Number Of Coding References** | |  | **Reference Number** |  | **Coded By Initials** |  | **Modified On** |  |  |  |
|  | old, he made 21 in September; but no one at home knows that he is positive except me, and his parents who are in the village. His home village is Bujagaali, but he lives in Maganjo. However, no one at home knows that he is HIV+ because he does not disclose to them, and they only say discouraging words yet they don’t know that he is positive. But he comes to speak to me and I say, "Just ignore them". Sometimes I call his father in the village, but we are talking about the son who is here. So it is not bad to ask, but the question is, "Who am I consulting?" you might not ask anyone and instead it affects you because advice could be poisonous and also medicine. So I think you need to be conscious; 'who am I going to consult?' Consult someone who is knowledgeable. We also have—I'm not sure if it was true but I think it was true; we have our system when we are in the community. We tell someone, "If you find that you are positive, do not go around disclosing to anyone, but you just have to ask yourself, 'Who should I disclose to?' because you have to disclose to one or two people. But that girl told me it was only her father and mother that knew her status, but I shouldn’t tell anyone at home, "If I get any problem"—she even gave me her father's contact; I don’t know him at all, and neither does he know me but I have his contact. If she gets any problem, I first call the father; in fact, one time she got a problem, she fell seriously ill with typhoid. But I didn’t tell anyone at home; I called the father in the village and he said, "You know, I think that girl should come" however, she had an Airtel simcard and the father had MTN. He asked me, "Do you know of anyone you trust with an MTN simcard so that I send the child money to come to the village?" He sent the money to my phone, I gave it to the child and she went to the village. She got treatment, stabilized and then came back, but the people at home; even when she gets her medicine, she says, "I don’t want to keep the pills here because those people's words discourage me a lot, but you always encourage me" and I told her, "Ohh we shall change the packaging of the medicine, and use another polythene bag" and she takes her medicine home. "Even if they see it—do not label it because I know it. Do not label it, those at home do not know it. But if they see it, it is my business". So I say it is not good to ask around, but it is good to consult a professional first. | | | | | | | | | | | | | | | |  |  |  |
|  |  |  |  |  |  |  |  |  |  |  |  |  |  |  |  |  |  |  |  |
|  |  |  |  |  |  |  |  |  |  |  |  |  |  |  |  |  |  |  |  |
|  |  | | | | | | | | | | | | | | | |  |  |  |
|  | | | | | | | | | | | | 2 |  | AT |  | 01/03/2022 10:53 |  |  |  |
|  | P6: I was still speaking. Just like you said, you could tell your grandparent because most grandparents have lived long enough to know the family problems, the children. I will give an example; most of the educated people do not believe bathing the children with 'ekyogero' <good-luck charms> and they argue that White people who do not have the same rituals but they survive. What does that mean? The people long ago who did it, did so for a reason but it might instead cause a side-effect on the child. So I would suggest that if you are to share it, share it with someone who will keep your identity discreet; they could call for a meeting and say, "If you give birth to a child with this health condition, just know that it is in the lineage" instead of saying, "So and so has this". So if it is to be shared, it should be spoken of generally and not point out a particular person. Thank you very much. | | | | | | | | | | | | | | | |  |  |  |
|  |  |  |  |  |  |  |  |  |  |  |  |  |  |  |  |  |  |  |  |
|  |  | | | | | | | | | | | | | | | |  |  |  |
|  | | | | | | | | | | | | 3 |  | AT |  | 01/03/2022 10:53 |  |  |  |
|  | P5: In addition to my colleague P6, most people waste their money on traditional healers claiming that so and so was be-witched. But after sitting the clan down or your children, you tell them, "I went to study and we were told that these conditions are genetic" you tell your children, "Do not go to traditional healers asking for goats and so on, yet you know this is a genetic condition". Yes, that is all. | | | | | | | | | | | | | | | |  |  |  |
|  |  | | | | | | | | | | | | | | | |  |  |  |
|  | | | **Files\\FGD for CAB #3_3_Transcript** | | | | | | | | | | | | | |  |  |  |
|  |  |  | Yes |  |  |  | 0.0173 |  | 2 | |  | | | | | |  |  |  |
|  | | |  |  |  |  |  |  |  | |  | | | | | | |  |  |
|  | | | | | | | | | | | | 1 |  | AT |  | 28/02/2022 22:47 |  |  |  |
|  | P1: [moderator]. In a typical village setting, anybody who dies has been bewitched. | | | | | | | | | | | | | | | |  |  |  |
|  |  | | | | | | | | | | | | | | | |  |  |  |
|  | | | | | | | | | | | | 2 |  | AT |  | 28/02/2022 22:48 |  |  |  |
|  | M: Hmm, exactly. And here you are telling them that you want to—  P1: I'll tell you something; my Dad died of liver-related disease. My uncle also died of liver-related disease; I am ...<cuss word deleted> scared of that and I'm actually taking tests because I know—I believe in genetics. But of all those 2 people, none of them according to the villagers [died of liver, died of something else] ...<unintelligible> The other uncle—the other guy who never liked them and that was...<unintelligible>. So when you are bringing that, the reason why I laughed, we are living in a totally different setup from maybe Europe or United States. We are living in a setup where you've died because of some other causes rather than illness even when you have clearly died of this illness. So you see it really depends on the individual who has it, because you are not going to give—even the elder you have picked may never believe— | | | | | | | | | | | | | | | |  |  |  |
|  |  |  |  |  |  |  |  |  |  |  |  |  |  |  |  |  |  |  |  |
|  |  | | | | | | | | | | | | | | | |  |  |  |
|  | | | | | | | | | | | | | | | | | |  |  |
|  | | | | | | | | | | | | | | | | | |  |  |
| Reports\\Coding Summary By Code Report | | | | | | | | | | Page 185 of 409 | | | | | | | |  |  |
| 02/03/2022 02:43 | | | | | | | | | | | | | | | | | |  |  |
|  | | | **Aggregate** |  | **Classification** |  | **Coverage** |  | **Number Of Coding References** | |  | **Reference Number** |  | **Coded By Initials** |  | **Modified On** |  |  |  |
|  | | | **Files\\FGD for CAB #5_5_Transcript** | | | | | | | | | | | | | |  |  |  |
|  |  |  | Yes |  |  |  | 0.1777 |  | 5 | |  | | | | | |  |  |  |
|  | | |  |  |  |  |  |  |  | |  | | | | | | |  |  |
|  | | | | | | | | | | | | 1 |  | AT |  | 01/03/2022 09:06 |  |  |  |
|  | So I wanted to ask, what do you hear about technology in the communities? Do people believe in technology or not? P6.  P6: Technology especially on the side of Muslims; they don’t believe in that because they are taught different doctrines that say, "If you refuse to believe the prophet, you are condemned". An example is, there are some people I advised to go for the paternity test. Sometime back when the Muslim leader was around, there is a lady who had a child and two men claimed paternity of the child. So during that time, they asked, "Who is this woman's husband?" and they said, "It is him" and "Who is the owner of the bed?" "It is him". So he said, "I want to let you know that the owner of this bed is the father of the child". Now that is how the judgement is made in Islam— | | | | | | | | | | | | | | | |  |  |  |
|  |  | | | | | | | | | | | | | | | |  |  |  |
|  | | | | | | | | | | | | 2 |  | AT |  | 01/03/2022 09:06 |  |  |  |
|  | So I said, "Ahh! You could go to the DNA and find out the father of the child" and he said, "You are disagreeing with the prophet!". So I insisted and said, "Religion exists and back in the day, the final verdict of the prophet is what you would go by. But the world is evolving" and there is a word in Quran that says, "Consult others about what you do not know" <Speaks Arabic>. If you fall sick of malaria, you cannot see the germ itself but you go to an expert who will make the diagnosis and tell you, "You are suffering from malaria". Therefore, we should try to accept that there are those who know what we don’t know. Therefore—especially in the Muslim society, they are so rigid about accepting technology because a Muslim might not pick a phone call...<unintelligible> they exist in society. In this day and age, a mobile phone is necessary, it is a need and they just say, "Don’t tell me that!" So we still have that challenge especially among Muslims; ignorance is still high. Therefore, telling people in the community about technology requires thorough teaching in order for them to understand. Also giving examples for instance last evening, I was watching TV with some little girls, and they were talking about HIV/AIDS; they had never seen the situation before. Now when they saw the documentary about HIV/AIDS they were so shocked! "HIV/AIDS really affected these people!" They were frightened. I told them, "This is how they were, but even now that we have medication, one should still stay safe"—there was a gentleman who gave his story, he had HIV for about 30years but he is healthy. He said, "Even if I am still healthy, this disease has still affected me" and it is still evident that he is infected. There is a young adult who said he got HIV because he was promiscuous, and one girl said...<unintelligible>. So people really need to be aware and taught that technology actually exists. | | | | | | | | | | | | | | | |  |  |  |
|  |  |  |  |  |  |  |  |  |  |  |  |  |  |  |  |  |  |  |  |
|  |  |  |  |  |  |  |  |  |  |  |  |  |  |  |  |  |  |  |  |
|  |  | | | | | | | | | | | | | | | |  |  |  |
|  | | | | | | | | | | | | 3 |  | AT |  | 01/03/2022 09:09 |  |  |  |
|  | Indeed. Because—let me first start with the instance of the religious person who was talking the client. Because if you look at the bibles we read, they have editions; having editions means every generation has something different. And the bible says something--a certain leader used it and said, "You will have different children that will change the narrative of their community" you know this person; if I make the statement, it will sound political yet we are in research right now—  <laughs>  Do you understand? And the bible itself says, "we have to be as wise as serpents" it means our brains are always active and that is why we come up with different innovations. So we have different references to show those people that it is not all about the bible, even now as a human, you could be a moving bible because you are the one who puts the bible's instructions into practice. Because it would be in vain if you don’t put it into practice. So I personally think, some people don’t believe in science; some do and others don’t. An example; there are some churches I know that believe HIV is cured through the bible; they pray for it and it is cured. The more one goes to church, the more they deteriorate but when he/she goes home, "My pastor prays!" but the one telling him that he would get cured, is taking his pills. Do you get me? We also have some people who don’t wish others well in religion. So I don’t say it in bad faith, but I give the scenario; but ever since people discovered the truth—you find that one goes to church from Monday to Sunday, he has bible studies but knows that he has to take his medicine at 10pm in order to survive. So this person says, "I shall pray and plead the blood of Jesus, but continue taking my medicine" which means people understand science. You see even the elderly are on tiktok! How about us in research. So I think we should just continue to assure them and show them the advantages, benefits of technology because in this day and age, if an elderly person is able to hold a phone worth shs. 300,000 but another person says, "I don’t need it!" The old person even buys data and takes selfies; you understand? Which means we need to show people that every day that goes by, you cannot remain the same; every month and day that goes by—if you were 2years old—I started by crawling but now I can walk, run, speak, speak to those older than me and even those who have just been born. Which means there is always a new update, and technology is always improving...<unintelligible>Before my mother passed away, I was amazed and asked her, "Mother, don’t you think to yourself how you are in Uganda but you are able to communicate with your brother in America? How do you think things move on?" and she said, "We expected this; actually the clothes and shoes you wear now, are the ones we used to have in the days of the 'twist dance', the old trends like checked clothes, bell bottoms, are just coming back in this current generation".  So I think the more we sensitize people about the benefits, the more they will comprehend and adapt to it, then move on with it. | | | | | | | | | | | | | | | |  |  |  |
|  |  |  |  |  |  |  |  |  |  |  |  |  |  |  |  |  |  |  |  |
|  |  |  |  |  |  |  |  |  |  |  |  |  |  |  |  |  |  |  |  |
|  |  |  |  |  |  |  |  |  |  |  |  |  |  |  |  |  |  |  |  |
|  |  | | | | | | | | | | | | | | | |  |  |  |
|  | | | | | | | | | | | | | | | | | |  |  |
| Reports\\Coding Summary By Code Report | | | | | | | | | | Page 186 of 409 | | | | | | | |  |  |
| 02/03/2022 02:43 | | | | | | | | | | | | | | | | | |  |  |
|  | | | **Aggregate** |  | **Classification** |  | **Coverage** |  | **Number Of Coding References** | |  | **Reference Number** |  | **Coded By Initials** |  | **Modified On** |  |  |  |
|  | | | | | | | | | | | | | | | | | |  |  |
|  | | | | | | | | | | | | 4 |  | AT |  | 01/03/2022 09:08 |  |  |  |
|  | Indeed. Because—let me first start with the instance of the religious person who was talking the client. Because if you look at the bibles we read, they have editions; having editions means every generation has something different. And the bible says something--a certain leader used it and said, "You will have different children that will change the narrative of their community" you know this person; if I make the statement, it will sound political yet we are in research right now—  <laughs>  Do you understand? And the bible itself says, "we have to be as wise as serpents" it means our brains are always active and that is why we come up with different innovations. So we have different references to show those people that it is not all about the bible, even now as a human, you could be a moving bible because you are the one who puts the bible's instructions into practice. Because it would be in vain if you don’t put it into practice. So I personally think, some people don’t believe in science; some do and others don’t. An example; there are some churches I know that believe HIV is cured through the bible; they pray for it and it is cured. The more one goes to church, the more they deteriorate but when he/she goes home, "My pastor prays!" but the one telling him that he would get cured, is taking his pills. Do you get me? We also have some people who don’t wish others well in religion. So I don’t say it in bad faith, but I give the scenario; but ever since people discovered the truth—you find that one goes to church from Monday to Sunday, he has bible studies but knows that he has to take his medicine at 10pm in order to survive. So this person says, "I shall pray and plead the blood of Jesus, but continue taking my medicine" which means people understand science. You see even the elderly are on tiktok! How about us in research. So I think we should just continue to assure them and show them the advantages, benefits of technology because in this day and age, if an elderly person is able to hold a phone worth shs. 300,000 but another person says, "I don’t need it!" The old person even buys data and takes selfies; you understand? Which means we need to show people that every day that goes by, you cannot remain the same; every month and day that goes by—if you were 2years old—I started by crawling but now I can walk, run, speak, speak to those older than me and even those who have just been born. Which means there is always a new update, and technology is always improving...<unintelligible>Before my mother passed away, I was amazed and asked her, "Mother, don’t you think to yourself how you are in Uganda but you are able to communicate with your brother in America? How do you think things move on?" and she said, "We expected this; actually the clothes and shoes you wear now, are the ones we used to have in the days of the 'twist dance', the old trends like checked clothes, bell bottoms, are just coming back in this current generation".  So I think the more we sensitize people about the benefits, the more they will comprehend and adapt to it, then move on with it. | | | | | | | | | | | | | | | |  |  |  |
|  |  |  |  |  |  |  |  |  |  |  |  |  |  |  |  |  |  |  |  |
|  |  |  |  |  |  |  |  |  |  |  |  |  |  |  |  |  |  |  |  |
|  |  |  |  |  |  |  |  |  |  |  |  |  |  |  |  |  |  |  |  |
|  |  | | | | | | | | | | | | | | | |  |  |  |
|  | | | | | | | | | | | | | | | | | |  |  |
|  | | | | | | | | | | | | | | | | | |  |  |
|  | | | | | | | | | | | | | | | | | |  |  |
|  | | | | | | | | | | | | | | | | | |  |  |
|  | | | | | | | | | | | | | | | | | |  |  |
| Reports\\Coding Summary By Code Report | | | | | | | | | | Page 187 of 409 | | | | | | | |  |  |
| 02/03/2022 02:43 | | | | | | | | | | | | | | | | | |  |  |
|  | | | **Aggregate** |  | **Classification** |  | **Coverage** |  | **Number Of Coding References** | |  | **Reference Number** |  | **Coded By Initials** |  | **Modified On** |  |  |  |
|  | | | | | | | | | | | | | | | | | |  |  |
|  | | | | | | | | | | | | 5 |  | AT |  | 01/03/2022 09:11 |  |  |  |
|  | M: Yes, will the people accept the results from the technology, especially those that say, 'in the future, this is what will happen'?  P7: I think they will accept the technology; the answer is actually yes and no. Because if you look at the data in this country, majority of the population is young people and they are blessed to live in the Information technology era and they strongly believe in it. One thing I admire about the young people is, while we were still in school, we didn’t study the Entrepreneurship course. And it gave us hard time to know what 'business' is; and these children who have studied entrepreneurship are well versed in finance. While we were in school, we thought we just had to study and get jobs and then pay monthly bills but things have changed. The children who have studied the entrepreneurship course, they are versatile and change with the trends, and since it is majority of the population, they are well versed with science and prove the facts. Since they are the majority, those who do not want to adapt to the computer age will remain backward; we shall be just like the ones...<unintelligible> because we remained in the analog age yet the world is evolving.  We didn’t think a time would come where we are 50 people on a zoom meeting; but that is where the world is headed. In 5-10years, the entire world will run on technology, so since the younger generations have had that chance, they have grasped it faster. They will just go ahead and check, "What are the research results?" Take a look at the vaccinations for COVID, they just press this and the certificate number is given because we used to think the vaccination card was the certificate, which isn’t the case. So I believe they are in the technology age and in the future, if they experience any symptoms, they first make a diagnosis themselves using technology and then come to the doctor. And the doctor will have to also be an IT expert, not just a general doctor, so I think they will accept very well and continue with it. Another reason I say no is, for us who are rigid will just continue arguing and disagree, "It used to be like this". One time I was sent, "Go to Mpelerwe, our home is just next to the big tree" but the tree is no longer there; instead there are shops, etc, and yet this person is giving you directions based on what used to be there. So things have changed drastically, whether we like it or not; some of us will be left hanging, because the world is moving at a terrific speed. You just find yourself...<unintelligible> we used to pick up phone calls like this unlike now, but these days the young people are always on their phones, trying to keep up with the current trends. During our days—I wonder if the landline phones still exist; I think they are found in offices. So I think in the coming time, someone will just tab a button and all the data is presented.  P2: Now about these people's perception of science and technology; majority believe in science but others don’t believe in science. But the challenge of being rigid is—what makes them rigid when it comes to technology is, they put the blame on people, "We don’t trust the government, they are planning to do this" others believe conspiracy theories, "The whites just want to get rid of us and then take over our country". And take an example of the COVID vaccinations, "I don’t trust the first vaccine, I want Johnson and Johnson" Who manufactures it? It is for the whites; we were told". So don’t you see that there are information gaps, and they claim, "You are vaccinated so in a few years, all of you are going to die!"  ...<unintelligible> So you find that those are the challenges we have. Some of them still say, "We just check our children to know if they belong to our family" people don’t believe in that anymore. So if you don’t explain to them, some will believe in science and others won’t. But there are some who will never change because they still believe, "The owner of the bed and woman is the owner of the child" that is the law in Uganda! Until you prove beyond reasonable doubt that the child is not yours. But the child born in your house, is yours and nothing else! ...<Noise>  <unintelligible>  So you find that the court says this, the DNA says otherwise. So that is the challenge of science but our people have accepted science especially if you sensitize them. One may refuse to get vaccinated for COVID but thinks to oneself, "These children were vaccinated for the 9 child-killer diseases. Every child is immunized but we don’t know where those vaccines are manufactured" then eventually he comes back and says, "I think science is necessary! Let us just go ahead with everything". So people believe in science, some don’t—they are mixed in the community. But others are rigid and don’t believe in science. | | | | | | | | | | | | | | | |  |  |  |
|  |  |  |  |  |  |  |  |  |  |  |  |  |  |  |  |  |  |  |  |
|  |  |  |  |  |  |  |  |  |  |  |  |  |  |  |  |  |  |  |  |
|  |  |  |  |  |  |  |  |  |  |  |  |  |  |  |  |  |  |  |  |
|  |  |  |  |  |  |  |  |  |  |  |  |  |  |  |  |  |  |  |  |
|  |  |  |  |  |  |  |  |  |  |  |  |  |  |  |  |  |  |  |  |
|  |  | | | | | | | | | | | | | | | |  |  |  |
|  | | | | | | | | | | | | | | | | | |  |  |
|  | | | | | | | | | | | | | | | | | |  |  |
|  | | | | | | | | | | | | | | | | | |  |  |
|  | | | | | | | | | | | | | | | | | |  |  |
| Reports\\Coding Summary By Code Report | | | | | | | | | | Page 188 of 409 | | | | | | | |  |  |
| 02/03/2022 02:43 | | | | | | | | | | | | | | | | | |  |  |
|  | | | **Aggregate** |  | **Classification** |  | **Coverage** |  | **Number Of Coding References** | |  | **Reference Number** |  | **Coded By Initials** |  | **Modified On** |  |  |  |
|  | **Nodes\\Community engagement\Understanding Community values, cultural norms and beliefs\Association of illnesses with bewitchment** | | | | | | | | | | | | | | | |  |  |  |
|  | | **Document** | | | | | | | | | | | | | | |  |  |  |
|  | | | **Files\\FGD for CAB #2_2_Transcript** | | | | | | | | | | | | | |  |  |  |
|  |  |  | No |  |  |  | 0.1636 |  | 2 | |  | | | | | |  |  |  |
|  | | |  |  |  |  |  |  |  | |  | | | | | | |  |  |
|  | | | | | | | | | | | | 1 |  | AT |  | 01/03/2022 10:23 |  |  |  |
|  | Do you think that could be harmful? In the long run the person, when many people know about your status and other health conditions; will that be harmful to us in anyway? Like stigmatizing us, or saying 'this person is suffering from every health condition'.  P6: About the issue of releasing results like you have said; the truth is getting advice from someone especially if they are not a professional in that field, is very dangerous. Because most people have myths, so many facts they do not know about; you find that one says 'HIV kills' but does not know how to handle the disease. I believe it is dangerous to seek advice from people and there are so many people that do this; they advised each other and said whoever suffered from HIV was bewitched. It wasn’t the HIV+ patient that says this, but the people in the community that they sought advice from, are the ones that gave the wrong information. Those people exist, those who pose risks in regard to that issue. Thank you very much.  P1: Thank you Ma'am. About seeking advice from people; I wouldn’t second it due to personal reasons—or I would say it depends on the health condition of the patient. I will look at my personal experience; the person I asked for advice is the one who almost killed me because whenever anyone came to see me, she says, "Did you know that this person is going to die? She was found HIV+, she is going to die". So some people from whom we seek the advice, are the ones that cause the patient to die. But if the participant of the research is able to understand, just prepare him/her and they will disclose the results to someone they trust. Because one might be my relative but I do not trust him/her, and my other friend who is elsewhere is the one I feel I am comfortable sharing my status with and she is ready to stand with me in every situation. Now my relative to whom you will give the results, will just bury me because whoever comes, "That one has HIV, she is going to die" "She was diagnosed with cancer" yet the cancer patient can survive for even 5years. And one who does not have cancer or HIV, could die of an accident; we have also had COVID, but didn’t have cancer or any other disease. But this relative you told about my issues, is the one that killed me by bringing stigma. Thank you Madam.  P2: About seeking advice from someone has its advantage and disadvantage. You the participant should first get someone who is knowledgeable that you are able to consult, but you shouldn’t leave the participant of the research. But as a participant, you should first consult the researcher but at the end of it all, you have someone you can trust. For instance, we have had challenging health conditions; after being tested, I won’t deny the fact that HIV/AIDS kills, but the major killer was TB because of weak bodies. Now most of the people, because they used to hide and conceal the secret—it is so heavy and burdensome, you even get dizzy while walking because you kept it at heart; this caused TB to kill the mother, father, and even the children. Because of concealing the secret; you are coming from the hospital after being told you had TB, but at home you sleep in the same room with the husband, the breastfeeding baby and another child, so there is going to be infection. You have kept it at heart but you share items with everyone else at home, so you find that the entire household is infected. Yet if you got someone you trust, you could talk to him/her and find a way to avoid all that. But as an individual, think to yourself, 'among all the people I have, who can fight this battle with me?' Just like you see the battle of HIV/AIDS, we won’t brag that us the survivors have fought this battle alone, we have had people who have fought with us. For example, I have ever been into the coma; you leave home without any problem, but you get an attack, and you are in the coma for a couple of days. But because there was someone who was close to me who would rush to 2, since I disclosed my status. Yet if you were as hard as rock, you wouldn’t have survived, so those things have advantages and disadvantages according to my knowledge and experience.  P4: Like I said, these things are not easy; these community matters are not easy because I had my challenges; my wife was taken after I was poverty-stricken. I went to the village and said, "Let me go and hustle in my village. There is no medicine, there is nothing, I will die". But a friend of mine got me from the village and started bringing me to town, and I found a female friend who used to come to see me but I was always worried, "Why is this lady coming here? I am HIV+, why can’t she leave me alone?" But there is a certain man in the community who came and told me, "That woman I see around often lost her husband" so we might think they don’t know our status in the community, but they actually know. So when he gave me that information, I started asking my friend questions and I told her to go for an HIV test. She came back with the results and she was positive, so I told her, "Let us start from there. If you want to stay alive, you have to take medicine". So we kept progressing. One man asked me, "Did you know that the first time you met me, I asked God to give me 5more years?" I asked him how old he was and he said he was 25, "How old are you now?" he said, "I am now 45years old". "Now give thanks to God because you asked him for 5years but he gave you much more, and you are still here". So these matters are not easy, you...<unintelligible> so it depends on how you move on with your life, with God's help I believe everything will be fine. Thank you very much.  M: Thank you.  P6: What I forgot to say, just like you asked if it is good to seek for advice from people in the community. You cannot say it is good but I would say, if you are to seek for advice—if you are in research study about malaria, you know that it is the medical doctor treating malaria. So you could be participating in the research that 2 does not know about, and JCRC knows, but you are on ARVs and you don’t want JCRC to find out. Now if you participate in this research and you want to seek for advice, do not go to someone in the community; instead, come to 2 where there is a professional doctor, and tell him about the issue and what you were told. He will then help you to prepare yourself. But if you consult anyone in the community, it is very challenging; you will get worried. Thank you.  P7: Thank you for this opportunity. I think seeking for advice has 2 sides to it; it is not bad to consult, but it is not good to consult any random person. First ask yourself, 'Who should I ask?' And during counselling when testing HIV patients, you first tell someone, "The results of your blood test are between you and your doctor. And if you have any questions, first consult that very doctor, don’t just ask people because they might not have the right answer" because some answers are right and others are wrong. You might consult someone and they just have | | | | | | | | | | | | | | | |  |  |  |
|  |  |  |  |  |  |  |  |  |  |  |  |  |  |  |  |  |  |  |  |
|  |  |  |  |  |  |  |  |  |  |  |  |  |  |  |  |  |  |  |  |
|  |  |  |  |  |  |  |  |  |  |  |  |  |  |  |  |  |  |  |  |
|  |  |  |  |  |  |  |  |  |  |  |  |  |  |  |  |  |  |  |  |
|  |  |  |  |  |  |  |  |  |  |  |  |  |  |  |  |  |  |  |  |
|  |  |  |  |  |  |  |  |  |  |  |  |  |  |  |  |  |  |  |  |
|  |  |  |  |  |  |  |  |  |  |  |  |  |  |  |  |  |  |  |  |
|  |  |  |  |  |  |  |  |  |  |  |  |  |  |  |  |  |  |  |  |
| Reports\\Coding Summary By Code Report | | | | | | | | | | Page 189 of 409 | | | | | | | |  |  |
| 02/03/2022 02:43 | | | | | | | | | | | | | | | | | |  |  |
|  | | | **Aggregate** |  | **Classification** |  | **Coverage** |  | **Number Of Coding References** | |  | **Reference Number** |  | **Coded By Initials** |  | **Modified On** |  |  |  |
|  | positive answers which end up misleading you, and you end up dead. So I think—I have tested and tried it. There is a young adult about 20years old, he made 21 in September; but no one at home knows that he is positive except me, and his parents who are in the village. His home village is Bujagaali, but he lives in Maganjo. However, no one at home knows that he is HIV+ because he does not disclose to them, and they only say discouraging words yet they don’t know that he is positive. But he comes to speak to me and I say, "Just ignore them". Sometimes I call his father in the village, but we are talking about the son who is here. So it is not bad to ask, but the question is, "Who am I consulting?" you might not ask anyone and instead it affects you because advice could be poisonous and also medicine. So I think you need to be conscious; 'who am I going to consult?' Consult someone who is knowledgeable. We also have—I'm not sure if it was true but I think it was true; we have our system when we are in the community. We tell someone, "If you find that you are positive, do not go around disclosing to anyone, but you just have to ask yourself, 'Who should I disclose to?' because you have to disclose to one or two people. But that girl told me it was only her father and mother that knew her status, but I shouldn’t tell anyone at home, "If I get any problem"—she even gave me her father's contact; I don’t know him at all, and neither does he know me but I have his contact. If she gets any problem, I first call the father; in fact, one time she got a problem, she fell seriously ill with typhoid. But I didn’t tell anyone at home; I called the father in the village and he said, "You know, I think that girl should come" however, she had an Airtel simcard and the father had MTN. He asked me, "Do you know of anyone you trust with an MTN simcard so that I send the child money to come to the village?" He sent the money to my phone, I gave it to the child and she went to the village. She got treatment, stabilized and then came back, but the people at home; even when she gets her medicine, she says, "I don’t want to keep the pills here because those people's words discourage me a lot, but you always encourage me" and I told her, "Ohh we shall change the packaging of the medicine, and use another polythene bag" and she takes her medicine home. "Even if they see it—do not label it because I know it. Do not label it, those at home do not know it. But if they see it, it is my business". So I say it is not good to ask around, but it is good to consult a professional first. | | | | | | | | | | | | | | | |  |  |  |
|  |  |  |  |  |  |  |  |  |  |  |  |  |  |  |  |  |  |  |  |
|  |  |  |  |  |  |  |  |  |  |  |  |  |  |  |  |  |  |  |  |
|  |  | | | | | | | | | | | | | | | |  |  |  |
|  | | | | | | | | | | | | 2 |  | AT |  | 01/03/2022 10:53 |  |  |  |
|  | P5: In addition to my colleague P6, most people waste their money on traditional healers claiming that so and so was be-witched. But after sitting the clan down or your children, you tell them, "I went to study and we were told that these conditions are genetic" you tell your children, "Do not go to traditional healers asking for goats and so on, yet you know this is a genetic condition". Yes, that is all. | | | | | | | | | | | | | | | |  |  |  |
|  |  | | | | | | | | | | | | | | | |  |  |  |
|  | **Nodes\\Community engagement\Understanding Community values, cultural norms and beliefs\cultural beliefs** | | | | | | | | | | | | | | | |  |  |  |
|  | | **Document** | | | | | | | | | | | | | | |  |  |  |
|  | | | **Files\\FGD for CAB #2_2_Transcript** | | | | | | | | | | | | | |  |  |  |
|  |  |  | No |  |  |  | 0.0147 |  | 1 | |  | | | | | |  |  |  |
|  | | |  |  |  |  |  |  |  | |  | | | | | | |  |  |
|  | | | | | | | | | | | | 1 |  | AT |  | 01/03/2022 10:53 |  |  |  |
|  | P6: I was still speaking. Just like you said, you could tell your grandparent because most grandparents have lived long enough to know the family problems, the children. I will give an example; most of the educated people do not believe bathing the children with 'ekyogero' <good-luck charms> and they argue that White people who do not have the same rituals but they survive. What does that mean? The people long ago who did it, did so for a reason but it might instead cause a side-effect on the child. So I would suggest that if you are to share it, share it with someone who will keep your identity discreet; they could call for a meeting and say, "If you give birth to a child with this health condition, just know that it is in the lineage" instead of saying, "So and so has this". So if it is to be shared, it should be spoken of generally and not point out a particular person. Thank you very much. | | | | | | | | | | | | | | | |  |  |  |
|  |  |  |  |  |  |  |  |  |  |  |  |  |  |  |  |  |  |  |  |
|  |  | | | | | | | | | | | | | | | |  |  |  |
|  | | | | | | | | | | | | | | | | | |  |  |
|  | | | | | | | | | | | | | | | | | |  |  |
| Reports\\Coding Summary By Code Report | | | | | | | | | | Page 190 of 409 | | | | | | | |  |  |
| 02/03/2022 02:43 | | | | | | | | | | | | | | | | | |  |  |
|  | | | **Aggregate** |  | **Classification** |  | **Coverage** |  | **Number Of Coding References** | |  | **Reference Number** |  | **Coded By Initials** |  | **Modified On** |  |  |  |
|  | **Nodes\\Community engagement\Understanding Community values, cultural norms and beliefs\people accept the results from technology similar to how COVID vaccination certificate is obtained.** | | | | | | | | | | | | | | | |  |  |  |
|  | | **Document** | | | | | | | | | | | | | | |  |  |  |
|  | | | **Files\\FGD for CAB #5_5_Transcript** | | | | | | | | | | | | | |  |  |  |
|  |  |  | No |  |  |  | 0.0806 |  | 1 | |  | | | | | |  |  |  |
|  | | |  |  |  |  |  |  |  | |  | | | | | | |  |  |
|  | | | | | | | | | | | | 1 |  | AT |  | 01/03/2022 09:11 |  |  |  |
|  | M: Yes, will the people accept the results from the technology, especially those that say, 'in the future, this is what will happen'?  P7: I think they will accept the technology; the answer is actually yes and no. Because if you look at the data in this country, majority of the population is young people and they are blessed to live in the Information technology era and they strongly believe in it. One thing I admire about the young people is, while we were still in school, we didn’t study the Entrepreneurship course. And it gave us hard time to know what 'business' is; and these children who have studied entrepreneurship are well versed in finance. While we were in school, we thought we just had to study and get jobs and then pay monthly bills but things have changed. The children who have studied the entrepreneurship course, they are versatile and change with the trends, and since it is majority of the population, they are well versed with science and prove the facts. Since they are the majority, those who do not want to adapt to the computer age will remain backward; we shall be just like the ones...<unintelligible> because we remained in the analog age yet the world is evolving.  We didn’t think a time would come where we are 50 people on a zoom meeting; but that is where the world is headed. In 5-10years, the entire world will run on technology, so since the younger generations have had that chance, they have grasped it faster. They will just go ahead and check, "What are the research results?" Take a look at the vaccinations for COVID, they just press this and the certificate number is given because we used to think the vaccination card was the certificate, which isn’t the case. So I believe they are in the technology age and in the future, if they experience any symptoms, they first make a diagnosis themselves using technology and then come to the doctor. And the doctor will have to also be an IT expert, not just a general doctor, so I think they will accept very well and continue with it. Another reason I say no is, for us who are rigid will just continue arguing and disagree, "It used to be like this". One time I was sent, "Go to Mpelerwe, our home is just next to the big tree" but the tree is no longer there; instead there are shops, etc, and yet this person is giving you directions based on what used to be there. So things have changed drastically, whether we like it or not; some of us will be left hanging, because the world is moving at a terrific speed. You just find yourself...<unintelligible> we used to pick up phone calls like this unlike now, but these days the young people are always on their phones, trying to keep up with the current trends. During our days—I wonder if the landline phones still exist; I think they are found in offices. So I think in the coming time, someone will just tab a button and all the data is presented.  P2: Now about these people's perception of science and technology; majority believe in science but others don’t believe in science. But the challenge of being rigid is—what makes them rigid when it comes to technology is, they put the blame on people, "We don’t trust the government, they are planning to do this" others believe conspiracy theories, "The whites just want to get rid of us and then take over our country". And take an example of the COVID vaccinations, "I don’t trust the first vaccine, I want Johnson and Johnson" Who manufactures it? It is for the whites; we were told". So don’t you see that there are information gaps, and they claim, "You are vaccinated so in a few years, all of you are going to die!"  ...<unintelligible> So you find that those are the challenges we have. Some of them still say, "We just check our children to know if they belong to our family" people don’t believe in that anymore. So if you don’t explain to them, some will believe in science and others won’t. But there are some who will never change because they still believe, "The owner of the bed and woman is the owner of the child" that is the law in Uganda! Until you prove beyond reasonable doubt that the child is not yours. But the child born in your house, is yours and nothing else! ...<Noise>  <unintelligible>  So you find that the court says this, the DNA says otherwise. So that is the challenge of science but our people have accepted science especially if you sensitize them. One may refuse to get vaccinated for COVID but thinks to oneself, "These children were vaccinated for the 9 child-killer diseases. Every child is immunized but we don’t know where those vaccines are manufactured" then eventually he comes back and says, "I think science is necessary! Let us just go ahead with everything". So people believe in science, some don’t—they are mixed in the community. But others are rigid and don’t believe in science. | | | | | | | | | | | | | | | |  |  |  |
|  |  |  |  |  |  |  |  |  |  |  |  |  |  |  |  |  |  |  |  |
|  |  |  |  |  |  |  |  |  |  |  |  |  |  |  |  |  |  |  |  |
|  |  |  |  |  |  |  |  |  |  |  |  |  |  |  |  |  |  |  |  |
|  |  |  |  |  |  |  |  |  |  |  |  |  |  |  |  |  |  |  |  |
|  |  |  |  |  |  |  |  |  |  |  |  |  |  |  |  |  |  |  |  |
|  |  | | | | | | | | | | | | | | | |  |  |  |
|  | | | | | | | | | | | | | | | | | |  |  |
|  | | | | | | | | | | | | | | | | | |  |  |
| Reports\\Coding Summary By Code Report | | | | | | | | | | Page 191 of 409 | | | | | | | |  |  |
| 02/03/2022 02:43 | | | | | | | | | | | | | | | | | |  |  |
|  | | | **Aggregate** |  | **Classification** |  | **Coverage** |  | **Number Of Coding References** | |  | **Reference Number** |  | **Coded By Initials** |  | **Modified On** |  |  |  |
|  | **Nodes\\Community engagement\Understanding Community values, cultural norms and beliefs\Religious beliefs towards emerging technologies** | | | | | | | | | | | | | | | |  |  |  |
|  | | **Document** | | | | | | | | | | | | | | |  |  |  |
|  | | | **Files\\FGD for CAB #3_3_Transcript** | | | | | | | | | | | | | |  |  |  |
|  |  |  | Yes |  |  |  | 0.0173 |  | 2 | |  | | | | | |  |  |  |
|  | | |  |  |  |  |  |  |  | |  | | | | | | |  |  |
|  | | | | | | | | | | | | 1 |  | AT |  | 28/02/2022 22:47 |  |  |  |
|  | P1: [moderator]. In a typical village setting, anybody who dies has been bewitched. | | | | | | | | | | | | | | | |  |  |  |
|  |  | | | | | | | | | | | | | | | |  |  |  |
|  | | | | | | | | | | | | 2 |  | AT |  | 28/02/2022 22:48 |  |  |  |
|  | M: Hmm, exactly. And here you are telling them that you want to—  P1: I'll tell you something; my Dad died of liver-related disease. My uncle also died of liver-related disease; I am ...<cuss word deleted> scared of that and I'm actually taking tests because I know—I believe in genetics. But of all those 2 people, none of them according to the villagers [died of liver, died of something else] ...<unintelligible> The other uncle—the other guy who never liked them and that was...<unintelligible>. So when you are bringing that, the reason why I laughed, we are living in a totally different setup from maybe Europe or United States. We are living in a setup where you've died because of some other causes rather than illness even when you have clearly died of this illness. So you see it really depends on the individual who has it, because you are not going to give—even the elder you have picked may never believe— | | | | | | | | | | | | | | | |  |  |  |
|  |  |  |  |  |  |  |  |  |  |  |  |  |  |  |  |  |  |  |  |
|  |  | | | | | | | | | | | | | | | |  |  |  |
|  | | | **Files\\FGD for CAB #5_5_Transcript** | | | | | | | | | | | | | |  |  |  |
|  |  |  | Yes |  |  |  | 0.0970 |  | 3 | |  | | | | | |  |  |  |
|  | | |  |  |  |  |  |  |  | |  | | | | | | |  |  |
|  | | | | | | | | | | | | 1 |  | AT |  | 01/03/2022 09:06 |  |  |  |
|  | So I wanted to ask, what do you hear about technology in the communities? Do people believe in technology or not? P6.  P6: Technology especially on the side of Muslims; they don’t believe in that because they are taught different doctrines that say, "If you refuse to believe the prophet, you are condemned". An example is, there are some people I advised to go for the paternity test. Sometime back when the Muslim leader was around, there is a lady who had a child and two men claimed paternity of the child. So during that time, they asked, "Who is this woman's husband?" and they said, "It is him" and "Who is the owner of the bed?" "It is him". So he said, "I want to let you know that the owner of this bed is the father of the child". Now that is how the judgement is made in Islam— | | | | | | | | | | | | | | | |  |  |  |
|  |  | | | | | | | | | | | | | | | |  |  |  |
|  | | | | | | | | | | | | 2 |  | AT |  | 01/03/2022 09:06 |  |  |  |
|  | So I said, "Ahh! You could go to the DNA and find out the father of the child" and he said, "You are disagreeing with the prophet!". So I insisted and said, "Religion exists and back in the day, the final verdict of the prophet is what you would go by. But the world is evolving" and there is a word in Quran that says, "Consult others about what you do not know" <Speaks Arabic>. If you fall sick of malaria, you cannot see the germ itself but you go to an expert who will make the diagnosis and tell you, "You are suffering from malaria". Therefore, we should try to accept that there are those who know what we don’t know. Therefore—especially in the Muslim society, they are so rigid about accepting technology because a Muslim might not pick a phone call...<unintelligible> they exist in society. In this day and age, a mobile phone is necessary, it is a need and they just say, "Don’t tell me that!" So we still have that challenge especially among Muslims; ignorance is still high. Therefore, telling people in the community about technology requires thorough teaching in order for them to understand. Also giving examples for instance last evening, I was watching TV with some little girls, and they were talking about HIV/AIDS; they had never seen the situation before. Now when they saw the documentary about HIV/AIDS they were so shocked! "HIV/AIDS really affected these people!" They were frightened. I told them, "This is how they were, but even now that we have medication, one should still stay safe"—there was a gentleman who gave his story, he had HIV for about 30years but he is healthy. He said, "Even if I am still healthy, this disease has still affected me" and it is still evident that he is infected. There is a young adult who said he got HIV because he was promiscuous, and one girl said...<unintelligible>. So people really need to be aware and taught that technology actually exists. | | | | | | | | | | | | | | | |  |  |  |
|  |  |  |  |  |  |  |  |  |  |  |  |  |  |  |  |  |  |  |  |
|  |  |  |  |  |  |  |  |  |  |  |  |  |  |  |  |  |  |  |  |
|  |  | | | | | | | | | | | | | | | |  |  |  |
|  | | | | | | | | | | | | | | | | | |  |  |
| Reports\\Coding Summary By Code Report | | | | | | | | | | Page 192 of 409 | | | | | | | |  |  |
| 02/03/2022 02:43 | | | | | | | | | | | | | | | | | |  |  |
|  | | | **Aggregate** |  | **Classification** |  | **Coverage** |  | **Number Of Coding References** | |  | **Reference Number** |  | **Coded By Initials** |  | **Modified On** |  |  |  |
|  | | | | | | | | | | | | | | | | | |  |  |
|  | | | | | | | | | | | | 3 |  | AT |  | 01/03/2022 09:09 |  |  |  |
|  | Indeed. Because—let me first start with the instance of the religious person who was talking the client. Because if you look at the bibles we read, they have editions; having editions means every generation has something different. And the bible says something--a certain leader used it and said, "You will have different children that will change the narrative of their community" you know this person; if I make the statement, it will sound political yet we are in research right now—  <laughs>  Do you understand? And the bible itself says, "we have to be as wise as serpents" it means our brains are always active and that is why we come up with different innovations. So we have different references to show those people that it is not all about the bible, even now as a human, you could be a moving bible because you are the one who puts the bible's instructions into practice. Because it would be in vain if you don’t put it into practice. So I personally think, some people don’t believe in science; some do and others don’t. An example; there are some churches I know that believe HIV is cured through the bible; they pray for it and it is cured. The more one goes to church, the more they deteriorate but when he/she goes home, "My pastor prays!" but the one telling him that he would get cured, is taking his pills. Do you get me? We also have some people who don’t wish others well in religion. So I don’t say it in bad faith, but I give the scenario; but ever since people discovered the truth—you find that one goes to church from Monday to Sunday, he has bible studies but knows that he has to take his medicine at 10pm in order to survive. So this person says, "I shall pray and plead the blood of Jesus, but continue taking my medicine" which means people understand science. You see even the elderly are on tiktok! How about us in research. So I think we should just continue to assure them and show them the advantages, benefits of technology because in this day and age, if an elderly person is able to hold a phone worth shs. 300,000 but another person says, "I don’t need it!" The old person even buys data and takes selfies; you understand? Which means we need to show people that every day that goes by, you cannot remain the same; every month and day that goes by—if you were 2years old—I started by crawling but now I can walk, run, speak, speak to those older than me and even those who have just been born. Which means there is always a new update, and technology is always improving...<unintelligible>Before my mother passed away, I was amazed and asked her, "Mother, don’t you think to yourself how you are in Uganda but you are able to communicate with your brother in America? How do you think things move on?" and she said, "We expected this; actually the clothes and shoes you wear now, are the ones we used to have in the days of the 'twist dance', the old trends like checked clothes, bell bottoms, are just coming back in this current generation".  So I think the more we sensitize people about the benefits, the more they will comprehend and adapt to it, then move on with it. | | | | | | | | | | | | | | | |  |  |  |
|  |  |  |  |  |  |  |  |  |  |  |  |  |  |  |  |  |  |  |  |
|  |  |  |  |  |  |  |  |  |  |  |  |  |  |  |  |  |  |  |  |
|  |  |  |  |  |  |  |  |  |  |  |  |  |  |  |  |  |  |  |  |
|  |  | | | | | | | | | | | | | | | |  |  |  |
|  | | | | | | | | | | | | | | | | | |  |  |
|  | | | | | | | | | | | | | | | | | |  |  |
|  | | | | | | | | | | | | | | | | | |  |  |
|  | | | | | | | | | | | | | | | | | |  |  |
|  | | | | | | | | | | | | | | | | | |  |  |
| Reports\\Coding Summary By Code Report | | | | | | | | | | Page 193 of 409 | | | | | | | |  |  |
| 02/03/2022 02:43 | | | | | | | | | | | | | | | | | |  |  |
|  | | | **Aggregate** |  | **Classification** |  | **Coverage** |  | **Number Of Coding References** | |  | **Reference Number** |  | **Coded By Initials** |  | **Modified On** |  |  |  |
|  | **Nodes\\Community engagement\Understanding Community values, cultural norms and beliefs\Religious beliefs towards emerging technologies\Born again beliefs** | | | | | | | | | | | | | | | |  |  |  |
|  | | **Document** | | | | | | | | | | | | | | |  |  |  |
|  | | | **Files\\FGD for CAB #5_5_Transcript** | | | | | | | | | | | | | |  |  |  |
|  |  |  | No |  |  |  | 0.0519 |  | 1 | |  | | | | | |  |  |  |
|  | | |  |  |  |  |  |  |  | |  | | | | | | |  |  |
|  | | | | | | | | | | | | 1 |  | AT |  | 01/03/2022 09:09 |  |  |  |
|  | Indeed. Because—let me first start with the instance of the religious person who was talking the client. Because if you look at the bibles we read, they have editions; having editions means every generation has something different. And the bible says something--a certain leader used it and said, "You will have different children that will change the narrative of their community" you know this person; if I make the statement, it will sound political yet we are in research right now—  <laughs>  Do you understand? And the bible itself says, "we have to be as wise as serpents" it means our brains are always active and that is why we come up with different innovations. So we have different references to show those people that it is not all about the bible, even now as a human, you could be a moving bible because you are the one who puts the bible's instructions into practice. Because it would be in vain if you don’t put it into practice. So I personally think, some people don’t believe in science; some do and others don’t. An example; there are some churches I know that believe HIV is cured through the bible; they pray for it and it is cured. The more one goes to church, the more they deteriorate but when he/she goes home, "My pastor prays!" but the one telling him that he would get cured, is taking his pills. Do you get me? We also have some people who don’t wish others well in religion. So I don’t say it in bad faith, but I give the scenario; but ever since people discovered the truth—you find that one goes to church from Monday to Sunday, he has bible studies but knows that he has to take his medicine at 10pm in order to survive. So this person says, "I shall pray and plead the blood of Jesus, but continue taking my medicine" which means people understand science. You see even the elderly are on tiktok! How about us in research. So I think we should just continue to assure them and show them the advantages, benefits of technology because in this day and age, if an elderly person is able to hold a phone worth shs. 300,000 but another person says, "I don’t need it!" The old person even buys data and takes selfies; you understand? Which means we need to show people that every day that goes by, you cannot remain the same; every month and day that goes by—if you were 2years old—I started by crawling but now I can walk, run, speak, speak to those older than me and even those who have just been born. Which means there is always a new update, and technology is always improving...<unintelligible>Before my mother passed away, I was amazed and asked her, "Mother, don’t you think to yourself how you are in Uganda but you are able to communicate with your brother in America? How do you think things move on?" and she said, "We expected this; actually the clothes and shoes you wear now, are the ones we used to have in the days of the 'twist dance', the old trends like checked clothes, bell bottoms, are just coming back in this current generation".  So I think the more we sensitize people about the benefits, the more they will comprehend and adapt to it, then move on with it. | | | | | | | | | | | | | | | |  |  |  |

| **Nodes\\Community engagement\Understanding Community values, cultural norms and beliefs\Religious beliefs towards emerging technologies\In a typical village setting, anybody who dies has been bewitched** | | | | | | | | | | | | | | | |  |  |
| --- | --- | --- | --- | --- | --- | --- | --- | --- | --- | --- | --- | --- | --- | --- | --- | --- | --- |
|  | **Document** | | | | | | | | | | | | | | | |  |
|  | | **Files\\FGD for CAB #3_3_Transcript** | | | | | | | | | | | | | | |  |
|  |  | No |  |  |  | 0.0173 |  | 2 | |  | | | | | | |  |
|  | |  |  |  |  |  |  |  | |  | | | | | | | |
|  | | | | | | | | | | | 1 |  | AT |  | 28/02/2022 22:47 | |  |
| P1: [moderator]. In a typical village setting, anybody who dies has been bewitched. | | | | | | | | | | | | | | | |  |  |
|  | | | | | | | | | | | | | | | |  |  |
|  | | | | | | | | | | | | | | | | | |
| Reports\\Coding Summary By Code Report | | | | | | | | | Page 194 of 409 | | | | | | | | |
| 02/03/2022 02:43 | | | | | | | | | | | | | | | | | |
|  | | **Aggregate** |  | **Classification** |  | **Coverage** |  | **Number Of Coding References** | |  | **Reference Number** |  | **Coded By Initials** |  | **Modified On** | |  |
|  | | | | | | | | | | | | | | | | | |
|  | | | | | | | | | | | 2 |  | AT |  | 28/02/2022 22:48 | |  |
| M: Hmm, exactly. And here you are telling them that you want to—  P1: I'll tell you something; my Dad died of liver-related disease. My uncle also died of liver-related disease; I am ...<cuss word deleted> scared of that and I'm actually taking tests because I know—I believe in genetics. But of all those 2 people, none of them according to the villagers [died of liver, died of something else] ...<unintelligible> The other uncle—the other guy who never liked them and that was...<unintelligible>. So when you are bringing that, the reason why I laughed, we are living in a totally different setup from maybe Europe or United States. We are living in a setup where you've died because of some other causes rather than illness even when you have clearly died of this illness. So you see it really depends on the individual who has it, because you are not going to give—even the elder you have picked may never believe— | | | | | | | | | | | | | | | |  |  |
|  |  |  |  |  |  |  |  |  |  |  |  |  |  |  |  |  |  |
|  | | | | | | | | | | | | | | | |  |  |
| **Nodes\\Community engagement\Understanding Community values, cultural norms and beliefs\Religious beliefs towards emerging technologies\Islamic faith beliefs** | | | | | | | | | | | | | | | |  |  |
|  | **Document** | | | | | | | | | | | | | | | |  |
|  | | **Files\\FGD for CAB #5_5_Transcript** | | | | | | | | | | | | | | |  |
|  |  | No |  |  |  | 0.0451 |  | 2 | |  | | | | | | |  |
|  | |  |  |  |  |  |  |  | |  | | | | | | | |
|  | | | | | | | | | | | 1 |  | AT |  | 01/03/2022 09:06 | |  |
| So I wanted to ask, what do you hear about technology in the communities? Do people believe in technology or not? P6.  P6: Technology especially on the side of Muslims; they don’t believe in that because they are taught different doctrines that say, "If you refuse to believe the prophet, you are condemned". An example is, there are some people I advised to go for the paternity test. Sometime back when the Muslim leader was around, there is a lady who had a child and two men claimed paternity of the child. So during that time, they asked, "Who is this woman's husband?" and they said, "It is him" and "Who is the owner of the bed?" "It is him". So he said, "I want to let you know that the owner of this bed is the father of the child". Now that is how the judgement is made in Islam— | | | | | | | | | | | | | | | |  |  |
|  | | | | | | | | | | | | | | | |  |  |
|  | | | | | | | | | | | 2 |  | AT |  | 01/03/2022 09:06 | |  |
| So I said, "Ahh! You could go to the DNA and find out the father of the child" and he said, "You are disagreeing with the prophet!". So I insisted and said, "Religion exists and back in the day, the final verdict of the prophet is what you would go by. But the world is evolving" and there is a word in Quran that says, "Consult others about what you do not know" <Speaks Arabic>. If you fall sick of malaria, you cannot see the germ itself but you go to an expert who will make the diagnosis and tell you, "You are suffering from malaria". Therefore, we should try to accept that there are those who know what we don’t know. Therefore—especially in the Muslim society, they are so rigid about accepting technology because a Muslim might not pick a phone call...<unintelligible> they exist in society. In this day and age, a mobile phone is necessary, it is a need and they just say, "Don’t tell me that!" So we still have that challenge especially among Muslims; ignorance is still high. Therefore, telling people in the community about technology requires thorough teaching in order for them to understand. Also giving examples for instance last evening, I was watching TV with some little girls, and they were talking about HIV/AIDS; they had never seen the situation before. Now when they saw the documentary about HIV/AIDS they were so shocked! "HIV/AIDS really affected these people!" They were frightened. I told them, "This is how they were, but even now that we have medication, one should still stay safe"—there was a gentleman who gave his story, he had HIV for about 30years but he is healthy. He said, "Even if I am still healthy, this disease has still affected me" and it is still evident that he is infected. There is a young adult who said he got HIV because he was promiscuous, and one girl said...<unintelligible>. So people really need to be aware and taught that technology actually exists. | | | | | | | | | | | | | | | |  |  |
|  |  |  |  |  |  |  |  |  |  |  |  |  |  |  |  |  |  |
|  |  |  |  |  |  |  |  |  |  |  |  |  |  |  |  |  |  |
|  | | | | | | | | | | | | | | | |  |  |
|  | | | | | | | | | | | | | | | | | |
|  | | | | | | | | | | | | | | | | | |
| Reports\\Coding Summary By Code Report | | | | | | | | | Page 195 of 409 | | | | | | | | |
| 02/03/2022 02:43 | | | | | | | | | | | | | | | | | |
|  | | **Aggregate** |  | **Classification** |  | **Coverage** |  | **Number Of Coding References** | |  | **Reference Number** |  | **Coded By Initials** |  | **Modified On** | |  |
| **Nodes\\Community engagement\Understanding Community values, cultural norms and beliefs\some people dont believe in science** | | | | | | | | | | | | | | | |  |  |
|  | **Document** | | | | | | | | | | | | | | | |  |
|  | | **Files\\FGD for CAB #5_5_Transcript** | | | | | | | | | | | | | | |  |
|  |  | No |  |  |  | 0.0519 |  | 1 | |  | | | | | | |  |
|  | |  |  |  |  |  |  |  | |  | | | | | | | |
|  | | | | | | | | | | | 1 |  | AT |  | 01/03/2022 09:08 | |  |
| Indeed. Because—let me first start with the instance of the religious person who was talking the client. Because if you look at the bibles we read, they have editions; having editions means every generation has something different. And the bible says something--a certain leader used it and said, "You will have different children that will change the narrative of their community" you know this person; if I make the statement, it will sound political yet we are in research right now—  <laughs>  Do you understand? And the bible itself says, "we have to be as wise as serpents" it means our brains are always active and that is why we come up with different innovations. So we have different references to show those people that it is not all about the bible, even now as a human, you could be a moving bible because you are the one who puts the bible's instructions into practice. Because it would be in vain if you don’t put it into practice. So I personally think, some people don’t believe in science; some do and others don’t. An example; there are some churches I know that believe HIV is cured through the bible; they pray for it and it is cured. The more one goes to church, the more they deteriorate but when he/she goes home, "My pastor prays!" but the one telling him that he would get cured, is taking his pills. Do you get me? We also have some people who don’t wish others well in religion. So I don’t say it in bad faith, but I give the scenario; but ever since people discovered the truth—you find that one goes to church from Monday to Sunday, he has bible studies but knows that he has to take his medicine at 10pm in order to survive. So this person says, "I shall pray and plead the blood of Jesus, but continue taking my medicine" which means people understand science. You see even the elderly are on tiktok! How about us in research. So I think we should just continue to assure them and show them the advantages, benefits of technology because in this day and age, if an elderly person is able to hold a phone worth shs. 300,000 but another person says, "I don’t need it!" The old person even buys data and takes selfies; you understand? Which means we need to show people that every day that goes by, you cannot remain the same; every month and day that goes by—if you were 2years old—I started by crawling but now I can walk, run, speak, speak to those older than me and even those who have just been born. Which means there is always a new update, and technology is always improving...<unintelligible>Before my mother passed away, I was amazed and asked her, "Mother, don’t you think to yourself how you are in Uganda but you are able to communicate with your brother in America? How do you think things move on?" and she said, "We expected this; actually the clothes and shoes you wear now, are the ones we used to have in the days of the 'twist dance', the old trends like checked clothes, bell bottoms, are just coming back in this current generation".  So I think the more we sensitize people about the benefits, the more they will comprehend and adapt to it, then move on with it. | | | | | | | | | | | | | | | |  |  |
|  |  |  |  |  |  |  |  |  |  |  |  |  |  |  |  |  |  |
|  |  |  |  |  |  |  |  |  |  |  |  |  |  |  |  |  |  |
|  |  |  |  |  |  |  |  |  |  |  |  |  |  |  |  |  |  |
|  | | | | | | | | | | | | | | | |  |  |
