# Supplementary material for: The role of community engagement in promoting research participants’ understanding of pharmacogenomic research results: Perspectives of stakeholders involved in HIV/AIDS research and treatment
Source: PLoS One. 2024 Apr 2;19(4):e0299081. doi: 10.1371/journal.pone.0299081 (PMC10986979; doi:10.1371/journal.pone.0299081)
Supplement: S2 Dataset — (DOCX) [file pone.0299081.s002.docx]

| \| Coding Summary By Code \| \| --- \| \| THE ROLE OF COMMUNITY ENGAGEMENT IN PROMOTING UNDERSTANDING OF INDIVIDUAL PHARMACOGENOMICS RESEARCH RESULTS..  ***REC members’ perspectives*** \| | | | | | | | | | | | | | | | | | |
| --- | --- | --- | --- | --- | --- | --- | --- | --- | --- | --- | --- | --- | --- | --- | --- | --- | --- | --- | --- |
| 08/07/2022 10:27 | | | | | | | | | | | | | | | | | |
|  | **Aggregate** | | |  | **Classification** |  | **Coverage** |  | **Number Of Coding References** | |  | **Reference Number** |  | **Coded By Initials** |  | **Modified On** |  |
| **Nodes\\Role of community engagement in returning results\helps community members understand pharmacogenetic research better** | | | | | | | | | | | | | | | | |  |
|  | |  | | | | | | | | | | | | | | |  |
|  | |  | | | | | | | | | | | | | | |  |
|  | | | | | | | | | | | | 10 |  | AT |  | 06/07/2022 15:44 |  |
|  | | what should be the role of community engagement in the process of returning these results?  R: Uhm, community involvement. Yes uh, the role is that we need to involve the community because these people are from our communities there and when they go back, they talk a lot with their neighbors, those who are not even participants. So there's a lot we need to involve the community with because also the community—different communities have different environments which may affect the study. So we need to also involve them, "Where do you come from? How do you—". So it is really important that we need to also involve them as a community, not putting them all in the study but so that you know about the background of the community, what kind of people live there, are they going to drugs and things? Are they fishermen? Those kinds of things. | | | | | | | | | | | | | | |  |
|  | |  |  |  |  |  |  |  |  |  |  |  |  |  |  |  |  |
|  | |  | | | | | | | | | | | | | | |  |
|  | | | | | | | | | | | | | | | | | |
| Reports\\Coding Summary By Code Report | | | | | | | | | | Page 4 of 270 | | | | | | | |
| 08/07/2022 10:27 | | | | | | | | | | | | | | | | | |
|  | **Aggregate** | | |  | **Classification** |  | **Coverage** |  | **Number Of Coding References** | |  | **Reference Number** |  | **Coded By Initials** |  | **Modified On** |  |
|  | | | | | | | | | | | | | | | | | |
|  | | | | | | | | | | | | 11 |  | AT |  | 06/07/2022 15:47 |  |
|  | | **Nodes\\Role of community engagement in returning results\building trust**    R: Hmmm. That is a difficult one; you know people are not easy to really trust you fully <laughs> whatever you tell them, [Yes! to trust you fully. The COVID vaccine is enough] yeah! that's true. So they can never trust you fully but you can build a strong confidence in them—in you, by telling them; being open. Me I believe in being open. You know our communities are very interesting; the way you talk to them, if they see openness in you and you tell them, "You know this is true because we have heard it happen somewhere", they start trusting you more that 'eh, this one is very open and I can trust him'. So I believe in being more open; you know they always believe that research we are just looking for things, we don’t—you know, they don't benefit a lot from it because we are looking for what we are looking for and when we finish, that is it. [Hmmm, hmmm] But you have to keep building confidence in them by being open, by telling them—giving them a lot of knowledge even if it’s not part of the research. Just gather them together and teach them something that is nothing to do with research and then slowly, slowly, you put in your own research things and they become very, very trustworthy; they'll give you a lot of details. | | | | | | | | | | | | | | |  |
|  | |  |  |  |  |  |  |  |  |  |  |  |  |  |  |  |  |
|  | |  | | | | | | | | | | | | | | |  |
|  | |  | | | | | | | | | | | | | | |  |
|  | |  |  |  |  |  |  |  |  |  |  |  |  |  |  |  |  |
|  | |  | | | | | | | | | | | | | | |  |
|  | | | | | | | | | | | | 5 |  | AT |  | 07/07/2022 14:40 |  |
|  | |  | | | | | | | | | | | | | | |  |
|  | | | | | | | | | | | | | | | | | |
|  | | | | | | | | | | | | | | | | | |
| Reports\\Coding Summary By Code Report | | | | | | | | | | Page 16 of 270 | | | | | | | |
| 08/07/2022 10:27 | | | | | | | | | | | | | | | | | |
|  | **Aggregate** | | |  | **Classification** |  | **Coverage** |  | **Number Of Coding References** | |  | **Reference Number** |  | **Coded By Initials** |  | **Modified On** |  |
|  | | | | | | | | | | | | | | | | | |
|  | | | | | | | | | | | | 6 |  | AT |  | 07/07/2022 14:45 |  |
|  | | So I'm just wondering, what could be the role of engaging community, regarding genomic studies and genomics results in your opinion?  R: Uh, in my opinion—let me see. So the community definitely...<unintelligible> people we live with on a daily basis. So it depends on what kind of results we are getting back. Uhm, definitely if the study you're working on involves genomics, the community needs to be informed right from the word go, they need to understand what you're doing exactly. And also, they'll guide you on how sensitive that information is in their community, and even the perhaps how it should be delivered in case you have to deliver it, you know. So I think they are very important, especially if the results are going to reflect on that community, not just as an individual, but if they are going to find out that people of this community or this tribe or this region, you know, they have this genomic predisposition. Now that is going to be very key in engaging them right from the word go, [Yeah] for them to understand what you want to do and why you're doing it and to get their mind. And also, before they even get the results back....<unintelligible> and agree with them what kind of results are you going to bring back, and how are you going to bring them back and who are you going to give them to—if it's going to be general. But if it’s going to be at individual level, then I think we need to go through what we said earlier on; let this individual know about the possible implications of the results you're going to give them back, and be sure if they want them back and what type of results they want back in case they are there. And also in case you're not giving them any results, what does it mean, because also not telling me—in case you keep quiet, what do you mean? Because even when you keep quiet, you are communicating sometimes. So all those need to be agreed upon and leave no place for ambiguity. [Yeah] Uh, yes. But for results...<unintelligible> they may have an impact on the community, we must engage the community members and especially the community leaders to know what you're doing, and also what kind of results you're likely to get and how it may impact that community, and how it can be delivered back to them if it’s at all to be delivered back to them. Yes, even if to guide you; you don’t think for them in that case, you have to discuss with them and they guide you and you follow their lead in that case. | | | | | | | | | | | | | | |  |
|  | |  |  |  |  |  |  |  |  |  |  |  |  |  |  |  |  |
|  | |  |  |  |  |  |  |  |  |  |  |  |  |  |  |  |  |
|  | |  | | | | | | | | | | | | | | |  |
|  | | | | | | | | | | | | 7 |  | AT |  | 07/07/2022 14:53 |  |
|  | | I: Yes, coming from the participant, and uhh—yes, coming from the participants and the findings from this particular one individual involved in this study and—  R: And it may affect the whole family;  I: Yes, and it may affect the whole family and we want to understand, should these results be informed to family members? and how should it be done?  R: This individual you begun with, that's where it starts; that's where it starts. Now after the individual, first of all, they don't want to get back the results or not? Once you clear that, okay you want to get the results; what kind of results? we went through those different choices whether the results are negative, are neutral, are positive, whichever it is and you agree on what they want to get back. Now I'm just thinking of a scenario, they've agreed to get whatever it is; everything! I want to know everything about me. So then at that level--for them as an individual. Uh, now...<unintelligible> now going to inform their family, this individual needs to also consent to that I think; they need to consent to this—this information going now to the rest of the family members. And—but also when it gets to the family members, I think they also need to have a right to know or not to know. [Oh yeah] That's what I think about; they have a right to know or not to know, so that we don't impose to them information they didn't uh— [they didn’t request for] request for, yes, and it may impact them negatively. [Hmmm] So whatever can be done to get their consent to receive this information or not, I think that would be good to know, yes, and understand whether they really want to know the results. And I think you also have to take them through kind of what we did at the individual level; what kind of results and what kind of response they would say depending on whether the results the results are positive, neutral or negative. But I think I would imagine it would be important for them to know whether they want to get those results or not. If they don't want, then I don't think it would be right and ethical to impose on them results they don't want to know. | | | | | | | | | | | | | | |  |
|  | |  |  |  |  |  |  |  |  |  |  |  |  |  |  |  |  |
|  | |  |  |  |  |  |  |  |  |  |  |  |  |  |  |  |  |
|  | |  | | | | | | | | | | | | | | |  |
|  | | | | | | | | | | | | 5 |  | AT |  | 07/07/2022 18:34 |  |
|  | | So what could be the role of REC members in the process of returning results to participants?  R: The REC members do not have a role in the process of returning results. The REC members should be able to review and approve or disapprove if the document is not uh—if the method, the methodology is not clearly described. [Hmmm] Uhm, so the REC members' responsibility will be in areas where things are not going well within the study and then they are either whistleblowers, or something is happening which is not supposed to happen. For example, if somebody is returning results and he is saying, "You know, people are here doing this study, then they have told me I have this very grave condition which is going to take me up and then you are just going to die or something". And then eventually if the REC receives it, usually of course that calls for a review or monitoring of such a study—that is actually where the REC comes in. [Okay, Okay] Otherwise after the approval, the process continues and it is now the responsibility of the investigator to take on what is happening. | | | | | | | | | | | | | | |  |
|  | |  |  |  |  |  |  |  |  |  |  |  |  |  |  |  |  |
|  | |  | | | | | | | | | | | | | | |  |
|  | | | | | | | | | | | | 6 |  | AT |  | 07/07/2022 18:35 |  |
|  | | So what could be the role of REC members in the process of returning results to participants?  R: The REC members do not have a role in the process of returning results. The REC members should be able to review and approve or disapprove if the document is not uh—if the method, the methodology is not clearly described. [Hmmm] Uhm, so the REC members' responsibility will be in areas where things are not going well within the study and then they are either whistleblowers, or something is happening which is not supposed to happen. For example, if somebody is returning results and he is saying, "You know, people are here doing this study, then they have told me I have this very grave condition which is going to take me up and then you are just going to die or something". And then eventually if the REC receives it, usually of course that calls for a review or monitoring of such a study—that is actually where the REC comes in. [Okay, Okay] Otherwise after the approval, the process continues and it is now the responsibility of the investigator to take on what is happening. | | | | | | | | | | | | | | |  |
|  | |  |  |  |  |  |  |  |  |  |  |  |  |  |  |  |  |
|  | |  | | | | | | | | | | | | | | |  |
|  | | | | | | | | | | | | 7 |  | AT |  | 07/07/2022 18:37 |  |
|  | | **Nodes\\Role of community engagement in returning results\communicate PG results at community level**  I: Okay. And that brings me to a question of uhm, how well should researchers communicate these results at community level? That these results are accepted, they are not misunderstood, the misconceptions are rolled out a bit.  R: Trust is built right from the—from the, again, inception of the study. And then the presentation of the study, the review of that particular study and how the communication between the person and the researcher and the person who is going to participate. The way you communicate is the way people will understand you and making sure that you actually continue doing exactly what you plan to do other than changing goal posts and you start doing something else [laughs] which was not there. | | | | | | | | | | | | | | |  |
|  | |  | | | | | | | | | | | | | | |  |
| Reports\\Coding Summary By Code Report | | | | | | | | | | Page 18 of 270 | | | | | | | |
| 08/07/2022 10:27 | | | | | | | | | | | | | | | | | |
|  | **Aggregate** | | |  | **Classification** |  | **Coverage** |  | **Number Of Coding References** | |  | **Reference Number** |  | **Coded By Initials** |  | **Modified On** |  |
|  | | | | | | | | | | | | | | | | | |
|  | | | | | | | | | | | | 8 |  | AT |  | 07/07/2022 18:38 |  |
|  | | Do you think it’s important that that result is also communicated to another party beyond the participant?  R: Again that is uh—how do you start? I think it is basically the very person who participated in the study who should be engaged or who should [give permission to] yeah, be encouraged to share the results. And then if the person says yes uh, "My people, my children, this is what has been seen and so we are having this kind of thing"—because then it is going to bring a lot of anxiety in other people who never participated in this study before. [Who never participated] So I think that should only pass through the participant. | | | | | | | | | | | | | | |  |
|  | |  | | | | | | | | | | | | | | |  |
|  | | | | | | | | | | | | 9 |  | AT |  | 07/07/2022 18:39 |  |
|  | | So they feel like they would want to convey this information to people, to their family members and then they go for testing, but they have that fear that they may think this one is just saying things because they are kind of mad. So how would we try to help that kind of person?  R: It is still about communication; the person needs to understand the importance. If the person eventually engages his or her team and say you know, 'this is what has happened' and he thinks that that community will not understand, then he should also discuss, "Can I bring these people to talk to you about these findings?" If they say yes and everybody agrees, then somebody can speak to them. But it must be through the participant. | | | | | | | | | | | | | | |  |
|  | |  | | | | | | | | | | | | | | |  |
|  | | | | | | | | | | | | 10 |  | AT |  | 07/07/2022 18:41 |  |
|  | | I: Okay, so we've also talked about how the same will be approached. And lastly, I will just want to ask whether you've heard about genetic counsellors and what their role could be.  R: Genetic counsellors in terms of—like in research?  I: Yes, in research or even the next steps after the results have been conveyed or even during the whole process of the research study?  R: So what you are saying here is that a research that is doing genetic studies could engage geneticists or counsellors? It is okay still, for them to talk about it but I'm not sure they should really come at the very beginning [Okay]. I mean uh, first of all you even don't know what your— [your findings are going to be, yeah] findings will be, exactly! So I think the counsellor should—they can get engaged but maybe later on because when you are doing the research, you don't know what you are going to find. [Hmmm, okay] Uhm yeah. So they can be engaged but probably later. | | | | | | | | | | | | | | |  |
|  | |  |  |  |  |  |  |  |  |  |  |  |  |  |  |  |  |
|  | |  | | | | | | | | | | | | | | |  |
|  | **Files\\KII_Male_REC Participant #9_Transcript** | | | | | | | | | | | | | | | |  |
|  | |  | | | | | | | | | | | | | | |  |
|  | |  | | | | | | | | | | | | | | |  |
|  | | | | | | | | | | | | 2 |  | AT |  | 08/07/2022 08:37 |  |
|  | | R: Uhm, I think—I think the safety or the interest of the participant should be taken care of at all times during the research process, and so for me here it’s about safety; protection of the participants. So if the information identified from the pharmacogenetic analysis of the genes or the enzyme systems in the body indicate a certain condition, in a judgement should be made whether this condition is—is—is significant enough to affect the general health of the person, moving forward or not. I certainly don't think any incidental finding—maybe to agree on the definition of incidental finding would have been good but I think the genetic findings that should be given at any cost to the participant is those that have implication on their health or general wellbeing. Because there is so much information in our genetic code so some are redundant, they don't have the implications. So I think really it’s not necessary to give back uh—uh, you know, to give back any information that you find. However, this should be made clear to the participants at consenting as to which specific kind of results will be given. Of course participants will want—in some cases or in many cases, their results to be given to them, so I think it’s the duty of the researcher to define clearly and the REC to define clearly what boundaries of genetic results that are incidental in nature should be given back to the participants. Yeah. | | | | | | | | | | | | | | |  |
|  | |  |  |  |  |  |  |  |  |  |  |  |  |  |  |  |  |
|  | |  | | | | | | | | | | | | | | |  |
| Reports\\Coding Summary By Code Report | | | | | | | | | | Page 19 of 270 | | | | | | | |
| 08/07/2022 10:27 | | | | | | | | | | | | | | | | | |
|  | **Aggregate** | | |  | **Classification** |  | **Coverage** |  | **Number Of Coding References** | |  | **Reference Number** |  | **Coded By Initials** |  | **Modified On** |  |
|  | | | | | | | | | | | | | | | | | |
|  | | | | | | | | | | | | 3 |  | AT |  | 07/07/2022 19:00 |  |
|  | | **Nodes\\Role of community engagement in returning results\Role of REC members**  What could be REC member's role in the process of feedback or returning these results to participants?  R: Uhm, wow! Of course, perhaps one is to—to be able to uhm, stipulate during the review process that certain results should be given to the participants as like the starting point, [Aha! Hmmm] and then subsequently maybe through uh routine monitoring of the studies—uh routine monitoring of such studies to ensure that all REC recommendations are being adhered to during the conduct of the study. The REC’s primary role is to protect participants and their communities from research related harm. As you know a lot of harm can happen for example we need to ensure that the information given to them is simple, clear and easy to understand because the results can be misinterpreted and cause psychological harm to participants. | | | | | | | | | | | | | | |  |
|  | |  | | | | | | | | | | | | | | |  |
|  | | | | | | | | | | | | 4 |  | AT |  | 07/07/2022 19:02 |  |
|  | | But at what point do you think  R: RECs have a role of reviewing and advising investigators how to design informed consent processes and how these results can be safely returned. We can achieve this by standardizing genetics protocol templates for genetics and genomic studies. Many researchers don’t even know what ethical issues that might arise when conducting such research. So these templates will be very helpful to them | | | | | | | | | | | | | | |  |
|  | |  |  |  |  |  |  |  |  |  |  |  |  |  |  |  |  |
|  | |  | | | | | | | | | | | | | | |  |
|  | | | | | | | | | | | | 5 |  | AT |  | 08/07/2022 08:37 |  |
|  | | | | | | | | | | | | | | | | | |
| Reports\\Coding Summary By Code Report | | | | | | | | | | Page 20 of 270 | | | | | | | |
| 08/07/2022 10:27 | | | | | | | | | | | | | | | | | |
|  | **Aggregate** | | |  | **Classification** |  | **Coverage** |  | **Number Of Coding References** | |  | **Reference Number** |  | **Coded By Initials** |  | **Modified On** |  |
|  | | | | | | | | | | | | | | | | | |
|  | | | | | | | | | | | | 9 |  | AT |  | 07/07/2022 19:05 |  |
|  | | Now like the CAB members you mentioned, because they are the ones you felt are suitable to return the results, and what should be their role.  R: Uhm, the—I think the Community Advisory Board or community engagement in this case is to—is to help avoid uh miss-interpretation of the results, [Okay, Hmmm] you know, if not well-communicated then the results will be totally misinterpreted and the implications of that can be there. [Yes] So the Community Advisory Board or community engagement should help in uh awareness creation; creating awareness among community members of the need to know the uniqueness of each one of them and that uniqueness is not in any way uh making you less of a person but just makes you a unique being. So that people can take up these results and don't get psychological stress from them and get any other health complications from getting to know their genetic uh results that may be different from the other people. | | | | | | | | | | | | | | |  |
|  | |  |  |  |  |  |  |  |  |  |  |  |  |  |  |  |  |
|  | |  | | | | | | | | | | | | | | |  |
|  | |  | | | | | | | | | | | | | | |  |
|  | **Files\\KII_Male_REC Participant #9_Transcript** | | | | | | | | | | | | | | | |  |
|  | Yes | | |  |  |  | 0.1235 |  | 3 | |  | | | | | |  |
|  |  | | |  |  |  |  |  |  | |  | | | | | | |
|  | | | | | | | | | | | | 1 |  | AT |  | 07/07/2022 19:04 |  |
|  | | So who do you think in your opinion would be the most suitable person to communicate this information to the participant?  R: I think the CAB members; Community Advisory Board members [Okay] uh would be in position because this information needs to be broken down to the basic level that can be understood by the participants. So the Community Advisory Board members I feel would be the most appropriate persons to give these results back to the individuals. | | | | | | | | | | | | | | |  |
|  | |  | | | | | | | | | | | | | | |  |
|  | | | | | | | | | | | | 2 |  | AT |  | 07/07/2022 19:04 |  |
|  | |  | | | | | | | | | | | | | | |  |
|  | | | | | | | | | | | | 3 |  | AT |  | 07/07/2022 19:05 |  |
|  | | **Nodes\\Role of community engagement in returning results\helps community members understand pharmacogenetic research better**  Now like the CAB members you mentioned, because they are the ones you felt are suitable to return the results, and what should be their role.  R: Uhm, the—I think the Community Advisory Board or community engagement in this case is to—is to help avoid uh miss-interpretation of the results, [Okay, Hmmm] you know, if not well-communicated then the results will be totally misinterpreted and the implications of that can be there. [Yes] So the Community Advisory Board or community engagement should help in uh awareness creation; creating awareness among community members of the need to know the uniqueness of each one of them and that uniqueness is not in any way uh making you less of a person but just makes you a unique being. So that people can take up these results and don't get psychological stress from them and get any other health complications from getting to know their genetic uh results that may be different from the other people. | | | | | | | | | | | | | | |  |
|  | |  |  |  |  |  |  |  |  |  |  |  |  |  |  |  |  |
|  | |  | | | | | | | | | | | | | | |  |
|  | | **Nodes\\Approaches or strategies for safe return of individual results\Personnel to communicate results\CAB\genetic counsellors** | | | | | | | | | | | | | | |  |
|  | | | **Document** | | | | | | | | | | | | | |  |
|  | **Files\\KII_Male_REC Participant #9_Transcript** | | | | | | | | | | | | | | | |  |
|  | No | | |  |  |  | 0.0750 |  | 2 | |  | | | | | |  |
|  |  | | |  |  |  |  |  |  | |  | | | | | | |
|  | | | | | | | | | | | | 1 |  | AT |  | 07/07/2022 19:04 |  |
|  | | So who do you think in your opinion would be the most suitable person to communicate this information to the participant?  R: I think the CAB members; Community Advisory Board members [Okay] uh would be in position because this information needs to be broken down to the basic level that can be understood by the participants. So the Community Advisory Board members I feel would be the most appropriate persons to give these results back to the individuals.  **Files\\KII_Female_REC Participant #2_Transcript**  R: Sharing this genetics information with community members can sometimes be tricky. Many of them are illiterate and do not know much about genes. Therefore, the research team needs to be very creative and probably work with genetic counsellors who understand both the scientific information and societal norms. | | | | | | | | | | | | | | |  |
|  | |  | | | | | | | | | | | | | | |  |
|  | | | | | | | | | | | | | | | | | |
|  | | | | | | | | | | | | | | | | | |
| Reports\\Coding Summary By Code Report | | | | | | | | | | Page 65 of 270 | | | | | | | |
| 08/07/2022 10:27 | | | | | | | | | | | | | | | | | |
|  | **Aggregate** | | |  | **Classification** |  | **Coverage** |  | **Number Of Coding References** | |  | **Reference Number** |  | **Coded By Initials** |  | **Modified On** |  |
|  | | | | | | | | | | | | | | | | | |
|  | | | | | | | | | | | | 2 |  | AT |  | 07/07/2022 19:05 |  |
|  | | Now like the CAB members you mentioned, because they are the ones you felt are suitable to return the results, and what should be their role.  R: Uhm, the—I think the Community Advisory Board or community engagement in this case is to—is to help avoid uh miss-interpretation of the results, [Okay, Hmmm] you know, if not well-communicated then the results will be totally misinterpreted and the implications of that can be there. [Yes] So the Community Advisory Board or community engagement should help in uh awareness creation; creating awareness among community members of the need to know the uniqueness of each one of them and that uniqueness is not in any way uh making you less of a person but just makes you a unique being. So that people can take up these results and don't get psychological stress from them and get any other health complications from getting to know their genetic uh results that may be different from the other people. | | | | | | | | | | | | | | |  |
|  | |  |  |  |  |  |  |  |  |  |  |  |  |  |  |  |  |
|  | |  | | | | | | | | | | | | | | |  |
|  | | **Nodes\\Approaches or strategies for safe return of individual results\Personnel to communicate results\community social worker** | | | | | | | | | | | | | | |  |
|  | | | **Document** | | | | | | | | | | | | | |  |
|  | **Files\\KII_Male_REC Participant #9_Transcript** | | | | | | | | | | | | | | | |  |
|  | No | | |  |  |  | 0.0484 |  | 1 | |  | | | | | |  |
|  |  | | |  |  |  |  |  |  | |  | | | | | | |
|  | | | | | | | | | | | | 1 |  | AT |  | 07/07/2022 19:04 |  |
|  | | I: Yeah, I see. And then, uh, what kind of mode of communication—actually the channel of communication would be very appropriate? Does it really have to be a face-to-face? Can we do some phone conversations, can we do some kind of emails especially for participants who may be very literate? In your opinion.  R: Hmmm. Well the information first has to be confidential, uh, so the mode of the information transfer should ensure confidentiality of the individual. Uh, so I think uh—and see it has to be explained well; it’s not just a matter of sending an email or making a phone call, there has to be some bit of explanation for someone to understand. So I think face-to-face would be the most appropriate for me, for delivering these results and you know, community social worker or social worker in the Community advisory board team should be able to communicate these results, face-to-face to the patients. | | | | | | | | | | | | | | |  |
|  | |  |  |  |  |  |  |  |  |  |  |  |  |  |  |  |  |
|  | |  | | | | | | | | | | | | | | |  |
