# Supplementary material for: The role of community engagement in promoting research participants’ understanding of pharmacogenomic research results: Perspectives of stakeholders involved in HIV/AIDS research and treatment
Source: PLoS One. 2024 Apr 2;19(4):e0299081. doi: 10.1371/journal.pone.0299081 (PMC10986979; doi:10.1371/journal.pone.0299081)
Supplement: S3 Dataset — (DOCX) [file pone.0299081.s003.docx]

| 01/12/2021 00:27 | | | | | | | | | | | | | | | | | |
| --- | --- | --- | --- | --- | --- | --- | --- | --- | --- | --- | --- | --- | --- | --- | --- | --- | --- |
| Coding Summary By Code | | | | | | | | | | | | | | | | | |
| THE ROLE OF COMMUNITY ENGAGEMENT IN PROMOTING UNDERSTANDING OF INDIVIDUAL PHARMACOGENOMICS RESEARCH RESULTS..  ***Researchers perspectives*** | | | | | | | | | | | | | | | | | |
| 01/12/2021 00:27 | | | | | | | | | | | | | | | | | |
|  | | | **Aggregate** |  | **Classification** |  | **Coverage** |  | **Number Of Coding References** | |  | **Reference Number** |  | **Coded By Initials** |  | **Modified On** |  |
| **Node\\community engagement\\the role of community representatives\Community Advisory Boards** | | | | | | | | | | | | | | | | |  |
|  | | | **Aggregate** |  | **Classification** |  | **Coverage** |  | **Number Of Coding References** | |  | **Reference Number** |  | **Coded By Initials** |  | **Modified On** |  |
|  | | | | | | | | | | | | | | | | | |
|  | R: Please capture that. If you're disseminating research about something, in fact if it is out of 5—of course the participants must be there, and then other stakeholders like the Ministry because if they can change policy. And then maybe the CAB; the Community Advisory Board because they are the ones we link Community with the researchers. And then the actual researchers who may be funded the study or did everything, all those other things. And then most importantly, the sites where the studies were done; they should know that when they were in Kisenyi, a study they did there changed Policy in the world. | | | | | | | | | | | | | | | | |
|  | R: Yeah, uh. When you explain in terms of simplification of the message, lay people or maybe—you could have maybe community volunteers or people who can better simplify this message so that the participants can receive it better. So yes, it would be important actually to involve them. | | | | | | | | | | | | | | | | |
|  | | | | | | | | | | | | 17 |  | AT |  | 30/11/2021 09:49 |  |
|  | And then you can be sensitive to your community, you know the things that are our issues here. In the West, they might be people who don’t want to know if they are going to get cancer or not because they can do something about it. | | | | | | | | | | | | | | | | |
|  |  | | | | | | | | | | | | | | | | |
|  | R: Uh, Yeah. So the people in the community; the leaders and the peers will give you the context that you are saying. They will give you the context, they'll give you words for genetics, they'll tell you what is taboo [Hmm] for your setting. Like I said, even within a country Uganda, in one tribe genetics can be seen as something that is predicting your future or your past and it might be taboo [yes] to predict the future or look into your past. [Yes, hmm] So that can be taboo. So I think this understanding the context—the leaders—simple answer to...<unintelligible> I mean is community engagement necessary? Yes! Community engagement also helps you understand how your topic--disseminating results in a way that is culturally sensitive, gender, language sensitive. Yeah. | | | | | | | | | | | | | | | | |
|  |  | | | | | | | | | | | | | | | | |
|  | | | | | | | | | | | | 22 |  | AT |  | 30/11/2021 09:53 |  |
|  | Yes! Community engagement also helps you understand how your topic--disseminating results in a way that is culturally sensitive, gender, language sensitive. Yeah. | | | | | | | | | | | | | | | | |
|  |  | | | | | | | | | | | | | | | | |
|  | | | | | | | | | | | | 4 |  | AT |  | 30/11/2021 13:50 |  |
|  | R: Yes, absolutely. But Uhm you need to be careful, you need to study your audience—the kind of participants you are dealing with. If you are dealing with young adults you might want to use maybe social media to engage, if you're dealing with you know, pregnant women, you might need to find groups. But community involvement at whatever level—I don’t know whether social media is part of it but at whatever level, it’s extremely important. | | | | | | | | | | | | | | | | |
|  |  | | | | | | | | | | | | | | | | |
|  | | | | | | | | | | | | 5 |  | AT |  | 30/11/2021 13:50 |  |
|  | It may be hard for you to penetrate certain groups unless you are using members of their community or people that they know, they trust, leaders and what not, CAB members. Yeah, that is extremely important. | | | | | | | | | | | | | | | | |
|  |  | | | | | | | | | | | | | | | | |
| Reports\\Coding Summary By Code Report | | | | | | | | | | Page 8 of 249 | | | | | | | |
| 01/12/2021 00:27 | | | | | | | | | | | | | | | | | |
|  | | | **Aggregate** |  | **Classification** |  | **Coverage** |  | **Number Of Coding References** | |  | **Reference Number** |  | **Coded By Initials** |  | **Modified On** |  |
|  | | | | | | | | | | | | | | | | | |
|  | | | | | | | | | | | | 6 |  | AT |  | 30/11/2021 13:52 |  |
|  | : I think it might depend on the kind of study. There are certain studies that are maybe—where participants will not engage so much with the study team. But if it is a kind of study for example like my study where participants are very, very much engaged with the study team, then that is absolutely the...to disseminate these results. They should be the ones that are involved with these community engagements and get feedback to the participants. Because these kind of interactions require trust, and trust is built by connection. So if there is no connection...<unintelligible> Say for example everything was by correspondence or it was mail, or it was phone calls and we never got to engage | | | | | | | | | | | | | | | | |
|  |  | | | | | | | | | | | | | | | | |
|  | | | | | | | | | | | | 7 |  | AT |  | 30/11/2021 13:52 |  |
|  | R: Yes. At community level there is a lot of benefit. [Hmm] Yes if we realized that anyway for us as Africans or as Ugandans or as this particular tribe, this seems to be something that applies to us. We are very prone to sickle cells for instance, because it’s of this common gene around us, and then people can say 'okay, maybe it is time we considered intermarrying'. Yes, at community level it can be very good. | | | | | | | | | | | | | | | | |
|  |  | | | | | | | | | | | | | | | | |
|  | | | | | | | | | | | | 6 |  | AT |  | 30/11/2021 12:29 |  |
|  | R: Yes. I'm thinking now community—we have communities of varying categories. One is our definition of community here; we are not going to go to collect the LC Council and reveals us. | | | | | | | | | | | | | | | | |
|  |  | | | | | | | | | | | | | | | | |
|  | | | | | | | | | | | | 7 |  | AT |  | 30/11/2021 12:30 |  |
|  | For me when I talk about community, if it is about ...we know that there is NDA for instance, that can receive this information on behalf of the people of the community and can take judgement on behalf of the community. [Hmm | | | | | | | | | | | | | | | | |
|  |  | | | | | | | | | | | | | | | | |
|  | | | | | | | | | | | | 8 |  | AT |  | 30/11/2021 12:30 |  |
|  | If it is something to do with cancer, with some group being more prone to cancer, the community to reveal it now will be Cancer Institute and the people involved. So that if they see someone of a particular grouping, they can look out more for what we are talking about. | | | | | | | | | | | | | | | | |
|  |  | | | | | | | | | | | | | | | | |
|  | | | | | | | | | | | | | | | | | |
|  | | | | | | | | | | | | | | | | | |
| Reports\\Coding Summary By Code Report | | | | | | | | | | Page 10 of 249 | | | | | | | |
| 01/12/2021 00:27 | | | | | | | | | | | | | | | | | |
|  | | | **Aggregate** |  | **Classification** |  | **Coverage** |  | **Number Of Coding References** | |  | **Reference Number** |  | **Coded By Initials** |  | **Modified On** |  |
|  | | | **Files\\KII_Male_Researcher_12_Transcript** | | | | | | | | | | | | | |  |
|  |  |  | Yes |  |  |  | 0.1856 |  | 15 | |  | | | | | |  |
|  | | |  |  |  |  |  |  |  | |  | | | | | | |
|  | | | | | | | | | | | | 1 |  | AT |  | 30/11/2021 18:46 |  |
|  |  | | | | | | | | | | | | | | | | |
|  | | | | | | | | | | | | 4 |  | AT |  | 30/11/2021 17:39 |  |
|  | But if you want to do one-on-one dissemination, it can also be a bit tricky. But then also, some participants might not want to appear as part of the group—may not want other participants to know 'I was in this study'. So that can be some of the complexities; how would you prefer to disseminate, what would the patient be interested in but again in that case, that would be part of the consenting process. You know, 'would you like?' because we always say in the protocol, the patient can request a copy of bla… bla...bla but usually that article is written in very scientific jargon and things that might not be very meaningful to a non-field person. And so the part of community engagement in a language that the patients understand after the ...<unintelligible>I don’t think that has been emphasized even by the ethics board. So it’s really up to the researchers; it’s up to you. | | | | | | | | | | | | | | | | |
|  |  |  |  |  |  |  |  |  |  |  |  |  |  |  |  |  |  |
|  |  | | | | | | | | | | | | | | | | |
|  | | | | | | | | | | | | 5 |  | AT |  | 30/11/2021 17:39 |  |
|  | : And usually after presenting at a conference or an abstract, is usually where we close the chapter on that. | | | | | | | | | | | | | | | | |
|  |  | | | | | | | | | | | | | | | | |
| Reports\\Coding Summary By Code Report | | | | | | | | | | Page 12 of 249 | | | | | | | |
| 01/12/2021 00:27 | | | | | | | | | | | | | | | | | |
|  | | | **Aggregate** |  | **Classification** |  | **Coverage** |  | **Number Of Coding References** | |  | **Reference Number** |  | **Coded By Initials** |  | **Modified On** |  |
|  | | | | | | | | | | | | | | | | | |
|  | | | | | | | | | | | | 6 |  | AT |  | 30/11/2021 17:41 |  |
|  | I think if it’s a long-term cooperation that can be viewed from the point of building trust with the community, you know. But that’s the way I think I'd angle it because usually—the sponsor is usually interested in the data, usually. When they have their data, they'd have their own objectives, their own interests. This other interest is a patient interest and it comes more from a patient-advocacy point of view. | | | | | | | | | | | | | | | | |
|  |  | | | | | | | | | | | | | | | | |
|  | | | | | | | | | | | | 7 |  | AT |  | 30/11/2021 17:42 |  |
|  | So it would have to be an ethics board or a National regulator that insists on this being part of the process, otherwise if we leave it to the sponsor, I don’t think there's much value they might see in that extra expense in that particular activity. Yeah. But some might be, some might not be so it might have to be just standardized. And then the patients can decide if they are interested and if they are interested in receiving results, which results and how would they like to receive the results. Do they mind applying in a group, or they want it to be one-on-one? And then from then, it can also inform future planning because that’s also a research study of its own; the preferences of the patients and all that. After a while you can get a sense of what this community would prefer. | | | | | | | | | | | | | | | | |
|  |  | | | | | | | | | | | | | | | | |
|  | | | | | | | | | | | | 8 |  | AT |  | 30/11/2021 17:42 |  |
|  | : So I thought it depends—So I think it depends. Uhm, you know it’s ...<unintelligible>in my community if uh—because during the study, the participants may not really, really see the P.I or you know. But then the trust might be built with the medical doctor, you get what I'm saying [Hmm], that might be the person they are interacting with quite a lot. [Hmm | | | | | | | | | | | | | | | | |
|  |  | | | | | | | | | | | | | | | | |
|  | | | | | | | | | | | | 9 |  | AT |  | 30/11/2021 17:43 |  |
|  | | | **Files\\KII_Male_Researcher_3_Transcript** | | | | | | | | | | | | | |  |
|  |  | | | | | | | | | | | | | | | | |
|  | | | | | | | | | | | | 4 |  | AT |  | 30/11/2021 18:03 |  |
|  | : So the need for community engagement to try and find some of these things—to try, kind of decide what will be communicated to the participants. [Hmm, hmm] there's much need for that. | | | | | | | | | | | | | | | | |
|  |  | | | | | | | | | | | | | | | | |
|  | | | | | | | | | | | | | | | | | |
| Reports\\Coding Summary By Code Report | | | | | | | | | | Page 13 of 249 | | | | | | | |
| 01/12/2021 00:27 | | | | | | | | | | | | | | | | | |
|  | | | **Aggregate** |  | **Classification** |  | **Coverage** |  | **Number Of Coding References** | |  | **Reference Number** |  | **Coded By Initials** |  | **Modified On** |  |
|  | | | | | | | | | | | | | | | | | |
|  | | | | | | | | | | | | 5 |  | AT |  | 30/11/2021 18:09 |  |
|  | : ...<inaudible>I think the same reason of empowering participants with knowledge, and of course the knowledge will eliminate the fears and misinformation...<inaudible> | | | | | | | | | | | | | | | | |
|  |  | | | | | | | | | | | | | | | | |
|  | | | **Files\\KII_Male_Researcher_6_Transcript** | | | | | | | | | | | | | |  |
|  |  | | | | | | | | | | | | | | | | |
|  |  | | | | | | | | | | | | | | | | |
|  | | | | | | | | | | | | 19 |  | AT |  | 30/11/2021 11:31 |  |
|  | : I actually don’t think; I know community engagement is very important in genomics research because genomics research is not a one-off. If I get a sample now, it could be used for very many other things in the future. [Yeah] The way I see it is this; when you look at the West, they have some long-term projects like let me say the, there's uh—I think—Okay there's a genomics project that is running in the UK where they gather data over a long period of time, in a population of patients who are born today and then they follow them for certain disorders, how they behave, over a period of 20 years or something. That is only something you can pull off when the community like let’s say when you've engaged CAB members, they understand the purpose of what genomics means, and uh can disseminate such information in their communities. So Community Advisory Boards which are normally composed of people in the community, need to be brought up to speed with genomics and its potential implications in uh—not only personalized medicine but also in public health. [Hmm, wow] So it’s important, I'll just give it to you that way. I may not expound on them here but I think people in the community need to understand that if we know what percentage of our population has the mutant verses the Worldwide variant of the same gene that could affect the response to any treatment, then that’s important for us as a country. Because that will help us know, roll out appropriate Public Health Policies, appropriate medicines that are contextual to our context—contextual to our environment sorry. But uh, yeah. So to me it’s important; engaging them, giving them basic knowledge—they don’t have to be expert level knowledge, but basic knowledge on the potential benefits of the genome project would be a good thing. | | | | | | | | | | | | | | | | |
|  |  |  |  |  |  |  |  |  |  |  |  |  |  |  |  |  |  |
|  |  |  |  |  |  |  |  |  |  |  |  |  |  |  |  |  |  |
|  |  | | | | | | | | | | | | | | | | |
|  | | | | | | | | | | | | 20 |  | AT |  | 30/11/2021 11:32 |  |
|  | : I actually don’t think; I know community engagement is very important in genomics research because genomics research is not a one-off. If I get a sample now, it could be used for very many other things in the future. [Yeah] The way I see it is this; when you look at the West, they have some long-term projects like let me say the, there's uh—I think—Okay there's a genomics project that is running in the UK where they gather data over a long period of time, in a population of patients who are born today and then they follow them for certain disorders, how they behave, over a period of 20 years or something. That is only something you can pull off when the community like let’s say when you've engaged CAB members, they understand the purpose of what genomics means, and uh can disseminate such information in their communities. So Community Advisory Boards which are normally composed of people in the community, need to be brought up to speed with genomics and its potential implications in uh—not only personalized medicine but also in public health. [Hmm, wow] So it’s important, I'll just give it to you that way. I may not expound on them here but I think people in the community need to understand that if we know what percentage of our population has the mutant verses the Worldwide variant of the same gene that could affect the response to any treatment, then that’s important for us as a country. Because that will help us know, roll out appropriate Public Health Policies, appropriate medicines that are contextual to our context—contextual to our environment sorry. But uh, yeah. So to me it’s important; engaging them, giving them basic knowledge—they don’t have to be expert level knowledge, but basic knowledge on the potential benefits of the genome project would be a good thing. | | | | | | | | | | | | | | | | |
|  |  |  |  |  |  |  |  |  |  |  |  |  |  |  |  |  |  |
|  |  |  |  |  |  |  |  |  |  |  |  |  |  |  |  |  |  |
|  |  | | | | | | | | | | | | | | | | |
|  | | | | | | | | | | | | | | | | | |
|  | | | | | | | | | | | | | | | | | |
| Reports\\Coding Summary By Code Report | | | | | | | | | | Page 29 of 249 | | | | | | | |
| 01/12/2021 00:27 | | | | | | | | | | | | | | | | | |
|  | **Nodes\\Approaches for the safe return of PG individual results\Community engagement** | | | | | | | | | | | | | | | | |
|  | | **Document** | | | | | | | | | | | | | | |  |
|  | | | **Files\\KII_Female_Researcher_4_Transcript** | | | | | | | | | | | | | |  |
|  |  |  | Yes |  |  |  | 0.0256 |  | 2 | |  | | | | | |  |
|  | | |  |  |  |  |  |  |  | |  | | | | | | |
|  | | | | | | | | | | | | 1 |  | AT |  | 30/11/2021 12:42 |  |
|  | R: Yes, the funders. Because if they are the ones going to fund the research, then you have to discuss—at least budget for that and know that, 'you know what, this is actually important and I think we should put some money aside to cover this budget line'. | | | | | | | | | | | | | | | | |
|  |  | | | | | | | | | | | | | | | | |
|  | | | | | | | | | | | | 2 |  | AT |  | 30/11/2021 12:43 |  |
|  | R: Yeah, uh. When you explain in terms of simplification of the message, lay people or maybe—you could have maybe community volunteers or people who can better simplify this message so that the participants can receive it better. So yes, it would be important actually to involve them. | | | | | | | | | | | | | | | | |
|  |  | | | | | | | | | | | | | | | | |
|  | | | **Files\\KII_Female_Researcher_5_Transcript** | | | | | | | | | | | | | |  |
|  |  |  | Yes |  |  |  | 0.0678 |  | 3 | |  | | | | | |  |
|  | | |  |  |  |  |  |  |  | |  | | | | | | |
|  | | | | | | | | | | | | 1 |  | AT |  | 30/11/2021 09:52 |  |
|  | R: Uh, Yeah. So the people in the community; the leaders and the peers will give you the context that you are saying. They will give you the context, they'll give you words for genetics, they'll tell you what is taboo [Hmm] for your setting. Like I said, even within a country Uganda, in one tribe genetics can be seen as something that is predicting your future or your past and it might be taboo [yes] to predict the future or look into your past. [Yes, hmm] So that can be taboo. So I think this understanding the context—the leaders—simple answer to...<unintelligible> I mean is community engagement necessary? Yes! Community engagement also helps you understand how your topic--disseminating results in a way that is culturally sensitive, gender, language sensitive. Yeah. | | | | | | | | | | | | | | | | |
|  |  | | | | | | | | | | | | | | | | |
|  | | | | | | | | | | | | 2 |  | AT |  | 30/11/2021 09:53 |  |
|  | Yes! Community engagement also helps you understand how your topic--disseminating results in a way that is culturally sensitive, gender, language sensitive. Yeah. | | | | | | | | | | | | | | | | |
|  | | | **Files\\KII_Female_Researcher_8_Transcript** | | | | | | | | | | | | | |  |
|  |  |  | Yes |  |  |  | 0.0080 |  | 1 | |  | | | | | |  |
|  | | |  |  |  |  |  |  |  | |  | | | | | | |
|  | | | | | | | | | | | | 1 |  | AT |  | 30/11/2021 13:50 |  |
|  | It may be hard for you to penetrate certain groups unless you are using members of their community or people that they know, they trust, leaders and what not, CAB members. Yeah, that is extremely important. | | | | | | | | | | | | | | | | |
|  |  | | | | | | | | | | | | | | | | |
|  | | | **Files\\KII_Male_Researcher_1_Transcript** | | | | | | | | | | | | | |  |
|  |  |  | Yes |  |  |  | 0.0257 |  | 1 | |  | | | | | |  |
|  | | |  |  |  |  |  |  |  | |  | | | | | | |
|  | | | | | | | | | | | | 1 |  | AT |  | 30/11/2021 15:03 |  |
|  | R: <laughs> I like the way you say that. Uhm, I think—I mean we do need to get to a point where we definitely have to engage the communities in the work we are doing, and it is not right of course for us not to uh, get the information out there in the modalities and the level of lay English or lay Luganda or whatever it is, for the patients who take part in our studies. And we just need to do it. So we just need to engage with the communities in ways that are simple for them, and there is no excuse for doing it; is what I am trying to drive to. | | | | | | | | | | | | | | | | |
|  |  | | | | | | | | | | | | | | | | |
|  | | | **Files\\KII_Male_Researcher_10_Transcript** | | | | | | | | | | | | | |  |
|  |  |  | Yes |  |  |  | 0.0745 |  | 4 | |  | | | | | |  |
|  | | |  |  |  |  |  |  |  | |  | | | | | | |
|  | | | | | | | | | | | | 1 |  | AT |  | 30/11/2021 12:27 |  |
|  | R: Yes. At community level there is a lot of benefit. [Hmm] Yes if we realized that anyway for us as Africans or as Ugandans or as this particular tribe, this seems to be something that applies to us. We are very prone to sickle cells for instance, because it’s of this common gene around us, and then people can say 'okay, maybe it is time we considered intermarrying'. Yes, at community level it can be very good. | | | | | | | | | | | | | | | | |
|  |  | | | | | | | | | | | | | | | | |
| Reports\\Coding Summary By Code Report | | | | | | | | | | Page 31 of 249 | | | | | | | |
| 01/12/2021 00:27 | | | | | | | | | | | | | | | | | |
|  | | | **Aggregate** |  | **Classification** |  | **Coverage** |  | **Number Of Coding References** | |  | **Reference Number** |  | **Coded By Initials** |  | **Modified On** |  |
|  | | | | | | | | | | | | | | | | | |
|  | | | | | | | | | | | | 2 |  | AT |  | 30/11/2021 12:29 |  |
|  | R: Yes. I'm thinking now community—we have communities of varying categories. One is our definition of community here; we are not going to go to collect the LC Council and reveals us. | | | | | | | | | | | | | | | | |
|  |  | | | | | | | | | | | | | | | | |
|  | | | | | | | | | | | | 3 |  | AT |  | 30/11/2021 12:30 |  |
|  | For me when I talk about community, if it is about ...we know that there is NDA for instance, that can receive this information on behalf of the people of the community and can take judgement on behalf of the community. [Hmm | | | | | | | | | | | | | | | | |
|  |  | | | | | | | | | | | | | | | | |
|  | | | | | | | | | | | | 4 |  | AT |  | 30/11/2021 12:30 |  |
|  | If it is something to do with cancer, with some group being more prone to cancer, the community to reveal it now will be Cancer Institute and the people involved. So that if they see someone of a particular grouping, they can look out more for what we are talking about. | | | | | | | | | | | | | | | | |
|  |  | | | | | | | | | | | | | | | | |
|  | | | **Files\\KII_Male_Researcher_2_Transcript** | | | | | | | | | | | | | |  |
|  |  |  | Yes |  |  |  | 0.0515 |  | 2 | |  | | | | | |  |
|  | | |  |  |  |  |  |  |  | |  | | | | | | |
|  | | | | | | | | | | | | 1 |  | AT |  | 30/11/2021 17:41 |  |
|  | I think if it’s a long-term cooperation that can be viewed from the point of building trust with the community, you know. But that’s the way I think I'd angle it because usually—the sponsor is usually interested in the data, usually. When they have their data, they'd have their own objectives, their own interests. This other interest is a patient interest and it comes more from a patient-advocacy point of view. | | | | | | | | | | | | | | | | |
|  |  | | | | | | | | | | | | | | | | |
|  | | | | | | | | | | | | 2 |  | AT |  | 30/11/2021 17:42 |  |
|  | So it would have to be an ethics board or a National regulator that insists on this being part of the process, otherwise if we leave it to the sponsor, I don’t think there's much value they might see in that extra expense in that particular activity. Yeah. But some might be, some might not be so it might have to be just standardized. And then the patients can decide if they are interested and if they are interested in receiving results, which results and how would they like to receive the results. Do they mind applying in a group, or they want it to be one-on-one? And then from then, it can also inform future planning because that’s also a research study of its own; the preferences of the patients and all that. After a while you can get a sense of what this community would prefer. | | | | | | | | | | | | | | | | |
|  |  | | | | | | | | | | | | | | | | |
|  | | | **Files\\KII_Male_Researcher_3_Transcript** | | | | | | | | | | | | | |  |
|  |  |  | Yes |  |  |  | 0.0091 |  | 1 | |  | | | | | |  |
|  | | |  |  |  |  |  |  |  | |  | | | | | | |
|  | | | | | | | | | | | | 1 |  | AT |  | 30/11/2021 18:03 |  |
|  | : So the need for community engagement to try and find some of these things—to try, kind of decide what will be communicated to the participants. [Hmm, hmm] there's much need for that. | | | | | | | | | | | | | | | | |
|  |  | | | | | | | | | | | | | | | | |
|  | | | **Files\\KII_Male_Researcher_6_Transcript** | | | | | | | | | | | | | |  |
|  |  |  | Yes |  |  |  | 0.0455 |  | 3 | |  | | | | | |  |
|  | | |  |  |  |  |  |  |  | |  | | | | | | |
|  | | | | | | | | | | | | 1 |  | AT |  | 30/11/2021 11:32 |  |
|  | : I actually don’t think; I know community engagement is very important in genomics research because genomics research is not a one-off. If I get a sample now, it could be used for very many other things in the future. [Yeah] The way I see it is this; when you look at the West, they have some long-term projects like let me say the, there's uh—I think—Okay there's a genomics project that is running in the UK where they gather data over a long period of time, in a population of patients who are born today and then they follow them for certain disorders, how they behave, over a period of 20 years or something. That is only something you can pull off when the community like let’s say when you've engaged CAB members, they understand the purpose of what genomics means, and uh can disseminate such information in their communities. So Community Advisory Boards which are normally composed of people in the community, need to be brought up to speed with genomics and its potential implications in uh—not only personalized medicine but also in public health. [Hmm, wow] So it’s important, I'll just give it to you that way. I may not expound on them here but I think people in the community need to understand that if we know what percentage of our population has the mutant verses the Worldwide variant of the same gene that could affect the response to any treatment, then that’s important for us as a country. Because that will help us know, roll out appropriate Public Health Policies, appropriate medicines that are contextual to our context—contextual to our environment sorry. But uh, yeah. So to me it’s important; engaging them, giving them basic knowledge—they don’t have to be expert level knowledge, but basic knowledge on the potential benefits of the genome project would be a good thing. | | | | | | | | | | | | | | | | |
|  |  |  |  |  |  |  |  |  |  |  |  |  |  |  |  |  |  |
|  |  |  |  |  |  |  |  |  |  |  |  |  |  |  |  |  |  |
|  |  | | | | | | | | | | | | | | | | |
| Reports\\Coding Summary By Code Report | | | | | | | | | | Page 32 of 249 | | | | | | | |
| 01/12/2021 00:27 | | | | | | | | | | | | | | | | | |
|  | | | **Aggregate** |  | **Classification** |  | **Coverage** |  | **Number Of Coding References** | |  | **Reference Number** |  | **Coded By Initials** |  | **Modified On** |  |
|  | | | | | | | | | | | | | | | | | |
|  | | | | | | | | | | | | 2 |  | AT |  | 30/11/2021 11:31 |  |
|  | : I actually don’t think; I know community engagement is very important in genomics research because genomics research is not a one-off. If I get a sample now, it could be used for very many other things in the future. [Yeah] The way I see it is this; when you look at the West, they have some long-term projects like let me say the, there's uh—I think—Okay there's a genomics project that is running in the UK where they gather data over a long period of time, in a population of patients who are born today and then they follow them for certain disorders, how they behave, over a period of 20 years or something. That is only something you can pull off when the community like let’s say when you've engaged CAB members, they understand the purpose of what genomics means, and uh can disseminate such information in their communities. So Community Advisory Boards which are normally composed of people in the community, need to be brought up to speed with genomics and its potential implications in uh—not only personalized medicine but also in public health. [Hmm, wow] So it’s important, I'll just give it to you that way. I may not expound on them here but I think people in the community need to understand that if we know what percentage of our population has the mutant verses the Worldwide variant of the same gene that could affect the response to any treatment, then that’s important for us as a country. Because that will help us know, roll out appropriate Public Health Policies, appropriate medicines that are contextual to our context—contextual to our environment sorry. But uh, yeah. So to me it’s important; engaging them, giving them basic knowledge—they don’t have to be expert level knowledge, but basic knowledge on the potential benefits of the genome project would be a good thing. | | | | | | | | | | | | | | | | |
|  |  |  |  |  |  |  |  |  |  |  |  |  |  |  |  |  |  |
|  |  |  |  |  |  |  |  |  |  |  |  |  |  |  |  |  |  |
|  |  | | | | | | | | | | | | | | | | |
|  | | | | | | | | | | | | 3 |  | AT |  | 30/11/2021 11:32 |  |
|  | engaging them, giving them basic knowledge—they don’t have to be expert level knowledge, but basic knowledge on the potential benefits of the genome project would be a good thing. | | | | | | | | | | | | | | | | |
|  |  | | | | | | | | | | | | | | | | |
|  | **Nodes\\Approaches for the safe return of PG individual results\Community engagement\Apply study to local context** | | | | | | | | | | | | | | | | |
|  | | **Document** | | | | | | | | | | | | | | |  |
|  | | | **Files\\KII_Female_Researcher_5_Transcript** | | | | | | | | | | | | | |  |
|  |  |  | Yes |  |  |  | 0.0295 |  | 1 | |  | | | | | |  |
|  | | |  |  |  |  |  |  |  | |  | | | | | | |
|  | | | | | | | | | | | | 1 |  | AT |  | 30/11/2021 09:52 |  |
|  | R: Uh, Yeah. So the people in the community; the leaders and the peers will give you the context that you are saying. They will give you the context, they'll give you words for genetics, they'll tell you what is taboo [Hmm] for your setting. Like I said, even within a country Uganda, in one tribe genetics can be seen as something that is predicting your future or your past and it might be taboo [yes] to predict the future or look into your past. [Yes, hmm] So that can be taboo. So I think this understanding the context—the leaders—simple answer to...<unintelligible> I mean is community engagement necessary? Yes! Community engagement also helps you understand how your topic--disseminating results in a way that is culturally sensitive, gender, language sensitive. Yeah. | | | | | | | | | | | | | | | | |
|  |  | | | | | | | | | | | | | | | | |
|  | | | | | | | | | | | | | | | | | |
|  | | | | | | | | | | | | | | | | | |
|  | | | | | | | | | | | | | | | | | |
| Reports\\Coding Summary By Code Report | | | | | | | | | | Page 33 of 249 | | | | | | | |
| 01/12/2021 00:27 | | | | | | | | | | | | | | | | | |
|  | | | **Aggregate** |  | **Classification** |  | **Coverage** |  | **Number Of Coding References** | |  | **Reference Number** |  | **Coded By Initials** |  | **Modified On** |  |
|  | **Nodes\\Approaches for the safe return of PG individual results\Community engagement\Apply study to local context\Community leaders and the peers apply study to local context** | | | | | | | | | | | | | | | | |
|  | | **Document** | | | | | | | | | | | | | | |  |
|  | | | **Files\\KII_Female_Researcher_5_Transcript** | | | | | | | | | | | | | |  |
|  |  |  | No |  |  |  | 0.0295 |  | 1 | |  | | | | | |  |
|  | | |  |  |  |  |  |  |  | |  | | | | | | |
|  | | | | | | | | | | | | 1 |  | AT |  | 30/11/2021 09:52 |  |
|  | R: Uh, Yeah. So the people in the community; the leaders and the peers will give you the context that you are saying. They will give you the context, they'll give you words for genetics, they'll tell you what is taboo [Hmm] for your setting. Like I said, even within a country Uganda, in one tribe genetics can be seen as something that is predicting your future or your past and it might be taboo [yes] to predict the future or look into your past. [Yes, hmm] So that can be taboo. So I think this understanding the context—the leaders—simple answer to...<unintelligible> I mean is community engagement necessary? Yes! Community engagement also helps you understand how your topic--disseminating results in a way that is culturally sensitive, gender, language sensitive. Yeah. | | | | | | | | | | | | | | | | |
|  |  | | | | | | | | | | | | | | | | |
|  | **Nodes\\Approaches for the safe return of PG individual results\Community engagement\Don’t reveal results to LC Council** | | | | | | | | | | | | | | | | |
|  | | **Document** | | | | | | | | | | | | | | |  |
|  | | | **Files\\KII_Male_Researcher_10_Transcript** | | | | | | | | | | | | | |  |
|  |  |  | No |  |  |  | 0.0124 |  | 1 | |  | | | | | |  |
|  | | |  |  |  |  |  |  |  | |  | | | | | | |
|  | | | | | | | | | | | | 1 |  | AT |  | 30/11/2021 12:29 |  |
|  | R: Yes. I'm thinking now community—we have communities of varying categories. One is our definition of community here; we are not going to go to collect the LC Council and reveals to us results | | | | | | | | | | | | | | | | |
|  |  | | | | | | | | | | | | | | | | |
| Reports\\Coding Summary By Code Report | | | | | | | | | | Page 34 of 249 | | | | | | | |
| 01/12/2021 00:27 | | | | | | | | | | | | | | | | | |
|  | | | **Aggregate** |  | **Classification** |  | **Coverage** |  | **Number Of Coding References** | |  | **Reference Number** |  | **Coded By Initials** |  | **Modified On** |  |
|  | **Nodes\\Approaches for the safe return of PG individual results\Community engagement\Engage Cancer Institute and the people involved in genetic work** | | | | | | | | | | | | | | | | |
|  | | **Document** | | | | | | | | | | | | | | |  |
|  | | | **Files\\KII_Male_Researcher_10_Transcript** | | | | | | | | | | | | | |  |
|  |  |  | No |  |  |  | 0.0185 |  | 1 | |  | | | | | |  |
|  | | |  |  |  |  |  |  |  | |  | | | | | | |
|  | | | | | | | | | | | | 1 |  | AT |  | 30/11/2021 12:30 |  |
|  | If it is something to do with cancer, with some group being more prone to cancer, the community to reveal it now will be Cancer Institute and the people involved. So that if they see someone of a particular grouping, they can look out more for what we are talking about. | | | | | | | | | | | | | | | | |
|  |  | | | | | | | | | | | | | | | | |
| 01/12/2021 00:27 | | | | | | | | | | | | | | | | | |
|  | | | **Aggregate** |  | **Classification** |  | **Coverage** |  | **Number Of Coding References** | |  | **Reference Number** |  | **Coded By Initials** |  | **Modified On** |  |
|  | | | **Files\\KII_Female_Researcher_7_Transcript** | | | | | | | | | | | | | |  |
|  |  |  | No |  |  |  | 0.0161 |  | 1 | |  | | | | | |  |
|  | | |  |  |  |  |  |  |  | |  | | | | | | |
|  | | | | | | | | | | | | 1 |  | AT |  | 30/11/2021 13:35 |  |
|  | R: Yeah, definitely. And I think also the good thing with funders, they usually do not want to break any codes; they want to respect the—the what, the—you know how they are always cautious about IRB in the country [Yeah] things like that. [Hmm] Yeah. So I think that once—once you know—and usually its better when it comes out like a formal body, that comes up with recommendations. For example, if UNCST came up with the recommendations, yes. Then funders might need to respect that, [Oh yeah] otherwise they always find a way...<inaudible> | | | | | | | | | | | | | | | | |
|  |  | | | | | | | | | | | | | | | | |
|  | **Nodes\\Approaches for the safe return of PG individual results\Community engagement\Engage NDA for instance, to receive this information on behalf of the people.** | | | | | | | | | | | | | | | | |
|  | | **Document** | | | | | | | | | | | | | | |  |
|  | | | **Files\\KII_Male_Researcher_10_Transcript** | | | | | | | | | | | | | |  |
|  |  |  | No |  |  |  | 0.0153 |  | 1 | |  | | | | | |  |
|  | | |  |  |  |  |  |  |  | |  | | | | | | |
|  | | | | | | | | | | | | 1 |  | AT |  | 30/11/2021 12:30 |  |
|  | For me when I talk about community, if it is about ...we know that there is NDA for instance, that can receive this information on behalf of the people of the community and can take judgement on behalf of the community. [Hmm | | | | | | | | | | | | | | | | |
|  |  | | | | | | | | | | | | | | | | |
|  | **Nodes\\Approaches for the safe return of PG individual results\Community engagement\Engage patient advocates** | | | | | | | | | | | | | | | | |
|  | | **Document** | | | | | | | | | | | | | | |  |
|  | | | **Files\\KII_Female_Researcher_7_Transcript** | | | | | | | | | | | | | |  |
|  |  |  | No |  |  |  | 0.0071 |  | 1 | |  | | | | | |  |
|  | | |  |  |  |  |  |  |  | |  | | | | | | |
|  | | | | | | | | | | | | 1 |  | AT |  | 30/11/2021 13:34 |  |
|  | R: Yeah, I think so. Usually now we are thinking of patients; even with patient advocates [Hmm] for patient safety, if we can use...<inaudible> these champions to reach...<inaudible> Yeah definitely...<unintelligible>trust at that level. | | | | | | | | | | | | | | | | |
|  |  | | | | | | | | | | | | | | | | |
|  | | | | | | | | | | | | | | | | | |
|  | | | | | | | | | | | | | | | | | |
| Reports\\Coding Summary By Code Report | | | | | | | | | | Page 36 of 249 | | | | | | | |
| 01/12/2021 00:27 | | | | | | | | | | | | | | | | | |
|  | | | **Aggregate** |  | **Classification** |  | **Coverage** |  | **Number Of Coding References** | |  | **Reference Number** |  | **Coded By Initials** |  | **Modified On** |  |
|  | | | **Files\\KII_Male_Researcher_2_Transcript** | | | | | | | | | | | | | |  |
|  |  |  | No |  |  |  | 0.0177 |  | 1 | |  | | | | | |  |
|  | | |  |  |  |  |  |  |  | |  | | | | | | |
|  | | | | | | | | | | | | 1 |  | AT |  | 30/11/2021 17:41 |  |
|  | I think if it’s a long-term cooperation that can be viewed from the point of building trust with the community, you know. But that’s the way I think I'd angle it because usually—the sponsor is usually interested in the data, usually. When they have their data, they'd have their own objectives, their own interests. This other interest is a patient interest and it comes more from a patient-advocacy point of view. | | | | | | | | | | | | | | | | |
|  |  | | | | | | | | | | | | | | | | |
|  | **Nodes\\Approaches for the safe return of PG individual results\Community engagement\Engage regulatory authority** | | | | | | | | | | | | | | | | |
|  | | **Document** | | | | | | | | | | | | | | |  |
|  | | | **Files\\KII_Male_Researcher_2_Transcript** | | | | | | | | | | | | | |  |
|  |  |  | No |  |  |  | 0.0337 |  | 1 | |  | | | | | |  |
|  | | |  |  |  |  |  |  |  | |  | | | | | | |
|  | | | | | | | | | | | | 1 |  | AT |  | 30/11/2021 17:42 |  |
|  | So it would have to be an ethics board or a National regulator that insists on this being part of the process, otherwise if we leave it to the sponsor, I don’t think there's much value they might see in that extra expense in that particular activity. Yeah. But some might be, some might not be so it might have to be just standardized. And then the patients can decide if they are interested and if they are interested in receiving results, which results and how would they like to receive the results. Do they mind applying in a group, or they want it to be one-on-one? And then from then, it can also inform future planning because that’s also a research study of its own; the preferences of the patients and all that. After a while you can get a sense of what this community would prefer. | | | | | | | | | | | | | | | | |
|  |  | | | | | | | | | | | | | | | | |
|  | **Nodes\\Approaches for the safe return of PG individual results\Community engagement\Information dissemination** | | | | | | | | | | | | | | | | |
|  | | **Document** | | | | | | | | | | | | | | |  |
|  | | | **Files\\KII_Female_Researcher_5_Transcript** | | | | | | | | | | | | | |  |
|  | | |  |  |  |  |  |  |  | |  | | | | | | |
|  | | | | | | | | | | | | 1 |  | AT |  | 30/11/2021 09:53 |  |
|  | Yes! Community engagement also helps you understand how your topic--disseminating results in a way that is culturally sensitive, gender, language sensitive. Yeah.  **Files\\ KII_Female_Researcher_8 _Transcript**  R: I think we can also utilize our peer-clients to explain these results to their fellow participants. From the time we started these studies, we have trained our expert patients how to explain and break down these terms. They even share with us some of the concerns that some participants tell them.  **Files\\ KII_Male_Researcher_11_Transcript**  R: Another way we can communicate these results to participants and community members is utilizing the creativity of our drama club here at the Institute. They usually come up with skits and songs to communicate HIV-related information at least once in two weeks. So we can give them scripts to act out something about the role of genes in breaking down the ARVs.  **Files\\ KII_Female_Researcher_2_ Transcript**  R: You can also talk about things like group education and discussions. I would think about holding group discussions among the potential participants who may be attending the ART [Antiretroviral treatment] clinic on a given day. For example, I could plan a session on one Tuesday, the day when many patients come in, then I give them some information about the study and encourage them to ask questions. This way, some other potential participants can learn from others’ questions but can ask more questions when they choose to join the | | | | | | | | | | | | | | | | |
|  |  | | | | | | | | | | | | | | | | |
|  | | | | | | | | | | | | | | | | | |
| Reports\\Coding Summary By Code Report | | | | | | | | | | Page 37 of 249 | | | | | | | |
| 01/12/2021 00:27 | | | | | | | | | | | | | | | | | |
|  | | | **Aggregate** |  | **Classification** |  | **Coverage** |  | **Number Of Coding References** | |  | **Reference Number** |  | **Coded By Initials** |  | **Modified On** |  |
|  | | | **Files\\KII_Female_Researcher_8_Transcript** | | | | | | | | | | | | | |  |
|  |  |  | Yes |  |  |  | 0.0080 |  | 1 | |  | | | | | |  |
|  | | |  |  |  |  |  |  |  | |  | | | | | | |
|  | | | | | | | | | | | | 1 |  | AT |  | 30/11/2021 13:50 |  |
|  | It may be hard for you to penetrate certain groups unless you are using members of their community or people that they know, they trust, leaders and what not, CAB members. Yeah, that is extremely important. | | | | | | | | | | | | | | | | |
|  |  | | | | | | | | | | | | | | | | |
|  | | | **Files\\KII_Male_Researcher_6_Transcript** | | | | | | | | | | | | | |  |
|  |  |  | Yes |  |  |  | 0.0455 |  | 3 | |  | | | | | |  |
|  | | |  |  |  |  |  |  |  | |  | | | | | | |
|  | | | | | | | | | | | | 1 |  | AT |  | 30/11/2021 11:32 |  |
|  | : I actually don’t think; I know community engagement is very important in genomics research because genomics research is not a one-off. If I get a sample now, it could be used for very many other things in the future. [Yeah] The way I see it is this; when you look at the West, they have some long-term projects like let me say the, there's uh—I think—Okay there's a genomics project that is running in the UK where they gather data over a long period of time, in a population of patients who are born today and then they follow them for certain disorders, how they behave, over a period of 20 years or something. That is only something you can pull off when the community like let’s say when you've engaged CAB members, they understand the purpose of what genomics means, and uh can disseminate such information in their communities. So Community Advisory Boards which are normally composed of people in the community, need to be brought up to speed with genomics and its potential implications in uh—not only personalized medicine but also in public health. [Hmm, wow] So it’s important, I'll just give it to you that way. I may not expound on them here but I think people in the community need to understand that if we know what percentage of our population has the mutant verses the Worldwide variant of the same gene that could affect the response to any treatment, then that’s important for us as a country. Because that will help us know, roll out appropriate Public Health Policies, appropriate medicines that are contextual to our context—contextual to our environment sorry. But uh, yeah. So to me it’s important; engaging them, giving them basic knowledge—they don’t have to be expert level knowledge, but basic knowledge on the potential benefits of the genome project would be a good thing. | | | | | | | | | | | | | | | | |
|  |  |  |  |  |  |  |  |  |  |  |  |  |  |  |  |  |  |
|  |  |  |  |  |  |  |  |  |  |  |  |  |  |  |  |  |  |
|  |  | | | | | | | | | | | | | | | | |
|  | | | | | | | | | | | | 2 |  | AT |  | 30/11/2021 11:31 |  |
|  | : I actually don’t think; I know community engagement is very important in genomics research because genomics research is not a one-off. If I get a sample now, it could be used for very many other things in the future. [Yeah] The way I see it is this; when you look at the West, they have some long-term projects like let me say the, there's uh—I think—Okay there's a genomics project that is running in the UK where they gather data over a long period of time, in a population of patients who are born today and then they follow them for certain disorders, how they behave, over a period of 20 years or something. That is only something you can pull off when the community like let’s say when you've engaged CAB members, they understand the purpose of what genomics means, and uh can disseminate such information in their communities. So Community Advisory Boards which are normally composed of people in the community, need to be brought up to speed with genomics and its potential implications in uh—not only personalized medicine but also in public health. [Hmm, wow] So it’s important, I'll just give it to you that way. I may not expound on them here but I think people in the community need to understand that if we know what percentage of our population has the mutant verses the Worldwide variant of the same gene that could affect the response to any treatment, then that’s important for us as a country. Because that will help us know, roll out appropriate Public Health Policies, appropriate medicines that are contextual to our context—contextual to our environment sorry. But uh, yeah. So to me it’s important; engaging them, giving them basic knowledge—they don’t have to be expert level knowledge, but basic knowledge on the potential benefits of the genome project would be a good thing. | | | | | | | | | | | | | | | | |
|  |  |  |  |  |  |  |  |  |  |  |  |  |  |  |  |  |  |
|  |  |  |  |  |  |  |  |  |  |  |  |  |  |  |  |  |  |
|  |  | | | | | | | | | | | | | | | | |
|  | | | | | | | | | | | | 3 |  | AT |  | 30/11/2021 11:32 |  |
|  | engaging them, giving them basic knowledge—they don’t have to be expert level knowledge, but basic knowledge on the potential benefits of the genome project would be a good thing. | | | | | | | | | | | | | | | | |
| Reports\\Coding Summary By Code Report | | | | | | | | | | Page 38 of 249 | | | | | | | |
| 01/12/2021 00:27 | | | | | | | | | | | | | | | | | |
|  | | | **Aggregate** |  | **Classification** |  | **Coverage** |  | **Number Of Coding References** | |  | **Reference Number** |  | **Coded By Initials** |  | **Modified On** |  |
|  | **Nodes\\Approaches for the safe return of PG individual results\Community engagement\Information dissemination\CAB members can disseminate genetic information in their communities** | | | | | | | | | | | | | | | | |
|  | | **Document** | | | | | | | | | | | | | | |  |
|  | | | **Files\\KII_Female_Researcher_8_Transcript** | | | | | | | | | | | | | |  |
|  |  |  | No |  |  |  | 0.0080 |  | 1 | |  | | | | | |  |
|  | | |  |  |  |  |  |  |  | |  | | | | | | |
|  | | | | | | | | | | | | 1 |  | AT |  | 30/11/2021 13:50 |  |
|  | It may be hard for you to penetrate certain groups unless you are using members of their community or people that they know, they trust, leaders and what not, CAB members. Yeah, that is extremely important. | | | | | | | | | | | | | | | | |
| Reports\\Coding Summary By Code Report | | | | | | | | | | Page 39 of 249 | | | | | | | |
| 01/12/2021 00:27 | | | | | | | | | | | | | | | | | |
|  | | | **Aggregate** |  | **Classification** |  | **Coverage** |  | **Number Of Coding References** | |  | **Reference Number** |  | **Coded By Initials** |  | **Modified On** |  |
|  | **Nodes\\Approaches for the safe return of PG individual results\Community engagement\Information dissemination\People in the community need to understand about what percentage of our population has the mutant variant of the same gene.** | | | | | | | | | | | | | | | | |
|  | | **Document** | | | | | | | | | | | | | | |  |
|  | | | **Files\\KII_Male_Researcher_6_Transcript** | | | | | | | | | | | | | |  |
|  |  |  | No |  |  |  | 0.0455 |  | 1 | |  | | | | | |  |
|  | | |  |  |  |  |  |  |  | |  | | | | | | |
|  | | | | | | | | | | | | 1 |  | AT |  | 30/11/2021 11:32 |  |
|  | : I actually don’t think; I know community engagement is very important in genomics research because genomics research is not a one-off. If I get a sample now, it could be used for very many other things in the future. [Yeah] The way I see it is this; when you look at the West, they have some long-term projects like let me say the, there's uh—I think—Okay there's a genomics project that is running in the UK where they gather data over a long period of time, in a population of patients who are born today and then they follow them for certain disorders, how they behave, over a period of 20 years or something. That is only something you can pull off when the community like let’s say when you've engaged CAB members, they understand the purpose of what genomics means, and uh can disseminate such information in their communities. So Community Advisory Boards which are normally composed of people in the community, need to be brought up to speed with genomics and its potential implications in uh—not only personalized medicine but also in public health. [Hmm, wow] So it’s important, I'll just give it to you that way. I may not expound on them here but I think people in the community need to understand that if we know what percentage of our population has the mutant verses the Worldwide variant of the same gene that could affect the response to any treatment, then that’s important for us as a country. Because that will help us know, roll out appropriate Public Health Policies, appropriate medicines that are contextual to our context—contextual to our environment sorry. But uh, yeah. So to me it’s important; engaging them, giving them basic knowledge—they don’t have to be expert level knowledge, but basic knowledge on the potential benefits of the genome project would be a good thing. | | | | | | | | | | | | | | | | |
|  |  |  |  |  |  |  |  |  |  |  |  |  |  |  |  |  |  |
|  |  |  |  |  |  |  |  |  |  |  |  |  |  |  |  |  |  |
|  |  | | | | | | | | | | | | | | | | |
|  | **Nodes\\Approaches for the safe return of PG individual results\Community engagement\challenges in results dissemination** | | | | | | | | | | | | | | | | |
|  | | **Document** | | | | | | | | | | | | | | |  |
|  | | | **Files\\KII_Female_Researcher_5_Transcript** | | | | | | | | | | | | | |  |
|  |  |  | No |  |  |  | 0.0062 |  | 1 | |  | | | | | |  |
|  | | |  |  |  |  |  |  |  | |  | | | | | | |
|  | | | | | | | | | | | | 1 |  | AT |  | 30/11/2021 09:53 |  |
|  | Yes! Community engagement also helps you understand how your topic--disseminating results in a way that is culturally sensitive, gender, language sensitive. Yeah. But one of the biggest challenge we have here is that we don’t have trained genetic counsellors who are well trained to disseminate these results and give appropriate counselling to our people | | | | | | | | | | | | | | | | |
|  |  | | | | | | | | | | | | | | | | |
|  | | | | | | | | | | | | | | | | | |
|  | | | | | | | | | | | | | | | | | |
| Reports\\Coding Summary By Code Report | | | | | | | | | | Page 40 of 249 | | | | | | | |
| 01/12/2021 00:27 | | | | | | | | | | | | | | | | | |
|  | | | **Aggregate** |  | **Classification** |  | **Coverage** |  | **Number Of Coding References** | |  | **Reference Number** |  | **Coded By Initials** |  | **Modified On** |  |
|  | | | **Files\\KII_Male_Researcher_6_Transcript** | | | | | | | | | | | | | |  |
|  |  |  | No |  |  |  | 0.0455 |  | 1 | |  | | | | | |  |
|  | | |  |  |  |  |  |  |  | |  | | | | | | |
|  | | | | | | | | | | | | 1 |  | AT |  | 30/11/2021 11:31 |  |
|  | : I actually don’t think; I know community engagement is very important in genomics research because genomics research is not a one-off. If I get a sample now, it could be used for very many other things in the future. [Yeah] The way I see it is this; when you look at the West, they have some long-term projects like let me say the, there's uh—I think—Okay there's a genomics project that is running in the UK where they gather data over a long period of time, in a population of patients who are born today and then they follow them for certain disorders, how they behave, over a period of 20 years or something. That is only something you can pull off when the community like let’s say when you've engaged CAB members, they understand the purpose of what genomics means, and uh can disseminate such information in their communities. So Community Advisory Boards which are normally composed of people in the community, need to be brought up to speed with genomics and its potential implications in uh—not only personalized medicine but also in public health. [Hmm, wow] So it’s important, I'll just give it to you that way. I may not expound on them here but I think people in the community need to understand that if we know what percentage of our population has the mutant verses the Worldwide variant of the same gene that could affect the response to any treatment, then that’s important for us as a country. Because that will help us know, roll out appropriate Public Health Policies, appropriate medicines that are contextual to our context—contextual to our environment sorry. But uh, yeah. So to me it’s important; engaging them, giving them basic knowledge—they don’t have to be expert level knowledge, but basic knowledge on the potential benefits of the genome project would be a good thing. | | | | | | | | | | | | | | | | |
|  |  |  |  |  |  |  |  |  |  |  |  |  |  |  |  |  |  |
|  |  |  |  |  |  |  |  |  |  |  |  |  |  |  |  |  |  |
|  |  | | | | | | | | | | | | | | | | |
|  | **Nodes\\Approaches for the safe return of PG individual results\Community engagement\simplify genetic message** | | | | | | | | | | | | | | | | |
|  | | **Document** | | | | | | | | | | | | | | |  |
|  | | | **Files\\KII_Female_Researcher_4_Transcript** | | | | | | | | | | | | | |  |
|  |  |  | No |  |  |  | 0.0134 |  | 1 | |  | | | | | |  |
|  | | |  |  |  |  |  |  |  | |  | | | | | | |
|  | | | | | | | | | | | | 1 |  | AT |  | 30/11/2021 12:43 |  |
|  | R: Yeah, uh. When you explain in terms of simplification of the message, lay people or maybe—you could have maybe community volunteers or people who can better simplify this message so that the participants can receive it better. So yes, it would be important actually to involve them. | | | | | | | | | | | | | | | | |
|  |  | | | | | | | | | | | | | | | | |
|  | | | **Files\\KII_Male_Researcher_1_Transcript** | | | | | | | | | | | | | |  |
|  |  |  | No |  |  |  | 0.0257 |  | 1 | |  | | | | | |  |
|  | | |  |  |  |  |  |  |  | |  | | | | | | |
|  | | | | | | | | | | | | 1 |  | AT |  | 30/11/2021 15:03 |  |
|  | R: <laughs> I like the way you say that. Uhm, I think—I mean we do need to get to a point where we definitely have to engage the communities in the work we are doing, and it is not right of course for us not to uh, get the information out there in the modalities and the level of lay English or lay Luganda or whatever it is, for the patients who take part in our studies. And we just need to do it. So we just need to engage with the communities in ways that are simple for them, and there is no excuse for doing it; is what I am trying to drive to. | | | | | | | | | | | | | | | | |
|  |  | | | | | | | | | | | | | | | | |
|  | | | | | | | | | | | | | | | | | |
| Reports\\Coding Summary By Code Report | | | | | | | | | | Page 41 of 249 | | | | | | | |
| 01/12/2021 00:27 | | | | | | | | | | | | | | | | | |
|  |  | | | | | | | | | | | | | | | | |
|  |  | | | | | | | | | | | | | | | | |
|  | **Nodes\\Approaches for the safe return of PG individual results\role of researchers\roles of research institutions\ roles of participants** | | | | | | | | | | | | | | | | |
|  | | **Document** | | | | | | | | | | | | | | |  |
|  | | | **Files\\KII_Female_Researcher_8_Transcript** | | | | | | | | | | | | | |  |

What we [researchers] should do is to understand the implications of these results first, before presenting them to the participants, or to their family members. This way, we shall be able to provide answers and appropriate solutions to participants and their family members. (KII_Male_Researcher_6)

**Files\\KII_Male_Researcher_12_Transcript**

“We have the moral obligation to share all these findings with the Regulatory Authorities and ethics committees, whether primary results information or incidental findings. This information can help guide policy makers on what they may think is crucial to consider about pharmacogenomics in HIV treatment or other genomic studies.

**Files\\KII_Female_Researcher_5_Transcript**

One thing I would request research institutions to do, is to establish collaborations with other health facilities or NGOs that can provide extra support to our participants and their family members after we share these results. You may find that some participants need extra psychological support than just the counseling services we offer here.

**Files\\KII_Female_Researcher_3_Transcript**

We try our level best to build a good relationship with our participants so that they can freely and openly tell us their concerns and aspects they might not have understood. So we expect them to ask questions and also tell us truthful information during our discussions with them

**Files\\KII_Male_Researcher_10_Transcript**

I think research participants have the responsibility to clearly inform the researchers whether they want to receive their results or not. They should also specify the kind of results they would like to receive. They should also let us know whether it is okay to share their results with family members or not
