# Supplementary material for: The role of community engagement in promoting research participants’ understanding of pharmacogenomic research results: Perspectives of stakeholders involved in HIV/AIDS research and treatment
Source: PLoS One. 2024 Apr 2;19(4):e0299081. doi: 10.1371/journal.pone.0299081 (PMC10986979; doi:10.1371/journal.pone.0299081)
Supplement: Attachment — Submitted filename: Responses to reviewers.docx [file pone.0299081.s004.docx]

**RESPONSE TO ADDITIONAL JOURNAL REQUIREMENTS**

| **ID** | **COMMENTS** | **RESPONSES** |
| --- | --- | --- |
| **01** | Please ensure that your manuscript meets PLOS ONE's style requirements, including those for file naming. The PLOS ONE style templates can be found at  <https://journals.plos.org/plosone/s/file?id=wjVg/PLOSOne_formatting_sample_main_body.pdf> and  <https://journals.plos.org/plosone/s/file?id=ba62/PLOSOne_formatting_sample_title_authors_affiliations.pdf> | Thank you very much for raising this comment and providing the links to the style templates. We have revised the format of the manuscript as guided. |
| **02** | We suggest you thoroughly copyedit your manuscript for language usage, spelling, and grammar. If you do not know anyone who can help you do this, you may wish to consider employing a professional scientific editing service.  Upon resubmission, please provide the following:  The name of the colleague or the details of the professional service that edited your manuscript | Thank you very much for raising this comment. We acknowledge that the version of the manuscript that was initially submitted had several spelling and grammatical errors. However, the authors have proof-read the revised manuscript and also sought an independent review from a colleague, Dr. Jerome Roy Semakula who recently excelled in his IELTS exam. |
| **03** | We note that you have provided funding information that is currently declared in your Funding Statement. However, funding information should not appear in the Acknowledgments section or other areas of your manuscript. We will only publish funding information present in the Funding Statement section of the online submission form.  Please remove any funding-related text from the manuscript and let us know how you would like to update your Funding Statement. | Thank you very much for raising this comment. We have deleted the funding information from the acknowledgment section in the revised manuscript. Thank you once again for the correction.  We request to maintain the funding information in the Funding Statement section of the online submission form |
| **04** | In your Data Availability statement, you have not specified where the minimal data set underlying the results described in your manuscript can be found. PLOS defines a study's minimal data set as the underlying data used to reach the conclusions drawn in the manuscript and any additional data required to replicate the reported study findings in their entirety. | Thank you for raising this comment. We appreciate the spirit of sharing data for replication of the reported study findings and other benefits of data sharing. The data has been reported as quotes in the results section of the manuscript. This is the minimal data set used to reach the conclusions described in the manuscript. |
| **05** | We note that you have stated that you will provide repository information for your data at acceptance. Should your manuscript be accepted for publication, we will hold it until you provide the relevant accession numbers or DOIs necessary to access your data. If you wish to make changes to your Data Availability statement, please describe these changes in your cover letter and we will update your Data Availability statement to reflect the information you provide. | Thank you very much for raising this concern. I have provided the minimal data set as quotes reported in the results section of the manuscript. |

**RESPONSES TO PEER REVIEW COMMENTS**

| **ID** | **COMMENT** | **RESPONSES** | **Page** | **Line** |
| --- | --- | --- | --- | --- |
| **01** | Differentiate with responses. When I conduct focus groups, I usually have a design that differentiates people's roles to different focus groups. This way I can look at comparisons across groups with norms and concerns. You've presented the data as if all respondents - researchers, ethics committee members, and community members - all agree across every issue. | Thank you very for raising this comment. This comment has helped us realize that we needed to clarify how the data were collected across the different study populations. We conducted focus group discussions with community representatives from five research institutions offering care and treatment to people living with HI. We also conducted key informant interviews with researchers involved in pharmacogenomic research and HIV treatment and in-depth interviews with members of research ethics committees who had prior experience in reviewing pharmacogenomic research for HIV treatment. We do agree with you that all the above categories of respondents have different roles and that their feedback has been presented with their roles. We have followed your recommendation on how to present the results and the discussion of our findings. The contributions from each category of the respondents have been well labelled at the end of each quote in the results section. | 16 -29 | NA |
| **02** | Identify what is important in the study and lean into it. You're providing stories on how various actors feel about concepts of community engagement. The report back on findings seems dry, and basically is reduced to "Community Engagement is important." Why and how are key questions that you do not approach directly. | Thank you very much for raising this comment. We have followed your recommendation to focus on the research question when discussing our findings. We have provided detailed information to why and how community engagement plays an important role in promoting participants’ understanding of pharmacogenomic research results. | 29-33 | N/A |
| **03** | Avoid jargon. While you discuss pharmacogenomic research, it is often jargon. What makes this kind of research important for people living with HIV and who decides it's important? What are the ramifications of the research? There are times when you seem to conflate research with clinical practice (e.g. the individual wants their data from research). How is this possible? You make an assumption that all readers are up to date in this part of the field. | Thank you very much for raising this comment. This comment has helped us realize that we needed to revise our discussion section. We have provided more detail about the importance of pharmacogenomic research to PLHIV and the researchers involved in the treatment of HIV/AIDS. We have also provided details on the ramifications of this research and tried to explain what pharmacogenomic research is to avoid jargon. | 29-33 | N/A |
| **04** | Barriers to Community Engagement are not identified. This is odd, as most researchers will discuss their frustrations openly (e.g. funding requirements, time, lack of expertise) yet there was little of that here (outside of language). Other barriers could be highlighted more. | We have also provided information about other challenges that researchers experience when engaging communities in addition to some of them that were already mentioned. For example the absence of genetic counsellors who may be the most suitable professionals to determine the safe approaches and methods of communicating genomic and pharmacogenomic research information in different communities. In addition, we have added information about the limited funding for community engagement activities in some research institutions. | 32 | 624-625 |
| **05** | Please remove any funding-related text from the manuscript and let us know how you would like to update your Funding Statement | Thank you very much for your feedback. We have revised the acknowledgment section as advised. | 34 | 670-672 |
